# Repositioning the Leader Peptide in Graspetide Biosynthesis

**DOI:** 10.1021/jacs.5c21135

**Published:** 2026-03-06

**Authors:** Toby G. Johnson, Dean M. Miller, Drew V. Carson, Brian Choi, A. James Link

**Affiliations:** † Department of Chemical and Biological Engineering, 6740 Princeton University, Princeton, New Jersey 08544, United States; ‡ Department of Chemistry, Princeton University, Princeton, New Jersey 08544, United States; § Department of Molecular Biology, Princeton University, Princeton, New Jersey 08544, United States; £ Omenn-Darling Bioengineering Institute, Princeton University, Princeton, New Jersey 08544, United States

## Abstract

Applying enzymes from unique biosynthetic pathways in a combinatorial fashion to garner new-to-nature products has been a long-standing goal of synthetic biology. Ribosomally synthesized and post-translationally modified peptides (RiPPs) are a family of natural products that comprise a broad array of chemical and structural diversity, including mechanically interlocked molecular architectures. Guided by supramolecular recognition of an N-terminal leader sequence, the core region of a precursor peptide is decorated by tailoring enzymes to generate a mature RiPP. Through repositioning the leader sequence of the fuscimiditide precursor peptide C-terminal to the core, we have shown that substrate-selective post-translational modification by the graspetide synthetase, ThfB, is retained *in cellulo* and *in vitro*. Reconstitution of the ThfB-mediated cyclization of precursors with the native N-terminal or repositioned C-terminal leader sequence *in vitro* revealed a modest 2-fold reduction in the rate of enzymatic modification upon repositioning the leader. Rearrangement of the precursor peptide enabled the generation of chimeric RiPP products that were decorated by both lasso peptide and graspetide family enzymes guided by two leader sequences (one N-terminal and one C-terminal). This leader peptide engineering strategy unlocks access to mechanically interlocked peptide products with post-translational modifications not seen in nature, expanding the structural diversity possible in RiPPs.

## Graphical abstract

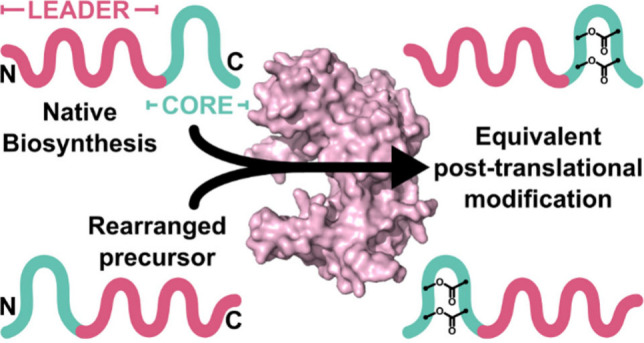

## Introduction

A diverse array of chemical complexity is biosynthesized within cells. Several classes of biosynthetic pathways exist and generally produce a single target natural product. Ribosomally synthesized and post-translationally modified peptides (RiPPs) represent a family of natural products which are inherently encoded for within an organism’s genetic code.^1^ A target RiPP is genetically encoded as the core region of a precursor peptide within a biosynthetic gene cluster (BGC), which also contains the genes for tailoring enzymes that post-translationally modify (PTM) the precursor peptide. The tailoring enzymes of the BGC selectively decorate the core peptide, guided by supramolecular recognition of an N-terminal leader peptide (leader-dependent) or recognition of the proteolytically cleaved core peptide (leader-independent).^2,3^ Through binding the leader peptide, leader-dependent enzymes achieve high substrate specificity, but can display substrate promiscuity within the core sequence, enabling the preparation of engineered constructs.^4–7^ Leader sequences tend to be highly substrate specific, where each peptide within a RiPP family will have a unique leader sequence and correspondingly a bespoke set of tailoring enzymes for peptide maturation.^8–10^ Naturally occurring lasso peptide precursor peptides without an N-terminal leader sequence are known, which rely on an endogenous methionyl aminopeptidase to excise the first Met and are functionalized by leader-independent tailoring enzymes.^11,12^

Engineering RiPPs through core sequence substitution has now become commonplace,^13–16^ supporting structural characterization,^17–19^ enabling bioactivity screening of vast libraries,^20–22^ and allowing integration of unnatural amino acids^23–26^ and the generation of new-to-nature interlocked peptide architectures.^27,28^ Studies that engineer the leader sequence of a RiPP precursor typically are aimed at identifying the key residues (recognition sequence) involved in binding interactions with the tailoring enzymes.^29–31^ Interestingly, for some RiPPs much of the leader sequence has been shown to be dispensable, with N-terminally truncated precursors being fully modified by the tailoring enzymes as long as the recognition sequence was not removed.^18,32^ Varying the leader through truncations or substitutions does, however, typically reduce enzyme modification efficiency and can alter the location of PTMs.^33^

In the pursuit of ever more complex and highly decorated peptides, it would be desirable to employ tailoring enzymes in a combinatorial fashion from different classes of RiPPs.^34^ Appending the leader sequence to the tailoring enzyme (in *cis*) has been shown to generate a constitutively active enzyme that modifies the core peptide without a leader.^35–39^ Addition of the leader sequence as a separate peptide (in *trans*)^37,38^ is an alternative method to activate the native tailoring enzyme to modify a core peptide lacking the leader sequence *in vitro*. Swapping the leader sequence iteratively after enzyme modification enabled sequential functionalization of a core sequence; however, this strategy has only been demonstrated *in vitro*.^40^ Although these strategies have been shown to be effective at generating modified substrates, the reduced size of the precursor peptide negates the study of peptide biosynthesis *in cellulo*, as the smaller precursor peptide is more prone to proteolysis. Sequentially linking multiple leader sequences or merging key recognition sequences into one N-terminal leader has enabled the recruitment of multiple enzymes to act on a single core sequence both *in vitro* ^41–43^ and *in cellulo*.^44–46^ Very recently, the binding interactions and modification selectivities of a library of nine enzymes have been parametrized to enable the *de novo* design of precursor peptides that recruit enzymes from the library to furnish a hybrid RiPP *in cellulo*.^47^ However, these approaches are all limited by the sequence space available to encode recognition sequences within an N-terminal leader sequence, equivalent to natural biosynthesis.

The diverse chemistry, broad biochemical activities, and genetically encoded nature of RiPPs make them an attractive class of natural products for engineering attempts. Fuscimiditide is an aspartimidylated graspetide (graspimiditide)^48^ encoded in the genome of the thermophilic actinobacterium *Thermobifida fusca*.^18,49^ The structure, as shown in Figure 1, comprises two Thr-Asp side chain ester cross-links installed by the ATP-grasp enzyme, ThfB, generating a bicyclic stem-loop structure. An aspartimide moiety in the loop is enzymatically installed by the *O*-methyltransferase ThfM, which shares homology with the protein repair catalyst protein l-isoaspartyl methyltransferase (PIMT). The fuscimiditide precursor, ThfA, is composed of a 67 aa leader peptide followed by a 22 aa core peptide where the ester and aspartimide modifications occur.

**Figure 1:**
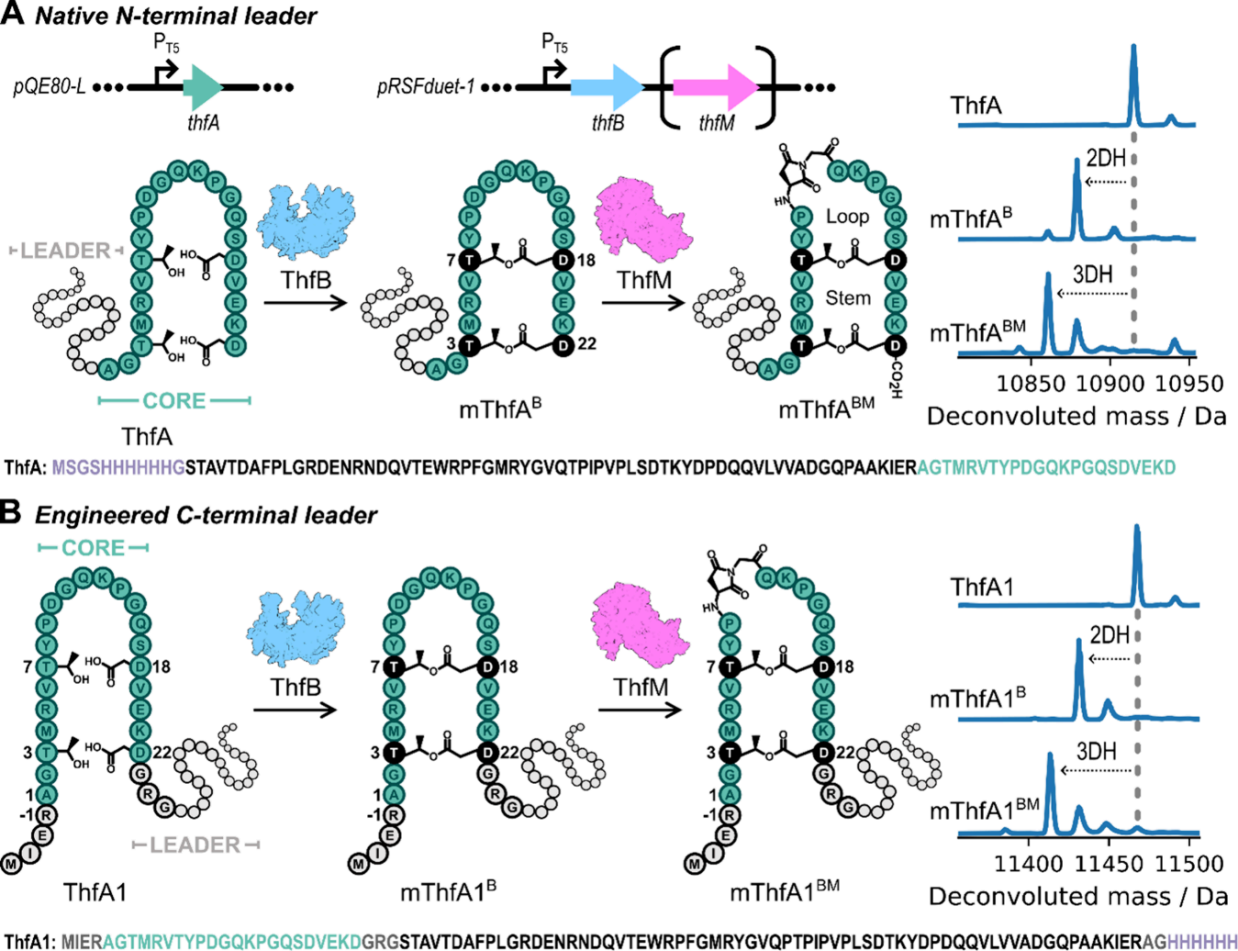
Engineering the biosynthesis of a RiPP precursor by repositioning the N-terminal leader sequence to the C-terminus. A: Native biosynthesis of fuscimiditide. The ribosomally synthesized linear precursor peptide ThfA is composed of an N-terminal leader sequence (gray) and a C-terminal core region (green). ThfA is phosphorylated by the ATP-grasp enzyme ThfB, which installs two ester cross-links, resulting in 2-fold dehydration of the precursor. mThfA^B^ is then methylated by the *O*-methyltransferase, ThfM, promoting intramolecular cyclization to form an aspartimide in mThfA^BM^, with another net dehydration. B: The engineered precursor ThfA1, with a C-terminal leader sequence (retaining the N–C order of aa) was efficiently modified by both ThfB and ThfM *in cellulo*, resulting in 2- and 3-fold dehydrations, respectively.

Herein, we demonstrate that repositioning the 67 aa leader peptide of ThfA from the N-terminus to the C-terminus (i.e., converting the leader into a follower sequence) results in the maturation of a peptide with the same side chain cross-links and subsequent aspartimidylation as the native peptide. Since the N-terminus of the precursor peptide is now free for functionalization, we demonstrated that chimeric natural products may be biosynthesized by combining functionalities from graspetide and lasso peptide RiPP families into a single peptide fusion containing 5 post-translational modifications, installed by four different tailoring enzymes. Reducing the size of the leader peptide or repositioning it entirely to free the N-terminus of the RiPP precursor enables new strategies for the further bioengineering of hybrid RiPPs.

## Results and Discussion

### Swapping the ThfA Leader Peptide To Be a Follower

Through core peptide engineering, we have previously shown ThfB to be a highly promiscuous graspetide synthetase, enabling the generation of multicyclic peptides, cyclized proteins, and cross-linked protein dimers *in cellulo*.^50,51^ This success in engineering alternative ThfA precursors for ThfB-mediated cyclization inspired us to investigate whether the leader sequence could be moved to a follower. Rearranged precursor peptides of ThfA C76A (to eliminate disulfide formation, termed ThfA hereafter) were designed with the leader sequence repositioned from the N-terminus to the C-terminus, to evaluate whether the tailoring enzymes, ThfB and ThfM, would still be recruited by the precursor and catalyze peptide modification. Genes encoding the rearranged His-tagged precursor peptides of ThfA were prepared via Golden Gate assembly and cloned into pQE80-L vectors (see the Supporting Information for full cloning details). Coexpression of ThfA variants with the tailoring enzymes ThfB and ThfM (encoded for on a separate pRSFDuet-1 vector) in *E. coli* BL21 (DE3) *ΔslyD* cells allowed the extent of modification to be quantified by LC-MS analysis following Ni-NTA affinity chromatography of the cell lysate. Natively, ThfB mediates the phosphorylation of an Asp carboxylic acid on ThfA, forming a mixed anhydride activated to nucleophilic attack by a Thr hydroxyl that results in the formation of an ester cross-link. Twofold dehydration of ThfA, when coexpressed with ThfB, is indicative of the formation of two side chain–side chain ester cross-links (mThfA^B^, Figure 1A). Subsequently, ThfM methylates an Asp carboxylic acid on mThfA^B^, activating it to nucleophilic attack by a backbone amide nitrogen of the adjacent aa to form an aspartimide, leading to one further net dehydration of the precursor peptide (mThfA^BM^, Figure 1A). ThfM was previously shown to modify the precursor in a leader-independent fashion.^18^ Reconstituted *in vitro* reactions with ThfM demonstrated that the cyclized core peptide lacking a leader sequence was modified, but linear full-length or linear core peptide precursors were not. ThfM therefore is guided by the cross-linking state of the precursor, only modifying the cyclized core peptide.

The variant ThfA1 was designed with a truncated N-terminal leader sequence, retaining only three amino acids prior to the core sequence. A linker sequence followed by the leader sequence (retaining the native order of aa) was repositioned to the C-terminus of the core sequence, which natively would be the end of the precursor peptide. Finally, a C-terminal His_6_ tag was included in the construct to enable efficient isolation of the redesigned precursor peptide by Ni-affinity chromatography. Expression of ThfA1 without the tailoring enzymes gave a product with no dehydration (Figure 1B, Figure S4), as expected. However, upon coexpression of ThfA1 with the graspetide synthetase (ThfB) *in cellulo*, the major product observed was 2-fold dehydrated (Figure 1B, Figure S5), highlighting that modifications were enzymatically installed by ThfB. A further dehydration of the precursor was observed when ThfA1 was coexpressed with ThfB and ThfM (Figure 1B, Figure S6), but not when coexpressed with only ThfM (Figure S7), suggesting that the cross-links installed in mThfA1^B^ are in the native positions (Thr3-Asp22 and Thr7-Asp18), as ThfM modification is based on recognition of the bicyclic core peptide structure.^18^ Variants ThfA2–4 with cross-linking pairs substituted to unreactive residues (Thr to Val and Asp to Asn) were generated to probe the ester connectivity, with ThfA2 harboring Thr3Val and Asp22Asn substitutions and ThfA3 harboring Thr7Val and Asp18Asn substitutions. ThfA4 harbors all four substitutions. When coexpressed with ThfB, these variants showed a single dehydration (mThfA2^B^ and mThfA3^B^, Figures S8, S9) or no dehydrations (ThfA4, Figure S10) with one or two cross-linking pairs ablated, respectively.

The GRG linker in ThfA1 was designed to introduce a trypsin cleavage site into the precursor to allow targeted proteolysis between the core peptide and the C-terminal leader sequence. Analysis of the trypsin digestion products of mThfA1^B^ revealed that the dehydrations were localized to the core region of the precursor peptide (Figure S11). Dehydrated species were observed only in peptide fragments containing the core sequence with a characteristic fragment (1–24 aa) observed with two dehydrations. Interestingly, this 1–24 aa 2-fold dehydrated fragment displayed resistance to tryptic digestion despite containing multiple trypsin cleavage sites, suggesting a cyclic structure with increased proteolytic stability. MS/MS analysis of the 1–24 aa 2-fold dehydrated fragment is also supportive of a bicyclic structure with cross-links at the same positions as the native product (Figure S12). HPLC purification of this tryptic fragment and incubation with hydrazine resulted in the hydrazinolysis of the esters and tagging of the Asp residues involved in the cross-link. MS/MS analysis of this hydrazinolysis product confirmed that the native Asp18 and Asp22 residues were cross-linked in mThfA1^B^ (Figure S13). Changing the GRG linker to GS in precursor ThfA5 generated a similar product distribution when coexpressed with ThfB *in cellulo* (Figures S14, S15), with 2-fold dehydration of mThfA5^B^ being the major product. Substitution of position Ala-62′ in the leader sequence back to the native cysteine (ThfA6) did not alter the distribution of ThfB-modified products (Figure S16). Collectively, these results demonstrate the ability to reposition the leader sequence from the N-terminus to the C-terminus and generate the native modifications in a RiPP biosynthetic pathway *in cellulo*, revealing for the first time the portability of the leader sequence within a precursor peptide.

To investigate the impact of repositioning the leader sequence to the C-terminus, the reaction rate of *in vitro* reconstituted ThfB-mediated cyclization was monitored. N-Terminal His_6_ tagged ThfB was natively purified for *in vitro* reactions. ThfA1 (10 μM) was incubated with ThfB (1 μM, 0.1 equiv) at 37 °C in sodium phosphate buffer (100 mM, pH 6.8) in the presence of ATP (10 mM), MgCl_2_ (10 mM), dithiothreitol (10 mM), and KCl (100 mM). Potassium chloride was included in the buffer system, as the presence of monovalent cations has previously been shown to enhance the rate of ATP-grasp-catalyzed reactions.^18,52^ Aliquots of the reaction mixture were removed and quenched with formic acid (1% v/v) before LC-MS analysis. Gratifyingly, after 3 h, the major product was doubly dehydrated ThfA1 with a conversion of ∼51% (Figure S17). The same reaction conditions lead to ∼81% conversion of ThfA to the doubly dehydrated product (Figure S18), revealing that repositioning the leader sequence to the C-terminus reduces the rate of ThfB-mediated modification. The initial rate of reaction was determined by plotting the natural log of precursor peptide concentration (determined by ion count from LC-MS analysis) against time. The observed rate of precursor peptide depletion was 0.029 ± 0.002 min^–1^ and 0.017 ± 0.002 min^–1^ for ThfA and ThfA1, respectively. These results support the *in cellulo* experiments, demonstrating that a precursor peptide can still be recognized and modified by the tailoring enzyme, despite the leader sequence being repositioned to the C-terminus. The engineered precursor ThfA1 was, however, modified at a lower rate than the native substrate ThfA (with the typical N-terminal leader sequence), but still demonstrated catalytic activity of ThfB, which was present at a substoichiometric ratio (0.1 equiv). Minimal accumulation of the singly dehydrated product was observed for ThfA1 with the unnatural C-terminal leader sequence, showing that the second ester cross-link is likely installed more rapidly than the first, equivalent to that seen for the natural precursor with the N-terminal leader.^18^

Varying the C-terminal leader sequence allowed the minimum required sequence to be determined for ThfB-mediated modification of the precursor peptide. Addition of an arbitrary sequence (SUMO) to the C-terminus instead of the leader sequence in ThfA7 was employed to test whether the leader sequence was required at all. When coexpressed with ThfB, no dehydration of ThfA7 was observed (Figure S19), demonstrating that SUMO was ineffective at recruiting ThfB for peptide modification. Interestingly, purification under native conditions pulled down some ThfB, showing that ThfB interacts with the core sequence but with a diminished affinity compared to that of the leader sequence. Despite the interaction of the core sequence with ThfB, no modification of the precursor was observed, confirming that ThfB is truly a leader-dependent tailoring enzyme. Constructs ThfA8–12 with truncations of the leader sequence from the C-terminus were each coexpressed with ThfB and evaluated by LC-MS analysis (Figures S20–S23). As more of the leader sequence was removed from the precursor peptide, the modification efficiency decreased (Figure 2), in line with studies that truncated the N-terminal leader from the N-terminus.^18^ Truncation of 50 amino acids from the C-terminus in construct ThfA12 resulted in no detectable product being isolated, presumably because the peptide was too short and was therefore proteolyzed within the cell. Remarkably, 40 aa could be removed from the C-terminus of the 67 aa leader sequence and still lead to full 2-fold modification of the precursor (Figure 2, mThf11^B^). In contrast, a maximum of 20 aa can be removed from the N-terminus before modification of the precursor is lost entirely.^18^ This strategy serves as an alternative method to an Ala scan for the identification of the recognition sequence within the leader sequence, which is essential for the recruitment of leader-dependent tailoring enzymes. The RPFG tetrapeptide sequence (Figure 2, yellow residues) has previously been identified as a highly conserved motif through bioinformatic analysis of aligned precursor sequences of several putative graspimiditides.^18^ This conserved sequence lies within the bounds of the limits of tolerable C- and N-terminal truncations, suggesting that this is a key recognition sequence within ThfA.

**Figure 2:**
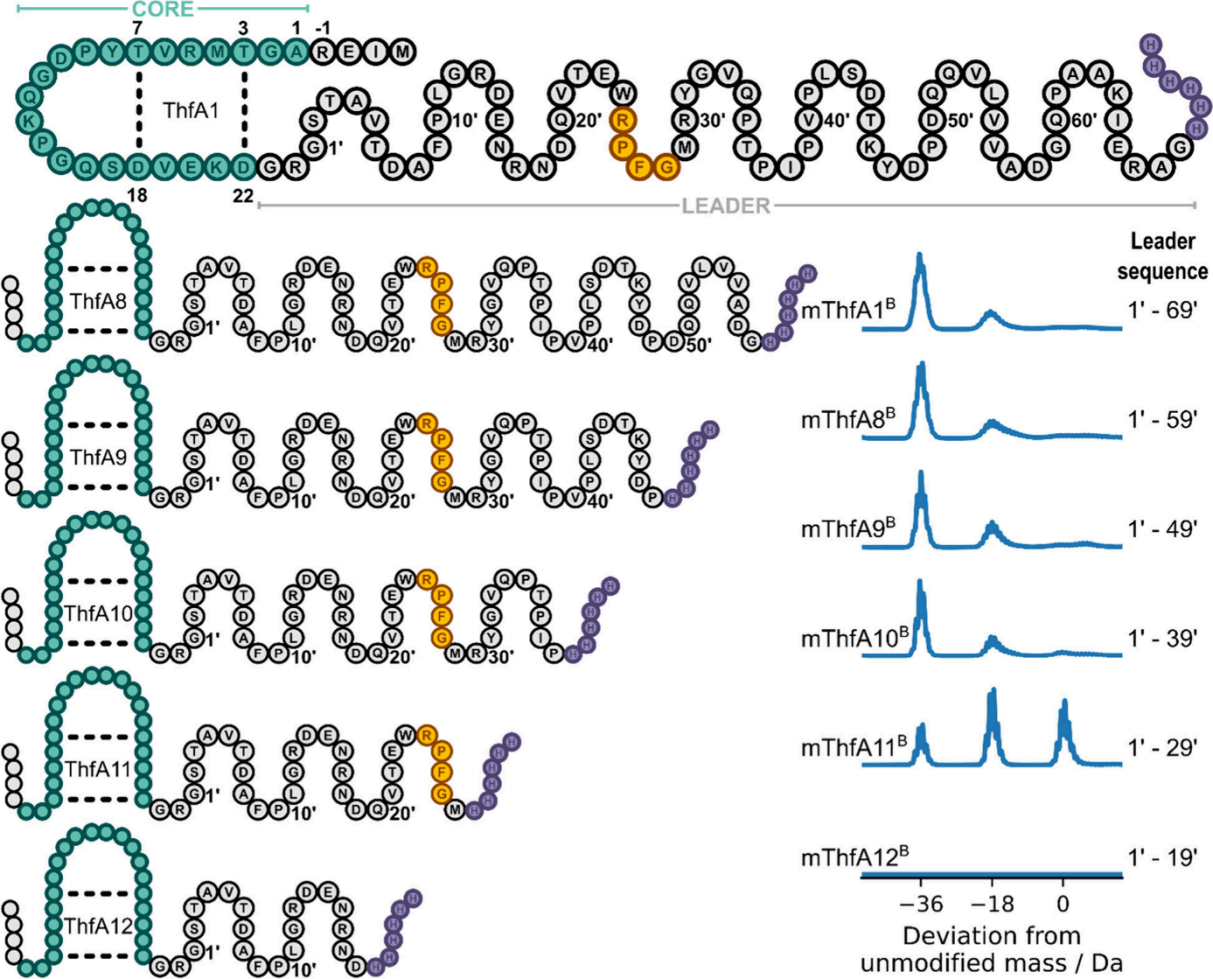
Determining how much of the C-terminal leader sequence is dispensable. The full sequence of ThfA1 is shown, with the N-terminal core (green), C-terminal leader (gray), putative tetrapeptide recognition sequence (yellow), and His_6_ tag (purple). ThfA8–12 are shown with consecutive 10 aa truncations from the C-terminal end of the leader sequence per construct to evaluate the effect on ThfB-mediated modification. Deconvoluted mass spectra of ThfB-modified constructs ThfA8–12 with varying truncations from the C-terminal leader sequence were obtained and compared to mThfA1^B^.

To evaluate the applicability of the leader relocation strategy, another member of the graspimiditide RiPP family was investigated. Amycolimiditide is a 29 aa graspimiditide with a stem-loop structure formed from four ester cross-links containing an aspartimide in the loop,^53^ similar to the structure of fuscimiditide. Heterologous expression of the N-terminally His_6_ tagged native precursor peptide (AmdA) and ATP-grasp enzyme (AmdB) in *E. coli* yielded the 4-fold dehydrated product mAmdA^B^, corresponding to the enzymatic installation of four ester cross-links by AmdB (Figure S24). Coexpression of an engineered amycolimiditide precursor (AmdA1), with the leader sequence repositioned to the C-terminus, with AmdB *in cellulo* resulted in up to 2-fold dehydration of the precursor peptide (Figure S25). In the native biosynthesis of amycolimiditide the ester cross-links were found to be installed in a strict order starting at the loop and proceeding down the stem.^53^ Without the innermost cross-link, none of the subsequent cross-links were installed. Therefore, the observation of products with more than one dehydration suggests that cross-links are installed in the native positions. The reduced modification efficiency of engineered amycolimiditide constructs compared to fuscimiditide constructs is typical of our experience and highlights the promiscuous nature of the modifying enzyme ThfB, demonstrating the ThfA/ThfB system to be a privileged platform for bioengineering.^50,51^

### Engineering Fuscimiditide Constructs with Multiple Leader Sequences

Fuscimiditide constructs with two and three core peptide repeats were designed to mimic the multivalent structures of other graspetides, such as plesiocin,^54^ chryseoviridin,^55^ and thuringinin.^56^ We have recently shown that the ThfA precursor can be engineered to contain tandem repeats of the core peptide appended to the C-terminus and still be modified by ThfB to create multivalent structures.^50^ Constructs ThfA13–15 were designed with the leader sequence repositioned to the C-terminus and coexpressed with ThfB to assess the ability of the rearranged leader sequence to recruit the tailoring enzyme and promote modification of these multivalent systems (Figure 3). The product distributions for mThfA13^B^ and mThfA14^B^ were similar to those observed for the constructs with the leader sequence in the native N-terminal position (Figures S26, S27),^50^ revealing the effectiveness of the repositioned leader sequence to mediate modification of engineered ThfA constructs. Equivalent to the N-terminal leader constructs, the C-terminal leader constructs displayed a reduced proportion of the product with the maximum number of modifications as the number of potential cross-links increased in the precursor. To improve the modification efficiency of the trivalent core, we designed ThfA15 with both N-terminal and C-terminal leader sequence. Remarkably, coexpression of this trivalent core peptide with ThfB gave a higher proportion of the maximally 6-fold dehydrated product (Figure 3, Figure S28). The incorporation of a second leader sequence enhanced the maturation of the fully modified multivalent product, aiding in the design of more complex engineered new-to-nature peptide constructs.

**Figure 3:**
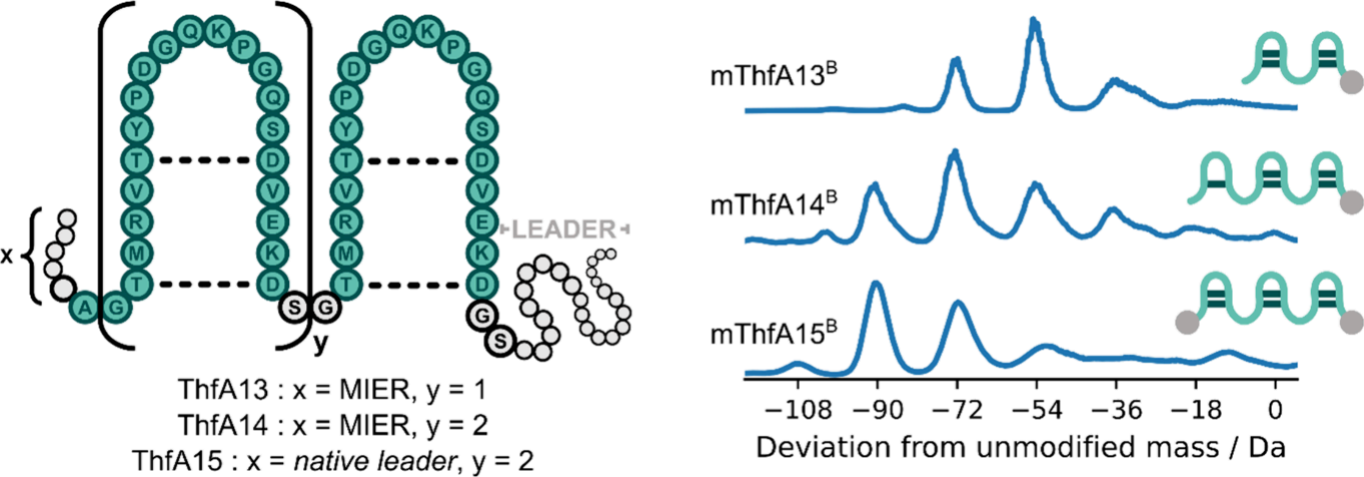
Forming multivalent peptide constructs from ThfA with a C-terminal leader sequence. General structure of the multivalent constructs with the truncated (MIER) or full native N-terminal leader sequence (gray circles) followed by the core peptide repeats (green) connected by SG linkers and followed by a C-terminal leader sequence (gray circles), with expected cross-links depicted as dashed lines. Deconvoluted mass spectra of ThfB-modified ThfA13–15, where a −72 Da peak shift for mThfA13^B^ from the unmodified mass indicates formation of the desired four ester cross-links and a −108 Da peak shift for mThfA14^B^ and mThfA15^B^ from the unmodified mass indicates formation of the desired six ester cross-links. Schematic of the maximally dehydrated product observed showing the proposed chemical connectivity and leader sequence (gray circle) are included next to the corresponding deconvoluted mass spectra.

### Generating Chimeric RiPPs through Incorporation of Repositioned Leader Sequences

Having demonstrated that two leader sequences could be incorporated into an engineered RiPP construct, we were inspired to combine the precursors of two different RiPP natural products into a single chimeric peptide fusion. Through combining the graspimiditide biosynthetic pathway with that of a lasso peptide, we strived to create a highly post-translationally modified interlocked peptide structure. Lasso peptides typically only allow minor substitutions within the core sequence, but examples of fusion proteins have been reported for astexin-1,^57^ pandonodin,^58^ and other lasso peptides,^59,60^ in which the interlocked structure was efficiently matured with a C-terminal extension of the lasso peptide tail. Mitchell et al. have reported a method to enzymatically modify the C-terminus of the lasso peptide fusilassin (also known as fuscanodin),^61^ but this was only achieved *in vitro*.^62^ We chose to form chimeric peptide fusions with pandonodin, as pandonodin fusion proteins are converted to the interlocked architecture more efficiently than astexin-1 constructs. Precursor ThfA16 was designed with the native pandonodin precursor (PanA) followed by the ThfA core sequence, a GS linker, and finally the ThfA leader sequence with a C-terminal His_6_ tag (Figure 4). A chimeric biosynthetic gene cluster was designed across different vectors to enable the combinatorial coexpression of the precursor peptide with different tailoring enzymes to evaluate PTM (Figure 4A). Coexpression of ThfA16 with the lasso peptide biosynthetic enzymes (PanB, PanC, and PanD) gave the desired truncated product with one dehydration, corresponding to excision of the lasso leader sequence by PanB and installation of the lasso isopeptide bond by PanC (Figure 4Bi). The full-length precursor peptide ThfA16 with no dehydrations and truncated singularly dehydrated peptide mThfA16^PanBC^ were isolated from the cell pellet, equivalent to previously reported pandonodin–protein fusions,^58^ demonstrating that the ABC-transporter PanD was ineffective at exporting these proteins from the cell. No truncated product mThfA16^PanBC^ without dehydration was observed (Figure S29), revealing that formation of the lasso isopeptide bond by PanC occurs rapidly and efficiently after cleavage of the pandonodin leader sequence, despite the 98 aa C-terminal extension to the peptide sequence.

**Figure 4:**
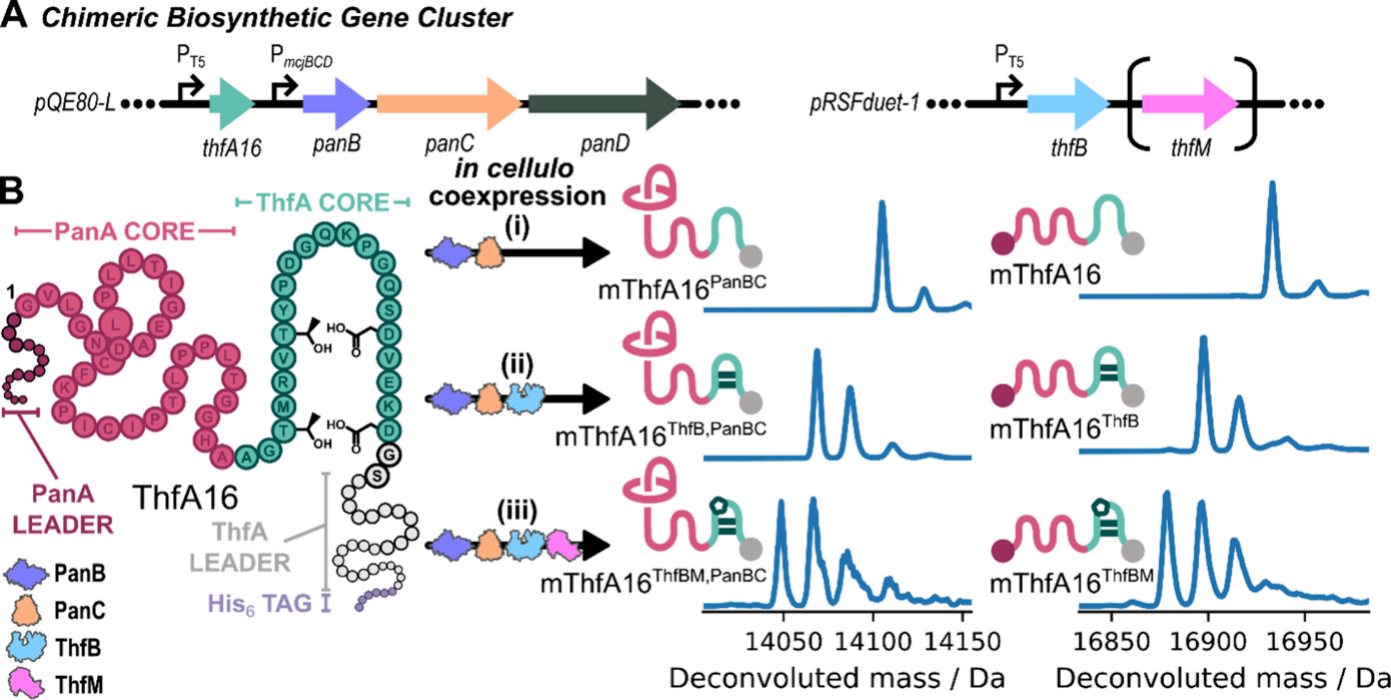
Combining precursors from two different RiPP natural product classes into a single chimeric peptide fusion enables generation of a highly post-translationally modified peptide *in cellulo*. A: Gene organization for the heterologous expression of the chimeric lasso-graspimiditide peptide fusion shows the two plasmids used to combinatorially test tailoring enzymes. B: The ribosomally synthesized linear precursor protein (ThfA16) is composed of an N-terminal PanA leader sequence (dark pink), PanA core (pink), ThfA core (light green), a GS linker, and ThfA leader sequence (gray) followed by a His_6_ tag (lavender) at the C-terminus. (i) Deconvoluted mass spectra of ThfA16 coexpressed with PanBCD showed a product with the PanA leader excised and a −18 Da peak shift corresponding to formation of an isopeptide bond, as expected for the lasso peptide fusion (left), as well as the full-length precursor with no dehydrations (right). (ii) Deconvoluted mass spectra of ThfA16 coexpressed with PanBCD and ThfB showed up to 3-fold dehydration of the PanA leader excised product corresponding to formation of an isopeptide bond and two ester cross-links (left), while the full-length precursor was observed with up to 2-fold dehydration corresponding to formation of two ester cross-links (right). (iii) Deconvoluted mass spectra of ThfA16 coexpressed with PanBCD and ThfBM showed up to 4-fold dehydration of the PanA leader excised product corresponding to formation of an isopeptide bond, two ester cross-links, and an aspartimide (left), while the full-length precursor was observed with up to 3-fold dehydration corresponding to formation of two ester cross-links and an aspartimide (right). Schematics of the major product showing the proposed chemical connectivity and leader sequences (depicted as dark pink and gray circles) are included next to the corresponding deconvoluted mass spectra.

Coexpression of ThfA16 was then investigated with the lasso biosynthetic enzymes and transporter (PanB, PanC, and PanD), as well as with the graspimiditide biosynthetic enzymes (ThfB alone or ThfB and ThfM). Gratifyingly, when ThfA16 was coexpressed with PanBCD and ThfB, the truncated product with two and three dehydrations was observed, consistent with a lasso peptide containing one or two ester cross-links in the tail (Figure 4Bii, Figure S30). The linear precursor was also observed with one and two dehydrations, with two dehydrations being the major product. The ratio of the lasso product to the full-length peptide in the presence and absence of the tailoring enzyme ThfB was found to be roughly 1:1. The minimal change in the modification efficiency of the lasso peptide tailoring enzymes demonstrates that ThfB-mediated PTM does not impact the maturation of the lasso structure. This supports the expectation that cross-links are installed within the ThfA portion of the precursor separately from PanA. Coexpression of ThfA16 with all the tailoring enzymes (PanB, PanC, ThfB, ThfM) gave the expected truncated product with up to four dehydrations, consistent with a set of 5 PTMs: excision of the PanA lasso leader sequence, installation of the lasso isopeptide bond, two graspetide ω-ester cross-links, and an aspartimidylation (Figure 4Biii, Figure S31). Variant ThfA17 with a GRG-linker between the ThfA core and the ThfA leader sequence was designed to include a trypsin cleavage site to allow *in vitro* digestion of the modified product and MS analysis. Coexpression of ThfA17 with different combinations of the lasso peptide and graspetide biosynthetic enzymes gave product distributions equivalent to those of ThfA16 (Figures S32–S34). Analysis of the trypsin digestion products of ThfA17 coexpressed with PanBCD and ThfB confirmed that ester cross-links installed by ThfB were generated within the ThfA portion of the molecule (Figure S35), supporting the proposed structure where two unique classes of RiPPs are fused directly next to one another.

Modification of the two RiPP core peptides in ThfA16 and ThfA17 appears to occur independently of one another, to generate a chimeric peptide that comprises two distinct structures consecutively linked together. However, we were interested in creating products that merged structural features from both classes of RiPPs into synergistic products, to generate densely functionalized chimeric RiPP products (Figure 5A). A range of peptide sequences were designed (ThfA18–23) with different tail truncations of the lasso precursor sequence followed by DVEKD (the native C-terminal stem sequence of fuscimiditide), a GRG-linker, the ThfA leader sequence, and finally a C-terminal His_6_ tag (Figure 5B). The full PanA leader sequence was retained in these constructs to maintain modification by the lasso tailoring enzymes (PanBCD), but tail truncations of the PanA core sequence were included to vary the separation between the nucleophilic residues within PanA and the aspartic acid residues within the DVEKD sequence C-terminal to the lasso peptide. It was expected that ThfB would still be recruited to the precursor and enzymatically phosphorylate the aspartic acid residues to generate activated esters for nucleophilic attack by residues from the pandonodin core sequence. The distance between the Asp residue and nucleophilic partner residue (Thr, Ser, Cys, Lys) has previously been shown to be integral to the observation of a cross-linked product.^50,51^

**Figure 5:**
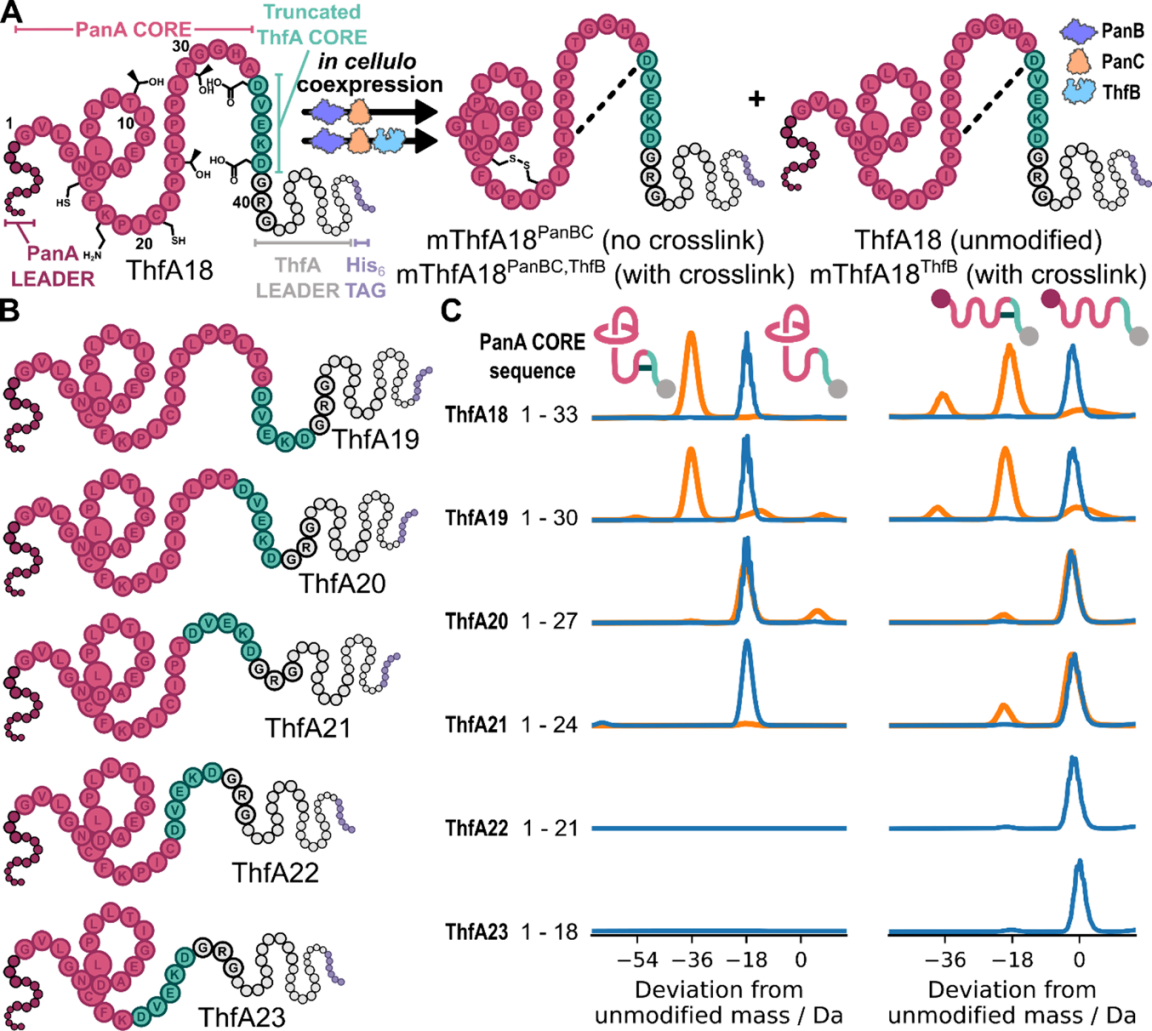
Preparing lasso peptides with an enzymatically cross-linked tail macrocycle. A: Schematic biosynthetic pathway of the lasso peptide fusion. The ribosomally synthesized linear precursor peptide (ThfA18) is composed of an N-terminal PanA leader sequence (dark pink), PanA core (pink), DVEKD sequence from ThfA (green), GRG linker, and C-terminal ThfA leader sequence (gray). B: Precursor peptides ThfA19–23 with the core peptide aa labeled. C: Left, deconvoluted mass spectra of ThfA18–23 coexpressed with PanBC (blue data) showing the lasso peptide with the PanA leader excised and a −18 Da peak shift corresponding to formation of an isopeptide bond. Deconvoluted mass spectra of ThfA18–21 coexpressed with PanBC and ThfB (orange data) overlaid, where −18 and −36 Da peak shifts correspond to no and 1 cross-link, respectively, installed by ThfB in the tail (orange data). Right, deconvoluted mass spectra of ThfA18–23 coexpressed with PanBC (blue data) showing the linear precursor, overlaid with data for ThfA18–21 coexpressed with PanBC and ThfB (orange data) where −18 and −36 Da peak shifts correspond to 1 and 2 cross-links, respectively, installed by ThfB. Schematics of the major product showing the proposed chemical connectivity and leader sequences (depicted as circles) are included next to the corresponding deconvoluted mass spectra.

The ability of the variants (ThfA18–23) to form a mechanically interlocked lasso product was first investigated through coexpression with PanBCD in *E. coli* (Figure 5C, blue lines). Variant ThfA18, with the full-length PanA sequence, was effectively converted into the lasso product (Figure 5C, Figure S36) with a conversion efficiency equivalent to variants ThfA16 and ThfA17, which also contain the full PanA sequence. Three, six, and nine aa truncations shortening the C-terminal tail of the PanA core sequence (ThfA19, ThfA20, and ThfA21, respectively) did not alter the ability of the precursor to be matured into the lasso product, with yields of lasso-protein fusion being equivalent to the full-length precursor (Figure 5C, Figures S37–S39). However, upon removal of 12 or more amino acids from the tail (ThfA22–23), no matured lasso products were detected by LCMS (Figure 5C, Figures S40 and S41). To probe the ability of ThfB to form cross-links with the lasso peptide core sequence, variants ThfA18–21 were then coexpressed with PanBCD and ThfB (Figure 5C, orange lines). Comparing the product distribution of dehydrated products of the full-length precursor allows the modification by ThfB to be uncoupled from PanBC. Onefold dehydration of the full-length precursor was the major product for mThfA18^ThfB^ and mThfA19^ThfB^ (Figure 5C, Figures S42 and S43), demonstrating that this method of appending just DVEKD and a repositioned ThfA leader sequence to a peptide can generate ThfB-modified products. However, for ThfA20 and ThfA21, ThfB modification of the full-length peptide was notably reduced, with the unmodified product being the major species observed (Figure 5C, Figures S44 and S45). Interestingly, when ThfA18 and ThfA19 were each coexpressed with PanBCD and ThfB, the PanA leader excised product was 2-fold dehydrated, corresponding to a ThfB-modified lasso product (Figure 5, Figures S42 and S43). Since ThfB modification did not impede the ability of PanBC to mature a lasso product, the cross-link is likely introduced within the tail portion of PanA, after steric lock residue Cys-16. Observation of disulfide-bonded products in mThfA18^PanBC,ThfB^ and mThfA19^PanBC,ThfB^ (Figures S42, S43) confirmed that the cysteines were not involved in the cross-link. Therefore, residues K18, T24, and T29 are the potential nucleophiles involved in forming the ThfB-installed cross-link. ThfA24–25 were engineered as variants of ThfA18 which substitute the ThfB-targeted electrophilic residues for unreactive Asn (Asp34Asn and Asp34Asn/Asp38Asn, respectively). When coexpressed with PanBCD and ThfB, lasso peptide formation occurs in both ThfA24 and ThfA25 but ThfB modification occurs only minimally or not at all for ThfA24 and ThfA25, respectively (Figures S46, S47). Therefore, in mThfA18^PanBC,ThfB^ the cross-link is likely between Thr-24 and Asp-34, as this would generate an 11 aa macrocycle which most closely matches the native Thr-Asp cross-link in the 12 aa macrocycle of fuscimiditide. The equivalent cross-link in mThfA19^PanBC,ThfB^ (Thr-24 to Asp-31) would result in an 8 aa macrocycle. Loss of ThfB-modified lasso products in constructs ThfA20 and ThfA21 also supports this connectivity, as the equivalent cross-link would generate 5 and 2 aa macrocycles, which we have previously shown are too sterically demanding to form.^50^ The distribution of dehydrated products was highly dependent on the number of intervening aa between the bottom steric lock residue (PanA Cys-16) and the first Asp of the ThfA portion of the hybrid precursor, generating mechanically interlocked peptides with a new-to-nature enzymatically cross-linked tail macrocycle.

## Conclusion

Here we have shown that repositioning the leader sequence of the RiPP precursor ThfA from the N-terminus to the C-terminus leads to the same post-translational modifications of the core sequence by the native tailoring enzymes. Reconstitution of the biosynthesis *in vitro* revealed that the rearranged precursor with the C-terminal appended leader was modified at half the rate of the natively arranged precursor ThfA, with an N-terminal leader. The long leader sequence of ThfA is comparable to that of cyanobactins,^38^ thiazole/oxazole-modified microcins,^63^ and proteusins,^64^ which have been shown to fold into defined three-dimensional structures to facilitate protein/protein engagement of the tailoring enzymes.^65^ Demonstration of this “leader to follower” design in another graspimiditide, amycolimiditide, suggests that this engineering strategy may be applicable to other families of RiPPs with long leader sequences that may be especially amenable to conversion into a follower. C-Terminal truncations of the rearranged precursor revealed that ThfB was able to effectively modify the precursor with up to 40 amino acids removed, showing that much of the C-terminal portion of the leader sequence was dispensable. This enabled the position of a key recognition sequence within the leader peptide to be identified as the RPFG tetrapeptide, which was previously seen to be highly conserved among graspimiditide precursors.^18^ These results, coupled with the observation that ThfA is a random coil,^18^ suggest a model in which only a short segment of the leader peptide engages with the enzyme and most of the leader peptide is unstructured, serving as a linker that allows for either N- or C-terminal placement of the core peptide in the precursor.

Having established that a C-terminally appended leader sequence can still recruit the tailoring enzymes to modify the native core sequence, we were interested in investigating the ability of a C-terminal leader sequence to modify engineered precursor peptides. Multivalent constructs with a single C-terminal leader sequence demonstrated that multiple core sequences could be modified to produce divalent and trivalent cores with up to 6 cross-links. Whether the leader sequence was in the native N-terminal position or the rearranged C-terminal position did not affect the modification efficiency; however, this strategy enabled a construct with two leader sequences to be designed which displayed an improved proportion of the maximally 6-fold cross-linked product compared to constructs with a single leader. Given that the introduction of two leader sequences into a precursor peptide improved the modification efficiency of a single tailoring enzyme, we were inspired to design precursors with two different leader sequences to recruit multiple sets of tailoring enzymes.

Post-translation modifications of lasso peptides typically occur on the linear precursor before excision of the leader peptide and formation of the isopeptide bond, which locks the peptide into the [1]­rotaxane architecture. A rare exception to this biosynthetic order is aspartimidylation, which has been shown to occur exclusively on the interlocked lasso peptides cellulonodin-2 and lihuanodin rather than their linear precursors.^66^ In the chimeric PanA-ThfA peptide fusions, we have shown that this same biosynthetic ordering of post-translational modification is followed. ThfB-mediated cyclization occurs rapidly, despite the rate being reduced due to the C-terminal rearranged leader, with cross-linking of the linear precursors being observed, prior to excision of the N-terminal lasso leader sequence and maturation into the interlocked architecture. The ThfA16 construct (Figure 4) demonstrates that a precursor peptide can be modified by all four tailoring enzymes from both biosynthetic pathways to give a highly functionalized [1]­rotaxane peptide containing a total of five post-translational modifications. This work demonstrates a new approach to access hybrid RiPPs, beyond engineering the tailoring of enzymes with the leader sequence and generating chimeric N-terminal leader sequences for a precursor peptide. Our strategy has the benefit of being able to be carried out either *in cellulo* or *in vitro*, expanding the scope of designer peptides to include mechanically interlocked architectures for a variety of applications.

## Supplementary Material



## Notes

Detailed materials and methods, additional mass spectrometry data, and *in vitro* reaction data (PDF)
