# Supplementary material for: Repositioning the Leader Peptide in Graspetide Biosynthesis
Source: J Am Chem Soc. 2026 Mar 6;148(10):10859–68. doi: 10.1021/jacs.5c21135 (PMC13003496; doi:10.1021/jacs.5c21135)
Supplement: Supplementary file 1 [file ja5c21135_si_001.pdf]

Supporting Information

Repositioning the Leader Peptide in Graspetide Biosynthesis

Toby G. Johnson,[†,£] Dean M. Miller,[†] Drew V. Carson,[†] Brian Choi,[†] and A. James Link[†,‡,§,£] *

[†]Department of Chemical and Biological Engineering, Princeton University, Princeton, NJ 08544, United States
[£]Omenn-Darling Bioengineering Institute, Princeton University, Princeton, NJ 08544, United States
[‡]Department of Chemistry, Princeton University, Princeton, NJ 08544, United States
[§]Department of Molecular Biology, Princeton University, Princeton, NJ 08544, United States
*Corresponding Author: ajlink@princeton.edu

Table of Contents

Methods ... 2
General ... 2
Cloning ... 4
Protein Expression ... 4
Liquid Chromatography–Mass Spectrometry (LC-MS) ... 5
Trypsin Digestion ... 6
High-Pressure Liquid Chromatography (HPLC) ... 6
Ester-Selective Hydrazinolysis ... 6
In vitro Enzyme Reconstitution ... 6
Supplementary Figures ... 20
References for Supplemental Information ... 58

Methods

General

Enzymes, reagents and consumables: Q5 High Fidelity DNA polymerase, Q5 polymerase buffer, dNTPs, restriction enzymes used for molecular cloning, T4 DNA Ligase and T4 ligase buffer were purchased from New England Biolabs (NEB). Trypsin was purchased from Promega. Yeast extract and tryptone were purchased from IBI Scientific. Ampicillin and kanamycin were purchased from Neta Scientific, Inc. Lysozyme (Cat. No. L6876), ATP (Cat. No. A2383) and Amicon® Ultra Centrifugal Filters (10 kDa MWCO, 4 mL, UFC8010) were purchased from Sigma-Aldrich. Ni-NTA resin (Cat. No. 88222) was purchased from Thermo Fisher Scientific.

Synthetic DNA: Synthetic DNA primers were obtained from Integrated DNA Technologies (IDT) and GENEWIZ (from Azenta Life Sciences).

Plasmid and DNA Purification: Amplified inserts were purified using Zymoclean™ Gel DNA Recovery Kit (Zymo Research) after gel electrophoresis following the manufacturer's protocol. Plasmids were purified using QIAprep Spin Miniprep Kit (QIAGEN) according to the manufacturer's protocol.

Gel Electrophoresis: UltraPure™ Agarose (Invitrogen) was used for gel analysis dissolved in TAE buffer and mixed with ethidium bromide to allow DNA visualization. DNA samples mixed with DNA Loading Dye (Cat. No. B7025S) were run alongside the necessary DNA ladder (1 kb DNA Ladder (N3232S) and 100 bp DNA Ladder (N3231S) were purchased from NEB).

Competent Cells: Chemically competent *E. coli* XL1-Blue (Stratagene) cells used for plasmid maintenance were prepared with Mix & Go! Transformation Kit (Zymo Research), while *E. coli* BL21 (DE3) Δ*slyD* cells were employed for protein production and purification. Both competent cell types were prepared in-house and used for transformation following established protocols.

Plasmid Sequence Verification: DNA sequences of constructed plasmids were confirmed by Sanger Sequencing (GENEWIZ/Azenta Life Sciences) or whole plasmid sequencing (Plasmidsaurus).

LC-MS(/MS) Analysis: Performed with an Agilent 6530 QTOF connected to an Agilent 1260 Infinity II LC system. Mass spectra were acquired using electrospray ionization (ESI) with the instrument in positive ion mode. Data from mass spectrometry was processed using Qualitative Analysis and Bioconfirm Agilent MassHunter software.

Composition of media and buffers used in the study can be found in Table S1.

NCBI GenBank accession numbers and native hosts of proteins used in this study can be found in Table S2.

Plasmids used in this study with the sequence of the protein expressed and cloning methodology can be found in Table S3.

The sequence of oligonucleotide primers used in this study for cloning can be found in Table S4.

Table S1. List of media and buffers used in this study.

Name	Composition
LB Broth	Tryptone (10.0 g/L), yeast extract (5.0 g/L), NaCl (10.0 g/L)
LB Agar	Tryptone (10.0 g/L), yeast extract (5.0 g/L), NaCl (10.0 g/L), agar (15.0 g/L)
TAE Buffer	Tris (40 mM), acetic acid (20 mM), EDTA (1 mM)
Lysis Buffer	NaH_2PO_4 (50 mM), NaCl (300 mM), Imidazole (10 mM), pH 8.0
Native Wash Buffer 1	NaH_2PO_4 (50 mM), NaCl (300 mM), Imidazole (20 mM), pH 8.0
Native Wash Buffer 2	NaH_2PO_4 (50 mM), NaCl (300 mM), Imidazole (50 mM), pH 8.0
Native Elution Buffer	NaH_2PO_4 (50 mM), NaCl (300 mM), Imidazole (250 mM), pH 8.0
PBS Buffer*	NaH_2PO_4 (10 mM), KH_2PO_4 (1.8 mM), NaCl (137 mM), KCl (2.7 mM), pH 7.4
Protein Storage Buffer	1X PBS, Glycerol (10% w/v)
Urea Buffer B	Urea (8M), NaH_2PO_4 (100 mM), Tris (10 mM), pH 8.0
Urea Buffer C	Urea (8M), NaH_2PO_4 (100 mM), Tris (10 mM), pH 6.4
Urea Buffer D	Urea (8M), NaH_2PO_4 (100 mM), Tris (10 mM), pH 5.9
Urea Buffer E	Urea (8M), NaH_2PO_4 (100 mM), Tris (10 mM), pH 4.5
Hydrazinolysis solution	1X PBS, hydrazine (10 wt. % in H_2O), pH 8 (adjusted with 6M HCl)
ThfB *in vitro* reaction buffer	NaH_2PO_4 (100 mM), KCl (100 mM), $MgCl_2$ (10 mM), ATP (10 mM), DTT (10 mM), pH 6.9

*prepared using the Cold Spring Harbor Protocol

Table S2. Native hosts and NCBI GenBank accession numbers of the proteins used in this study.

Protein	Native Host	Protein Accession Number	Nucleotide Accession Number
ThfA	*Thermobifida fusca* (DSM 43792 / NBRC 14071)	WP_011292231.1	NZ_BCWB01000024.1 36393-36662 (+)
ThfB	*Thermobifida fusca* (DSM 43792 / NBRC 14071)	WP_011292230.1	NZ_BCWB01000024.1 35443-36393 (+)
ThfM	*Thermobifida fusca* (DSM 43792 / NBRC 14071)	WP_011292232.1	NZ_BCWB01000024.1 36668-37798 (+)
PanA	*Pandoraea norimbergensis* (DSM 11628)	WP_058377877.1	NZ_CP013480.3 1649034-1649216 (+)
PanB	*Pandoraea norimbergensis* (DSM 11628)	WP_058377878.1	NZ_CP013480.3 1649209-1649970 (+)
PanC	*Pandoraea norimbergensis* (DSM 11628)	WP_058377879.1	NZ_CP013480.3 1650044-1651801 (+)
PanD	*Pandoraea norimbergensis* (DSM 11628)	WP_084656080.1	NZ_CP013480.3 1651798-1653645 (+)

Cloning

For molecular cloning, Golden Gate assembly was used as the primary method to construct ThfA variant plasmids using a Golden Gate assembly-enabled pQE-80L-based vector (pBC108) that we previously reported.[1] This vector was generated from pQE-80L, in which the *Bsa*I recognition site in AmpR was ablated with a silence mutation (G239G, GGG → GGT) and the multicloning site (MCS) of pQE-80L was replaced with a GFP constitutive expression cassette flanked by *Bsa*I recognition sites that generate *Bam*HI and *Hind*III sticky ends upon digestion. The GFP constitutive expression cassette contains the coding sequence of sfGFP (avGFP S2R S30R Y39N F64L S65T S72A F99S N105T Y145F M153T V163A I171V A206V) under the control of p_{glpT} promoter (Part: jtk2821; BBa_J72163 from iGEM Registry of Standard Biological Parts). The arginine residue of the His_6 tag was also mutated to serine to suppress the background methylation of the N-terminus in *E. coli.*

Inserts were prepared by PCR amplification of template DNA using the necessary primers as described in Table S3, with details of the primer sequences listed in Table S4. Golden Gate assembly of purified amplified insert(s) and plasmid (pBC108) or pre-digested plasmid was carried out with *Bsa*I-HFv2 and T4 DNA ligase in T4 ligase buffer with the following thermal cycling procedure. Incubation at 37 °C (1 min) followed by 16 °C (30 sec) was cycled 25 times in total, before heating to 85 °C (5 min) and finally samples were kept at 12 °C until used for transformation. To propagate the assembled plasmids, chemically competent *E. coli* XL1-Blue cells were transformed with the ligation mixture and grown in lysogeny broth (LB) media supplemented with ampicillin (100 mg L^{-1}), as needed for selection. Plasmids were recovered, purified and sequenced to confirm the identity of the inserted fragments.

Protein Expression

Typically, a plasmid encoding a ThfA variant and a plasmid encoding ThfB were transformed into electrocompetent *E. coli* BL21 (DE3) Δ*slyD* cells and plated onto LB agar containing ampicillin (100 mg L^{-1}) and kanamycin (50 mg L^{-1}). Starter cultures were created by picking colonies into LB (5 mL, 100 mg L^{-1} ampicillin, 50 mg L^{-1} kanamycin) and grown overnight at 37 °C. These cells were subcultured into LB media (500 mL, 100 mg L^{-1} ampicillin, 50 mg L^{-1} kanamycin) at a starting optical density measured at 600 nm (OD_{600}) of 0.02 and grown at 37 °C. Upon reaching an OD_{600} of 0.5, cells were induced with 1 M IPTG (500 μL, final concentration 1 mM) and left to shake at rt (24 h). Cells were then pelleted by centrifugation (4000 *g*, 15 min, 4 °C) before proceeding with protein purification.

Denaturing Protein Purification

For denaturing purification, the cell pellet (per 500 mL of culture) was resuspended in urea buffer B (10 mL, pH 8.0) and frozen (-80 °C). The frozen cell suspension was thawed (water bath, rt) before centrifugation (15,000 *g*, 1 h, 4 °C) to isolate a clarified cell lysate. Ni-NTA resin (1 mL resin) was added to the lysate and left to incubate (1 h, 4 °C). This suspension was then

added to a gravity column, washed with urea buffer C (10 mL, pH 6.4) and then urea buffer D (10 mL, pH 5.9). Protein was then eluted with urea buffer E (pH 4.5), collecting 8×1 mL fractions.

Native Protein Purification

For native purification, the cell pellet (per 500 mL of culture) was resuspended in lysis buffer (10 mL). Lysozyme (10 mg) was added to the cell suspension and left to incubate (20 min, 4 °C). The cell suspension was then sonicated (12 cycles of 10 s on/20 s off, 0 °C) to lyse the cells before centrifugation (10,000 *g*, 30 min, 4 °C) to isolate a clarified cell lysate. Ni-NTA resin (1 mL resin) was added to the lysate and left to incubate (1 h, 4 °C). In a cold room (4 °C), this suspension was then added to a gravity column, washed with native wash buffer 1 (10 mL) and then native wash buffer 2 (20 mL). Protein was then eluted with native elution buffer, collecting 8×1 mL fractions. Fractions 2-5 were pooled, concentrated (centrifugal filter, 10 kDa MWCO, 4 mL) and buffer exchanged into the protein storage buffer before being frozen (- 80 °C) for storage.

Liquid Chromatography–Mass Spectrometry (LC-MS)

LC-MS analysis was performed using an Agilent 6530 QTOF connected to an Agilent 1260 Infinity II LC system. Mass spectra were acquired using electrospray ionization (ESI) with the instrument in positive ion mode. The mobile phase A was water with 0.1% formic acid, and mobile phase B was acetonitrile with 0.1% formic acid.

Proteins were run on a Xbridge Protein BEH C4 column (2.1 mm × 50 mm, 3.5 μM particle size, Waters) with a flow rate of 0.5 mL min^{-1} using the following method: 10% B, 0-2 min; 10-50% B, 2-15 min; 50-90% B, 15-20 min; 90% B, 20-30 min. Deconvoluted mass spectra of the analyte (ThfA variant) were acquired using the Agilent MassHunter Bioconfirm software.

Trypsin digestion products and HPLC-purified core peptides (see below) were run on a Zorbax 300SB-C18 column (2.1 mm x 50 mm, 3.5 μm particle size) with a flow rate of 0.5 mL min^{-1} using the following method: 5% B, 0-1 min; 5-45% B, 1-20 min; 45-90% B, 20-25 min; 90% B, 25-30 min. The resulting LC traces and mass spectra were extracted and analyzed using the Agilent MassHunter Qualitative Analysis software for manual inspection. The Agilent MassHunter Bioconfirm software was used to automate the identification of the tryptic fragments.

For LC-MS/MS analysis, collision-induced dissociation (CID) was performed with 1.3 *m/z* isolation width and a defined collision energy (based on the formula, $V = 0.036 \times (m/z) - 4.8$, unless specified otherwise). MS/MS spectra were extracted from the Agilent MassHunter Qualitative Analysis software and analyzed manually using mMass.[2] Fragment ions were identified and assigned based on the *m/z* error of the monoisotopic peak, ion intensity, and the isotopic distribution.

Trypsin Digestion

For variants to be digested, all eluted fractions were combined, concentrated (centrifugal filter, 10 kDa MWCO, 4 mL) and buffer exchanged into phosphate buffer (100 mM NaH_2PO_4, pH 7.0). The protein was incubated (37 °C) with trypsin (1:100 (w/w) trypsin:protein) in ammonium bicarbonate solution (50 mM, pH 8) for the required time (indicated in all figure captions), before being quenched with formic acid (final concentration 1% v/v) and analyzed by LC-MS.

High-Pressure Liquid Chromatography (HPLC)

Semi-preparative reverse-phase HPLC was performed using an Agilent 1200 series instrument, equipped with the Zorbax 300SB-C18 (9.4 mm x 250 mm, 5 μm) column and UV detector wavelength set at 215 nm. The mobile phase A was water with 0.1% trifluoroacetic acid, and mobile phase B was acetonitrile with 0.1% trifluoroacetic acid. Peptides were purified using the following method run with a flow rate of 4.0 mL min^{-1}: 10% B, 0-1 min; 10-50% B, 1-20 min; 50-90% B, 20-25 min; 90% B, 25-30 min; 90-10% B, 30-32 min. The desired peak was collected, and analyzed by LC-MS. The purified peptide was frozen (-80 °C) and lyophilized (Labconco FreeZone Freeze Dry System) to give a white solid which was dissolved in deionized water.

Ester-Selective Hydrazinolysis

The hydrazinolysis solution was freshly prepared using hydrazine solution (10 μL, 35 wt. % H_2O), PBS buffer (15 μL, pH 5.0) and adjusted with HCl solution (6 M) to pH 8, determined with pH paper. HPLC-purified peptide (at variable concentration, 0.1-3.0 mg mL^{-1}) or crude product from tryptic digestions was mixed with the hydrazinolysis solution in equal volumes and incubated (55 °C, 45 min) before immediately being subjected to LC-MS(/MS) analysis.

In vitro Enzyme Reconstitution

ThfB for *in vitro* reactions was purified under native conditions from BL21 (DE3) Δ*slyD* cells transformed with pJDK131 and stored (- 80 °C) in protein storage buffer until required. The ThfA variants were purified under denaturing conditions and stored (- 80 °C) in protein storage buffer until required. ATP (133 mM, in H_2O, pH 7.0) was prepared by addition of NaOH solution (6 M) and stored (- 80 °C) until required. DTT (1 M, in H_2O) was freshly prepared each day for *in vitro* reactions.

ThfA variants (10 μM) were mixed with ThfB (1 μM) in the *in vitro* reaction buffer with ATP (10 mM) and DTT (10 mM) before being incubated (37 ºC) in a thermocycler. For time course analysis, aliquots (10 μL) were withdrawn diluted with H_2O (20 μL) and quenched with formic acid (final concentration 1% v/v) and analyzed by LC-MS.

Table S3. Plasmids used in this study with the sequence of the protein expressed and cloning methodology.

Name	Parent Plasmid	Name and Sequence of Expressed Protein	Cloning Method
pBC108	pQE-80L	GFP labelled plasmid for golden gate cloning	See reference[1]
pThfA	pRSF Duet-1	**ThfA**<br>MGSSHHHHHHSQDPMSTAVTDAFP<br>LGRDENRNDQVTEWRPFGMRYGVQ<br>PTPIPVPLSDTKYDPDQQVLVVADGQ<br>PCAKIERAGTMRVTYPDGQKPGQSD<br>VEKD	See reference[3]
pHE18	pRSF Duet-1	**ThfA C76A**<br>MGSSHHHHHHSQDPMSTAVTDAFP<br>LGRDENRNDQVTEWRPFGMRYGVQ<br>PTPIPVPLSDTKYDPDQQVLVVADGQ<br>PAAKIERAGTMRVTYPDGQKPGQSD<br>VEKD	See reference[3]
pTGJ023	pQE-80L	**ThfA C76A**<br>MSGSHHHHHHGSSTAVTDAFPLGR<br>DENRNDQVTEWRPFGMRYGVQPTP<br>IPVPLSDTKYDPDQQVLVVADGQPAA<br>KIERAGTMRVTYPDGQKPGQSDVEK<br>D	See reference[4]
pBC262	pRSF Duet-1	**ThfB**<br>MTVLILTNPFDITADDVILRLTERGVP<br>VVRLDPADFPQQVVLHSEIGGNGWT<br>GTLTTPHRILDLSTVTGIWYRRPRKF<br>RLPAQMSQAEYEFAATEARRGFGGII<br>NSLTGWINHPSAIGRAEYKPYQLHHA<br>VQAGLNVPRTLITNDPKQAKGWCAR<br>VGDVVYKPLSAPSWLENGDTYVVFT<br>TPITPDQWGDPAIGRTAHMFQQRLD<br>KEFEVRLTMVDGKAFPAAIHAHSDAA<br>RIDWRSDYDALTYSIPTVPQRVLTGA<br>RDLLRRLHLRYAALDFIVSPDGRWHF<br>LEVNPNGQYGWIEEHTGQPISDAIAD<br>ALTRKEN	See reference[4]
pBC273	pQE-80L	**ThfA T84V/D103N**<br>MSGSHHHHHHGSSTAVTDAFPLGR<br>DENRNDQVTEWRPFGMRYGVQPTP<br>IPVPLSDTKYDPDQQVLVVADGQPAA<br>KIERAGVMRVTYPDGQKPGQSDVEK<br>N	See reference[4]

pBC274	pQE-80L	**ThfA T88V/D99N**<br>MSGSHHHHHHGSSTAVTDAFPLGR<br>DENRNDQVTEWRPFGMRYGVQPTP<br>IPVPLSDTKYDPDQQVLVVADGQPAA<br>KIERAGTMRVVYPDGQKPGQSNVEK<br>D	See reference[4]
pJDK131	pQE-80L	**His$_6$-ThfB**<br>MRGSHHHHHHGSMTVLILTNPFDITA<br>DDVILRLTERGVPVVRLDPADFPQQV<br>VLHSEIGGNGWTGTLTTPHRILDLST<br>VTGIWYRRPRKFRLPAQMSQAEYEF<br>AATEARRGFGGIINSLTGWINHPSAIG<br>RAEYKPYQLHHAVQAGLNVPRTLITN<br>DPKQAKGWCARVGDVVYKPLSAPS<br>WLENGDTYVVFTTPITPDQWGDPAI<br>GRTAHMFQQRLDKEFEVRLTMVDGK<br>AFPAAIHAHSDAARIDWRSDYDALTY<br>SIPTVPQRVLTGARDLLRRLHLRYAA<br>LDFIVSPDGRWHFLEVNPNGQYGWI<br>EEHTGQPISDAIADALTRKEN	See reference[3]
pBC001	pQE-80L	**ThfB**<br>MTVLILTNPFDITADDVILRLTERGVP<br>VVRLDPADFPQQVVLHSEIGGNGWT<br>GTLTTPHRILDLSTVTGIWYRRPRKF<br>RLPAQMSQAEYEFAATEARRGFGGII<br>NSLTGWINHPSAIGRAEYKPYQLHHA<br>VQAGLNVPRTLITNDPKQAKGWCAR<br>VGDVVYKPLSAPSWLENGDTYVVFT<br>TPITPDQWGDPAIGRTAHMFQQRLD<br>KEFEVRLTMVDGKAFPAAIHAHSDAA<br>RIDWRSDYDALTYSIPTVPQRVLTGA<br>RDLLRRLHLRYAALDFIVSPDGRWHF<br>LEVNPNGQYGWIEEHTGQPISDAIAD<br>ALTRKEN<br><br>**ThfM**<br>MTTQHTSSPRALADKLAHDGKLRDP<br>AWIKAFASIPRHLFLPHIYQPHPDGGF<br>TLASTPEELAYQDEGWTTQINGTFPT<br>PKDGEPIQGQPTSSSSAPGLMAVML<br>EALDVTDGVRVLEVGTGTGYNAALL<br>CHRLGDQHVVTVEVDPVLAEQAQQR<br>LAEVGYRPIVHVGDGADGYPPGAPY<br>DRVIVTCALTSLPWKLIEQTRQGGVL<br>VVPVWRGHLPAGLMLRLTATGGSLA<br>RGQVLDAYGGFMPARNTPPADAVQL<br>LHRHQAAADEGGTARTTSLTTGTSG	See reference[3]

		EPWAWFAALVVPDAVSLGTIHDDGP PRHWLLTEDSWCYLTPDGRVVQGG RRHLWDEIEAAHSTWEQLGQPARH QCQVIIDPDGARLTYQGREWPILL	
pTGJ009	pRSF Duet-1	**ThfB**<br>MTVLILTNPFDITADDVILRLTERGVP VVRLDPADFPQQVVLHSEIGGNGWT GTLTTPHRILDLSTVTGIWYRRPRKF RLPAQMSQAEYEFAATEARRGFGGII NSLTGWINHPSAIGRAEYKPYQLHHA VQAGLNVPRTLITNDPKQAKGWCAR VGDVVYKPLSAPSWLENGDTYVVFT TPITPDQWGDPAIGRTAHMFQQRLD KEFEVRLTMVDGKAFPAAIHAHSDAA RIDWRSDYDALTYSIPTVPQRVLTGA RDLLRRLHLRYAALDFIVSPDGRWHF LEVNPNGQYGWIEEHTGQPISDAIAD ALTRKEN<br><br>**ThfM**<br>MTTQHTSSPRALADKLAHDGKLRDP AWIKAFASIPRHLFLPHIYQPHPDGGF TLASTPEELAYQDEGWTTQINGTFPT PKDGEPIQGQPTSSSSAPGLMAVML EALDVTDGVRVLEVGTGTGYNAALL CHRLGDQHVVTVEVDPVLAEQAQQR LAEVGYRPIVHVGDGADGYPPGAPY DRVIVTCALTSLPWKLIEQTRQGGVL VVPVWRGHLPAGLMLRLTATGGSLA RGQVLDAYGGFMPARNTPPADAVQL LHRHQAAADEGGTARTTSLTTGTSG EPWAWFAALVVPDAVSLGTIHDDGP PRHWLLTEDSWCYLTPDGRVVQGG RRHLWDEIEAAHSTWEQLGQPARH QCQVIIDPDGARLTYQGREWPILL	Insert amplified from pBC001 with primers oTGJ017/oTGJ018 and digested with BsaI_HFv2 in rCutSmart buffer. Vector (pRSFDuet-1) digested with NcoI/HindIII in r2.1 buffer. Digested fragments ligated.
pTGJ056	pRSF Duet-1	**ThfM**<br>MTTQHTSSPRALADKLAHDGKLRDP AWIKAFASIPRHLFLPHIYQPHPDGGF TLASTPEELAYQDEGWTTQINGTFPT PKDGEPIQGQPTSSSSAPGLMAVML EALDVTDGVRVLEVGTGTGYNAALL CHRLGDQHVVTVEVDPVLAEQAQQR LAEVGYRPIVHVGDGADGYPPGAPY DRVIVTCALTSLPWKLIEQTRQGGVL VVPVWRGHLPAGLMLRLTATGGSLA RGQVLDAYGGFMPARNTPPADAVQL LHRHQAAADEGGTARTTSLTTGTSG	Insert amplified from pTGJ009 with primers oTGJ115/oTGJ018 and digested with BsaI_HFv2 in rCutSmart buffer. Vector (pRSFDuet-1) digested with NcoI/HindIII in r2.1 buffer. Digested fragments ligated.

		EPWAWFAALVVPDAVSLGTIHDDGP<br>PRHWLLTEDSWCYLTPDGRVVQGG<br>RRHLWDEIEAAHSTWEQLGQPARH<br>QCQVIIDPDGARLTYQGREWPILL	
pBC045	pQE-80L	**AmdA**<br>MSGSHHHHHHGSPVHQHERPTETG<br>AFPSSGHFPLGRRFGMVDTAPEPAS<br>PVRPFGLTLGTRPRQVTPLNPADIGY<br>DEDAQMGLMRDDGGQLVPMSRHTD<br>GQTNTVTDGGDGRYTNKDSDTDHR<br>ED<br><br>**SUMO-AmdB**<br>MSDSEVNQEAKPEVKPEVKPETHINL<br>KVSDGSSEIFFKIKKTTPLRRLMEAFA<br>KRQGKEMDSLRFLYDGIRIQADQTPE<br>DLDMEDNDIIEAHREQIGGSVGAARS<br>VLVLTDRFDPTADRVVEELNSRDTTV<br>VRIDVADFPERLSVSAELNGGPWSA<br>AWLQTARRSVNLADVSGIYYRRPTG<br>FDFHPDLSADERRWSAVQARMGFG<br>GLLAAVGPWLNHPHHIGYAEYKPAQ<br>LGAAVACGLPVPRTLVTNDPGRARA<br>FVTGVGRAVYKPFGGHGVTDTEGYR<br>HVFASVVTPEQCNDPNIARTMHMFQ<br>QWVPKSYEVRLTVVDGRFFAARIDT<br>ESATAYVDWRADYESLTYTAIETPNF<br>VQSRVNELLSTLNLRFGALDFVVAPD<br>GKWWFLECNPNGQWAWIEDQTGM<br>PIAAAIADALEGYDKT	See reference[1]
pBC315	pQE-80L	**Trivalent ThfA**<br>MSGSHHHHHHGSSTAVTDAFPLGR<br>DENRNDQVTEWRPFGMRYGVQPTP<br>IPVPLSDTKYDPDQQVLVVADGQPC<br>AKIERAGTMRVTYPDGQKPGQSDVE<br>KDSGTMRVTYPDGQKPGQSDVEKD<br>SGTMRVTYPDGQKPGQSDVEKD	See reference[4]
pWC102	pQE-80L	**PanA**<br>MNKAMATAKEVSELEALAAVDATHA<br>TRGVLGNDAEGITLLPLCFKPICIPTLP<br>PLTGGHA<br><br>**PanB**<br>MPEVSDPTHIGTGACADAPVFRHLA<br>PDVHIAGIDGHVVVLKLSDDRYFNLS<br>YKHSQGLRRLIGWRVDGDIPGDVLLA<br>TQAMFDAQGILAPGAHATPDPRIAKR	See reference[5]

DMSPRLGFDAWLAMPADVTRESRV
RDVMRAGYWLWQAQRVTRRARMR
GVVDMVARSQSGVRTQYGAPQDYL
PYVAAMHSAALVYPQCSPCLPWYAA
LTAWCARDGLRLRLVIGVQRQPFYA
HAWTETDARVIGDDLRRREQLAVIYE
TPV

PanC
MKLARLRPGPAERHEPFPDAVVMRG
LDGVPVARIRPDADSTRLHSSGQSEL
LAWYISGTLFLKEDGREVAPGMDVA
RLALESPERFVERYWGHYRVIVHDR
WRGTTLVLCDPCGQCPTYYRFCRD
GAVHVGADPAALVDDHPSFDSLALK
SFLVHGDLGGVQCALYDVERLPLGH
ALWIPERGAPQTRVVWWPDWRMAD
HDAQNVASGEGLLRLLRRSVERSVG
DRPAVLELSGGVESTSLALALAESGR
ADTCTAINHRDPSSPSSDESHHAGS
VARHVRMAFERLDIDCAAFSPSPSSA
IPRLARPALHLCMLAQLDAVRERLST
YSGHRLVNGHGGDAIFLAPPPNGAF
LDAFADGQWRRAFSAWRDLAVMQR
LPLWWVAQGAFAQLGQRHANPDAA
LSTGLLTPAARIADTPRTEPLASAWL
SRWALRVRPGKRAQVLGILGNLRDV
EVQPSLAPAGTVFPFLTQPMVEAGL
KMPTYRLFNAMHNRLPLRRAAYLAS
GLPNLWRRDKGVLTGMVGAGAAAN
LAHVEEVCLDGYCAASGWIDRDACR
RAIGRVAHHHLESMTTIRRLYAIEMFV
RGWRAGAV

PanD
MTRQAGWQALCFVLRRLWARETQT
SRHRVWVVMAALAAAFMLALRLVAP
WVLGLAVDALTGERAVGYALPLATA
YVVLWSAAAIGERGKDLLIQRAMEDL
RRRTALGYLSRLVGLPAHRAREVNA
GQTLDCLFRVEVALPVIVTGLVFGLLP
LAVQVLATGIILMVNFGAMYGVLLGA
TVVAYVLTLRPSLARVTRREAQANDI
SRATMGALADTLTQLECVKTFAQEP
AEIGRLSQRLRTRYVAALATATTAQV
TSACQMAIMALGVSAMTLLAVRDVM
RGAEPVGTLVQINAYLLQFALPAAML

		GALVSQVARALVSVDEHLRDATSGP MTDDVEGVEDVDAAKEANESGQGG ATAAAGALAVPSPRAPCIELDQVGVT LGDGTPVVARVSLRIRAGEYVAIVGP SGAGKSTLLRLMTGLTLPTQGQVRID GRALNGVAATALRRVTGFVTQDCRL FDRSVFENVAYRAGLLNAALPDDPAI RDEFMRVATLAEVPDVISVMGLSGG ERQRVNIARALWGRPRWLLFDEPTA ALDALTEARILNRLAHERAGATCVVV AHRLAAICRADRIVVMDQGHIVDVGT HSALLARGGLYARMWQAQQADLGA EAVLG	
pTGJ007	pQE-80L	**ThfA1** MIERAGTMRVTYPDGQKPGQSDVEK DGRGSTAVTDAFPLGRDENRNDQVT EWRPFGMRYGVQPTPIPVPLSDTKY DPDQQVLVVADGQPAAKIERAGHHH HHH	Insert1 and Insert2 amplified from pHE18 with primers oTGJ013/oTGJ011 and oTGJ008/oTGJ014, respectively. Golden Gate Assembly with BsaI_Hfv2 of Insert1, Insert2 and the vector (pBC108) pre-digested with EcoRI/HindIII in r3.1 buffer.
pTGJ066	pQE-80L	**ThfA2** MIERAGVMRVTYPDGQKPGQSDVEK NGRGSTAVTDAFPLGRDENRNDQVT EWRPFGMRYGVQPTPIPVPLSDTKY DPDQQVLVVADGQPAAKIERAGHHH HHH	Insert1 amplified from pBC273 with primers oTGJ008/oTGJ130 and Insert2 amplified from pHE18 with primers oTGJ011/oTGJ013. Insert1 and Insert2 each digested with BsaI_HFv2 in rCutSmart buffer. Vector (pBC108) digested with EcoRI/HindIII in r3.1 buffer. Digested fragments ligated.
pTGJ067	pQE-80L	**ThfA3** MIERAGTMRVVYPDGQKPGQSNVEK DGRGSTAVTDAFPLGRDENRNDQVT EWRPFGMRYGVQPTPIPVPLSDTKY DPDQQVLVVADGQPAAKIERAGHHH HHH	Insert1 amplified from pBC274 with primers oTGJ008/oTGJ131 and Insert2 amplified from pHE18 with primers oTGJ011/oTGJ013. Insert1 and Insert2 each digested with BsaI_HFv2 in rCutSmart buffer. Vector (pBC108) digested with EcoRI/HindIII in r3.1 buffer. Digested fragments ligated.
pTGJ068	pQE-80L	**ThfA4** MIERAGVMRVVYPDGQKPGQSNVE KNGRGSTAVTDAFPLGRDENRNDQV TEWRPFGMRYGVQPTPIPVPLSDTK YDPDQQVLVVADGQPAAKIERAGHH HHHH	Insert1 amplified from pTGJ007 with primers oTGJ134/oTGJ134, Insert2 amplified from pBC274 with primers oTGJ132/oTGJ133 and Insert3 amplified from pHE18 with primers oTGJ011/oTGJ013. Insert1, Insert2 and Insert3 each digested with BsaI_HFv2 in rCutSmart buffer.

			Vector (pBC108) digested with XhoI/HindIII in r2.1 buffer. Digested fragments ligated.
pTGJ005	pQE-80L	**ThfA5** MIERAGTMRVTYPDGQKPGQSDVEKDGRGSTAVTDAFPLGRDENRNDQVTEWRPFGMRYGVQPTPIPVPLSDTKYDPDQQVLVVADGQPAAKIERAGHHHHHH	Insert1 and Insert2 amplified from pHE18 with primers oTGJ010/oTGJ011 and oTGJ008/oTGJ009, respectively. Golden Gate Assembly with BsaI_Hfv2 of Insert1, Insert2 and the vector (pBC108) pre-digested with EcoRI/HindIII in r3.1 buffer.
pTGJ004	pQE-80L	**ThfA6** MIERAGTMRVTYPDGQKPGQSDVEKDGSSTAVTDAFPLGRDENRNDQVTEWRPFGMRYGVQPTPIPVPLSDTKYDPDQQVLVVADGQPAAKIERAGHHHHHH	Insert1 and Insert2 amplified from pThfA with primers oTGJ010/oTGJ011 and oTGJ008/oTGJ009, respectively. Golden Gate Assembly with BsaI_Hfv2 of Insert1, Insert2 and the vector (pBC108) pre-digested with EcoRI/HindIII in r3.1 buffer.
pTGJ027	pQE-80L	**ThfA7** MIERAGTMRVTYPDGQKPGQSDVEKDGSSTAVTDAFPLGRDENRNDQVTEWRPFGMRYGVQPTPIPVPLSDTKYDPDQQVLVVADGQPCAKIERAGHHHHHH	Insert1 amplified from pHE18 with primers oTGJ008/oTGJ014. Insert2 amplified from CEN.PK (wt) genomic DNA with primers oTGJ059/oTGJ060. Golden Gate Assembly with BsaI_Hfv2 of Insert1, Insert2 and the vector (pBC108) pre-digested with EcoRI/HindIII in r3.1 buffer.
pTGJ037	pQE-80L	**ThfA8** MIERAGTMRVTYPDGQKPGQSDVEKDGRGSTAVTDAFPLGRDENRNDQVTEWRPFGMRYGVQPTPIPVPLSDTKYDPDQQVLVVADGHHHHHH	Insert1 and Insert2 amplified from pHE18 with primers oTGJ013/oTGJ080 and oTGJ008/oTGJ014, respectively. Golden Gate Assembly with BsaI_Hfv2 of Insert1, Insert2 and the vector (pBC108) pre-digested with EcoRI/HindIII in r3.1 buffer.
pTGJ038	pQE-80L	**ThfA9** MIERAGTMRVTYPDGQKPGQSDVEKDGRGSTAVTDAFPLGRDENRNDQVTEWRPFGMRYGVQPTPIPVPLSDTKYDPHHHHHH	Insert1 and Insert2 amplified from pHE18 with primers oTGJ013/oTGJ081 and oTGJ008/oTGJ014, respectively. Golden Gate Assembly with BsaI_Hfv2 of Insert1, Insert2 and the vector (pBC108) pre-digested with EcoRI/HindIII in r3.1 buffer.
pTGJ039	pQE-80L	**ThfA10**	Insert1 and Insert2 amplified from pHE18 with primers oTGJ013/oTGJ082 and

		MIERAGTMRVTYPDGQKPGQSDVEK DGRGSTAVTDAFPLGRDENRNDQVT EWRPFGMRYGVQPTPIPHHHHHH	oTGJ008/oTGJ014, respectively. Golden Gate Assembly with BsaI_Hfv2 of Insert1, Insert2 and the vector (pBC108) pre-digested with EcoRI/HindIII in r3.1 buffer.
pTGJ040	pQE-80L	**ThfA11**<br>MIERAGTMRVTYPDGQKPGQSDVEK DGRGSTAVTDAFPLGRDENRNDQVT EWRPFGMHHHHHH	Insert1 and Insert2 amplified from pHE18 with primers oTGJ013/oTGJ083 and oTGJ008/oTGJ014, respectively. Golden Gate Assembly with BsaI_Hfv2 of Insert1, Insert2 and the vector (pBC108) pre-digested with EcoRI/HindIII in r3.1 buffer.
pTGJ041	pQE-80L	**ThfA12**<br>MIERAGTMRVTYPDGQKPGQSDVEK DGRGSTAVTDAFPLGRDENRNDHH HHHH	Insert1 and Insert2 amplified from pHE18 with primers oTGJ013/oTGJ084 and oTGJ008/oTGJ014, respectively. Golden Gate Assembly with BsaI_Hfv2 of Insert1, Insert2 and the vector (pBC108) pre-digested with EcoRI/HindIII in r3.1 buffer.
pTGJ030	pQE-80L	**AmdA1**<br>MSRHTDGQTNTVTDGGDGRYTNKD SDTDHREDGRGPVHQHERPTETGA FPSSGHFPLGRRFGMVDTAPEPASP VRPFGLTLGTRPRQVTPLNPADIGYD EDAQMGLMRDDGGQLVPMSRHTHH HHHH<br><br>**SUMO-AmdB**<br>MSDSEVNQEAKPEVKPEVKPETHINL KVSDGSSEIFFKIKKTTPLRRLMEAFA KRQGKEMDSLRFLYDGIRIQADQTPE DLDMEDNDIIEAHREQIGGSVGAARS VLVLTDRFDPTADRVVEELNSRDTTV VRIDVADFPERLSVSAELNGGPWSA AWLQTARRSVNLADVSGIYYRRPTG FDFHPDLSADERRWSAVQARMGFG GLLAAVGPWLNHPHHIGYAEYKPAQ LGAAVACGLPVPRTLVTNDPGRARA FVTGVGRAVYKPFGGHGVTDTEGYR HVFASVVTPEQCNDPNIARTMHMFQ QWVPKSYEVRLTVVDGRFFAARIDT ESATAYVDWRADYESLTYTAIETPNF VQSRVNELLSTLNLRFGALDFVVAPD GKWWFLECNPNGQWAWIEDQTGM PIAAAIADALEGYDKT	Insert1, Insert2 and Insert3 amplified from pBC045 with primers oTGJ066/oTGJ067, oTGJ064/oTGJ065 and oTGJ068/oTGJ069, respectively. Golden Gate Assembly with BsaI_Hfv2 of Insert1, Insert2, Insert3 and the vector (pBC108).

pTGJ063	pQE-80L	**AmdA1** MSRHTDGQTNTVTDGGDGRYTNKD SDTDHREDGRGPVHQHERPTETGA FPSSGHFPLGRRFGMVDTAPEPASP VRPFGLTLGTRPRQVTPLNPADIGYD EDAQMGLMRDDGGQLVPMSRHTHH HHHH	Insert amplified from pTGJ030 with primers pQE80_fwd/oTGJ127. Insert and vector (pBC108) each digested with EcoRI/HindIII in r3.1 buffer. Digested fragments ligated.
pTGJ064	pRSF Duet-1	**SUMO-AmdB** MSDSEVNQEAKPEVKPEVKPETHINL KVSDGSSEIFFKIKKTTPLRRLMEAFA KRQGKEMDSLRFLYDGIRIQADQTPE DLDMEDNDIIEAHREQIGGSVGAARS VLVLTDRFDPTADRVVEELNSRDTTV VRIDVADFPERLSVSAELNGGPWSA AWLQTARRSVNLADVSGIYYRRPTG FDFHPDLSADERRWSAVQARMGFG GLLAAVGPWLNHPHHIGYAEYKPAQ LGAAVACGLPVPRTLVTNDPGRARA FVTGVGRAVYKPFGGHGVTDTEGYR HVFASVVTPEQCNDPNIARTMHMFQ QWVPKSYEVRLTVVDGRFFAARIDT ESATAYVDWRADYESLTYTAIETPNF VQSRVNELLSTLNLRFGALDFVVAPD GKWWFLECNPNGQWAWIEDQTGM PIAAAIADALEGYDKT	Insert amplified from pTGJ030 with primers oTGJ068/oTGJ069 and digested with BsaI_HFv2 in rCutSmart buffer. Vector (pRSFDuet-1) digested with NcoI/HindIII in r2.1 buffer. Digested fragments ligated.
pTGJ011	pQE-80L	**ThfA13** MIERAGTMRVTYPDGQKPGQSDVEK DSGTMRVTYPDGQKPGQSDVEKDG SSTAVTDAFPLGRDENRNDQVTEWR PFGMRYGVQPTPIPVPLSDTKYDPD QQVLVVADGQPCAKIERAGHHHHHH	Obtained as a side product of cloning pTGJ006.
pTGJ006	pQE-80L	**ThfA14** MIERAGTMRVTYPDGQKPGQSDVEK DSGTMRVTYPDGQKPGQSDVEKDS GTMRVTYPDGQKPGQSDVEKDGSS TAVTDAFPLGRDENRNDQVTEWRPF GMRYGVQPTPIPVPLSDTKYDPDQQ VLVVADGQPCAKIERAGHHHHHH	Insert1 and Insert2 amplified from pBC315 with primers oTGJ010/oTGJ011 and oTGJ008/oTGJ012, respectively. Golden Gate Assembly with BsaI_Hfv2 of Insert1, Insert2 and the vector (pBC108) pre-digested with EcoRI/HindIII in r3.1 buffer.
pTGJ008	pQE-80L	**ThfA15** MSGSHHHHHHGSSTAVTDAFPLGR DENRNDQVTEWRPFGMRYGVQPTP IPVPLSDTKYDPDQQVLVVADGQPC AKIERAGTMRVTYPDGQKPGQSDVE KDSGTMRVTYPDGQKPGQSDVEKD SGTMRVTYPDGQKPGQSDVEKDGS STAVTDAFPLGRDENRNDQVTEWRP	Insert1 and Insert2 amplified from pBC315 with primers oTGJ010/oTGJ016 and oTGJ015/oTGJ011, respectively. Golden Gate Assembly with BsaI_Hfv2 of Insert1, Insert2 and the vector (pBC108).

		FGMRYGVQPTPIPVPLSDTKYDPDQ QVLVVADGQPCAKIERAGHHHHHH	
pTGJ010	pQE-80L	**ThfA16 + PanBCD** MNKAMATAKEVSELEALAAVDATHA TRGVLGNDAEGITLLPLCFKPICIPTLP PLTGGHAAGTMRVTYPDGQKPGQS DVEKDGSSTAVTDAFPLGRDENRND QVTEWRPFGMRYGVQPTPIPVPLSD TKYDPDQQVLVVADGQPAAKIERAG HHHHHH	Insert1 amplified from pWC102 with primers oTGJ002/oTGJ025 and digested with XhoI/BsaI_HFv2 in r2.1 buffer. Insert2 amplified from pTGJ005 with primers oTGJ024/oTGJ023 and digested with BsaI_HFv2/HindIII in r2.1 buffer. Vector (pWC102) digested with XhoI/HindIII in r2.1 buffer. Digested fragments ligated.
pTGJ031	pQE-80L	**ThfA17 + PanBCD** MNKAMATAKEVSELEALAAVDATHA TRGVLGNDAEGITLLPLCFKPICIPTLP PLTGGHAAGTMRVTYPDGQKPGQS DVEKDGRGSTAVTDAFPLGRDENRN DQVTEWRPFGMRYGVQPTPIPVPLS DTKYDPDQQVLVVADGQPAAKIERA GHHHHHH	Insert1 amplified from pWC102 with primers oTGJ002/oTGJ025 and digested with XhoI/BsaI_HFv2 in r2.1 buffer. Insert2 amplified from pTGJ007 with primers oTGJ024/oTGJ023 and digested with BsaI_HFv2/HindIII in r2.1 buffer. Vector (pWC102) digested with XhoI/HindIII in r2.1 buffer. Digested fragments ligated.
pTGJ021	pQE-80L	**ThfA18 + PanBCD** MNKAMATAKEVSELEALAAVDATHA TRGVLGNDAEGITLLPLCFKPICIPTLP PLTGGHADVEKDGRGSTAVTDAFPL GRDENRNDQVTEWRPFGMRYGVQP TPIPVPLSDTKYDPDQQVLVVADGQP AAKIERAGHHHHHH	Insert1 amplified from pWC102 with primers oTGJ002/oTGJ051 and digested with XhoI/BsaI_HFv2 in r2.1 buffer. Insert2 amplified from pTGJ007 with primers oTGJ052/oTGJ023 and digested with BsaI_HFv2/HindIII in r2.1 buffer. Vector (pWC102) digested with XhoI/HindIII in r2.1 buffer. Digested fragments ligated.
pTGJ044	pQE-80L	**ThfA19 + PanBCD** MNKAMATAKEVSELEALAAVDATHA TRGVLGNDAEGITLLPLCFKPICIPTLP PLTGDVEKDGRGSTAVTDAFPLGRD ENRNDQVTEWRPFGMRYGVQPTPIP VPLSDTKYDPDQQVLVVADGQPAAKI ERAGHHHHHH	Insert1 amplified from pWC102 with primers oTGJ002/oTGJ091 and digested with XhoI/BsaI_HFv2 in r2.1 buffer. Insert2 amplified from pTGJ007 with primers oTGJ052/oTGJ023 and digested with BsaI_HFv2/HindIII in r2.1 buffer. Vector (pWC102) digested with XhoI/HindIII in r2.1 buffer. Digested fragments ligated.
pTGJ045	pQE-80L	**ThfA20 + PanBCD** MNKAMATAKEVSELEALAAVDATHA TRGVLGNDAEGITLLPLCFKPICIPTLP PDVEKDGRGSTAVTDAFPLGRDENR NDQVTEWRPFGMRYGVQPTPIPVPL	Insert1 amplified from pWC102 with primers oTGJ002/oTGJ092 and digested with XhoI/BsaI_HFv2 in r2.1 buffer. Insert2 amplified from pTGJ007 with primers

		SDTKYDPDQQVLVVADGQPAAKIER AGHHHHHH	oTGJ052/oTGJ023 and digested with BsaI_HFv2/HindIII in r2.1 buffer. Vector (pWC102) digested with XhoI/HindIII in r2.1 buffer. Digested fragments ligated.
pTGJ046	pQE-80L	**ThfA21 + PanBCD** MNKAMATAKEVSELEALAAVDATHA TRGVLGNDAEGITLLPLCFKPICIPTD VEKDGRGSTAVTDAFPLGRDENRND QVTEWRPFGMRYGVQPTPIPVPLSD TKYDPDQQVLVVADGQPAAKIERAG HHHHHH	Insert1 amplified from pWC102 with primers oTGJ002/oTGJ093 and digested with XhoI/BsaI_HFv2 in r2.1 buffer. Insert2 amplified from pTGJ007 with primers oTGJ052/oTGJ023 and digested with BsaI_HFv2/HindIII in r2.1 buffer. Vector (pWC102) digested with XhoI/HindIII in r2.1 buffer. Digested fragments ligated.
pTGJ047	pQE-80L	**ThfA22 + PanBCD** MNKAMATAKEVSELEALAAVDATHA TRGVLGNDAEGITLLPLCFKPICDVEK DGRGSTAVTDAFPLGRDENRNDQVT EWRPFGMRYGVQPTPIPVPLSDTKY DPDQQVLVVADGQPAAKIERAGHHH HHH	Insert1 amplified from pWC102 with primers oTGJ002/oTGJ094 and digested with XhoI/BsaI_HFv2 in r2.1 buffer. Insert2 amplified from pTGJ007 with primers oTGJ052/oTGJ023 and digested with BsaI_HFv2/HindIII in r2.1 buffer. Vector (pWC102) digested with XhoI/HindIII in r2.1 buffer. Digested fragments ligated.
pTGJ048	pQE-80L	**ThfA23 + PanBCD** MNKAMATAKEVSELEALAAVDATHA TRGVLGNDAEGITLLPLCFKDVEKDG RGSTAVTDAFPLGRDENRNDQVTE WRPFGMRYGVQPTPIPVPLSDTKYD PDQQVLVVADGQPAAKIERAGHHHH HH	Insert1 amplified from pWC102 with primers oTGJ002/oTGJ095 and digested with XhoI/BsaI_HFv2 in r2.1 buffer. Insert2 amplified from pTGJ007 with primers oTGJ052/oTGJ023 and digested with BsaI_HFv2/HindIII in r2.1 buffer. Vector (pWC102) digested with XhoI/HindIII in r2.1 buffer. Digested fragments ligated.
pTGJ070	pQE-80L	**ThfA24 + PanBCD** MNKAMATAKEVSELEALAAVDATHA TRGVLGNDAEGITLLPLCFKNVEKDG RGSTAVTDAFPLGRDENRNDQVTE WRPFGMRYGVQPTPIPVPLSDTKYD PDQQVLVVADGQPAAKIERAGHHHH HH	Insert1 amplified from pWC102 with primers oTGJ002/oTGJ137 and digested with XhoI/BsaI_HFv2 in r2.1 buffer. Insert2 amplified from pTGJ067 with primers oTGJ136/oTGJ023 and digested with BsaI_HFv2/HindIII in r2.1 buffer. Vector (pWC102) digested with XhoI/HindIII in r2.1 buffer. Digested fragments ligated.
pTGJ071	pQE-80L	**ThfA24 + PanBCD**	Insert1 amplified from pWC102 with primers oTGJ002/oTGJ137 and

	MNKAMATAKEVSELEALAAVDATHATRGVLGNDAEGITLLPLCFKNVEKNGRGSTAVTDAFPLGRDENRNDQVTEWRPFGMRYGVQPTPIPVPLSDTKYDPDQQVLVVADGQPAAKIERAGHHHHHH	digested with XhoI/BsaI_HFv2 in r2.1 buffer. Insert2 amplified from pTGJ068 with primers oTGJ138/oTGJ023 and digested with BsaI_HFv2/HindIII in r2.1 buffer. Vector (pWC102) digested with XhoI/HindIII in r2.1 buffer. Digested fragments ligated.

Table S4. Oligonucleotides used in this study.

Name	Sequence (5’→3’)
oTGJ002	GCTAATCTCGAGAAATCATAAAAAATTTATTTGCTTTGTG
oTGJ008	GCATGGTCTCTAATTCATTAAAGAGGAGAAATTAACTATGATCGAAAGAGCCGGC
oTGJ009	GCATGGTCTCTGATCCGTCTTTTTCCACGTCGC
oTGJ010	GCATGGTCTCTGATCCTCGACAGCGG
oTGJ011	GCATGGTCTCTAGCTTTAGTGATGGTGATGGTGATGGCCGGCTCTTTCGATTTT
oTGJ012	GCATGGTCTCTGATCCGTCCTTCTCTACGTCGCTC
oTGJ013	GCATGGTCTCTGACGAGGATCGACAGCGGTCAC
oTGJ014	GCATGGTCTCTCGTCCGTCTTTTTCCACGTCGC
oTGJ015	GCATGGTCTCTGAAGCTCGACAGCGGTCAC
oTGJ016	GCATGGTCTCTCTTCCGTCCTTCTCTACGTCGCTC
oTGJ017	GCATGGTCTCTCATGACCGTTCTCATCCTCAC
oTGJ018	CGATGGTCTCAAGCTTTTAGAGCAGGATCGG
oTGJ023	ATTCGGTCTCTCTAGCTTGGATTCTCACCAATAAAAAACG
oTGJ051	GCATTCGGTCTCTCGTCGGCATGGCCCCC
oTGJ052	TTAGCTGGTCTCTGACGTGGAAAAAGACGGAC
oTGJ059	GCATGGTCTCTGACGAGGATCGGACTCAGAAGTCAATC
oTGJ060	GCATGGTCTCTAGCTTTAGTGATGGTGATGGTGATGATACGTAGCACCACCAATCTG
oTGJ064	GCATGGTCTCTGACGAGGACCCGTACACCAGCAC
oTGJ065	GCATGGTCTCACATGATCAGTGATGGTGATGGTGATGCGTGTGACGACTCATCG
oTGJ066	GCATGGTCTCTAATTCATTAAAGAGGAGAAATTAACTATGAGTCGTCACACGGAC
oTGJ067	GCATGGTCTCTCGTCCATCCTCCCGATGGTCG
oTGJ068	TAACTGGTCTCTCATGTCGGACTCAGAAGTC
oTGJ069	AACTGGGTCTCTAGCTTTCATGTCTTGTCGTATCC
oTGJ080	GCATGGTCTCTAGCTTTAGTGATGGTGATGGTGATGGCCGTCTGCCACC
oTGJ081	GCATGGTCTCTAGCTTTAGTGATGGTGATGGTGATGGGGATCATACTTGGTGTCG
oTGJ082	GCATGGTCTCTAGCTTTAGTGATGGTGATGGTGATGAGGGATAGGCGTGGG
oTGJ083	GCATGGTCTCTAGCTTTAGTGATGGTGATGGTGATGCATTCCGAACGGGCG
oTGJ084	GCATGGTCTCTAGCTTTAGTGATGGTGATGGTGATGGTCGTTTCTGTTCTCGTCC
oTGJ091	GCATTCGGTCTCTCGTCCCCGGTCAGCGG
oTGJ092	GCATTCGGTCTCTCGTCCGGCGGCAACGTG
oTGJ093	GCATTCGGTCTCTCGTCCGTGGGAATGCAGATCG

oTGJ094	GCATTCGGTCTCTCGTCGCAGATCGGCTTGAAGC
oTGJ095	GCATTCGGTCTCTCGTCCTTGAAGCACAGCGGC
oTGJ115	GCATGGTCTCTCATGACCACACAGCACAC
oTGJ127	CTAATTAAGCTTTAGTGATGGTGATGGTGATGC
oTGJ130	GCATGGTCTCTCGTCCGTTTTTTTCCACGTCGCTC
oTGJ131	GCATGGTCTCTCGTCCGTCTTTTTCCACGTTGCTCTG
oTGJ132	GCATGGTCTCTCGTTATGAGAGTCGTTTATCCCGAC
oTGJ133	GCATGGTCTCTCGTCCGTTTTTTTCCACGTTGCTCTGC
oTGJ134	GTTAGAGGTCTCCTCGAGAAATCATAAAAAATTTATTTGCTTTGTG
oTGJ135	GCATGGTCTCTAACGCCGGCTCTTTCGATC
oTGJ136	TTAGCTGGTCTCCAACGTGGAAAAAGACGGAC
oTGJ137	GCATTCGGTCTCTCGTTGGCATGGCCCCC
oTGJ138	TTAGCTGGTCTCCAACGTGGAAAAAAACGGACG
pQE80_ fwd	CCCGAAAAGTGCCACCTG

Supplementary Figures

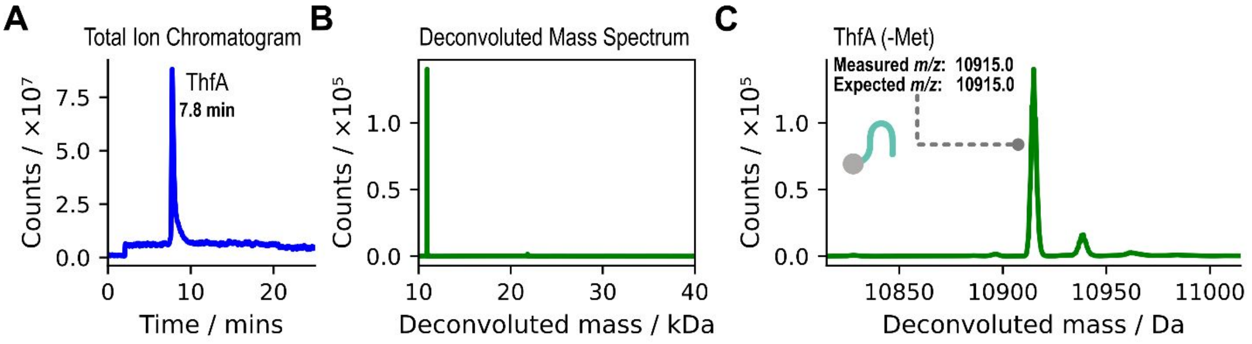

Figure S1. Mass spectrometry analysis of **ThfA** expressed in *E. coli* BL21 (DE3) *ΔslyD* cells transformed with pTGJ023, purified from the cell pellet under denaturing conditions. A) The total ion chromatogram of purified protein, with the retention time of the peak labelled. B) Deconvoluted mass spectrum of the major peak in the TIC. C) Magnified plot of the deconvoluted mass spectrum of the major peak showing **ThfA** with loss of Met1 from the protein mass. The gray circle in the cartoon represents the leader peptide.

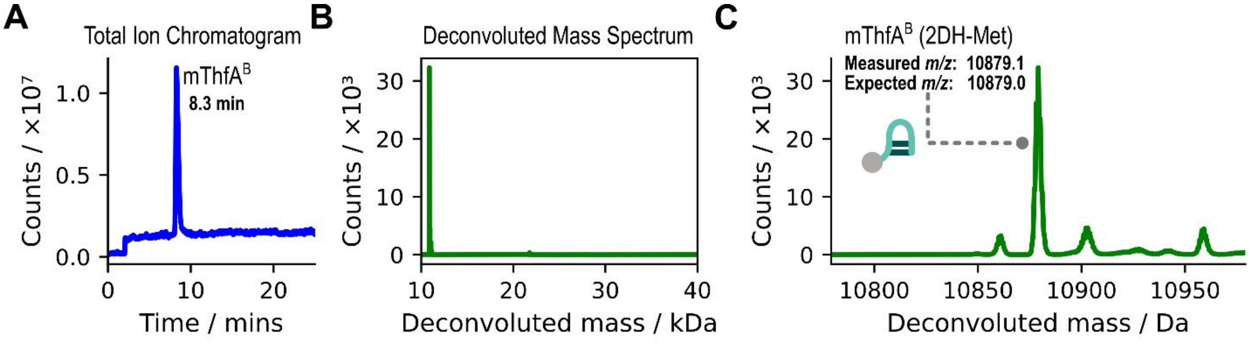

Figure S2. Mass spectrometry analysis of **mThfA^{B}** expressed in *E. coli* BL21 (DE3) *ΔslyD* cells transformed with pTGJ023 and pBC262, purified from the cell pellet under denaturing conditions. A) The total ion chromatogram of purified protein, with the retention time of the peak labelled. B) Deconvoluted mass spectrum of the major peak in the TIC. C) Magnified plot of the deconvoluted mass spectrum of the major peak showing **mThfA^{B}** with loss of Met1 and two-fold dehydration of the protein mass, indicative of the formation of two ester crosslinks. The gray circle in the cartoon represents the leader peptide.

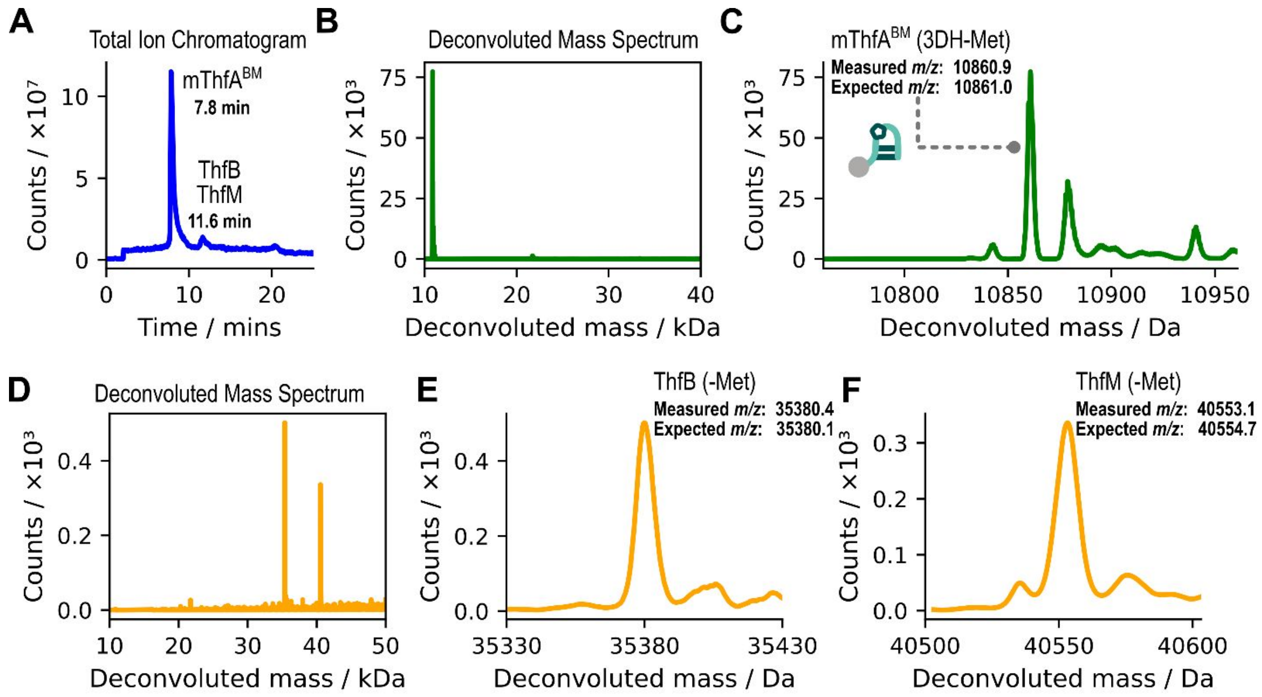

Figure S3. Mass spectrometry analysis of **mThfA^{BM}** expressed in *E. coli* BL21 (DE3) *ΔslyD* cells transformed with pTGJ023 and pTGJ009, purified from the cell pellet under denaturing conditions. A) The total ion chromatogram of purified protein, with the retention time of the peaks labelled. B) Deconvoluted mass spectrum of the major peak in the TIC. C) Magnified plot of the deconvoluted mass spectrum of the major peak showing **mThfA^{BM}** with loss of Met1 and two-fold dehydration of the protein mass, indicative of the formation of two ester crosslinks and an aspartimidylation. The gray circle in the cartoon represents the leader peptide. D) Deconvoluted mass spectrum of the minor peak in the TIC. E) Magnified plot of the deconvoluted mass spectrum of the minor peak showing **ThfB** with loss of Met1. F) Magnified plot of the deconvoluted mass spectrum of the minor peak showing **ThfM** with loss of Met1.

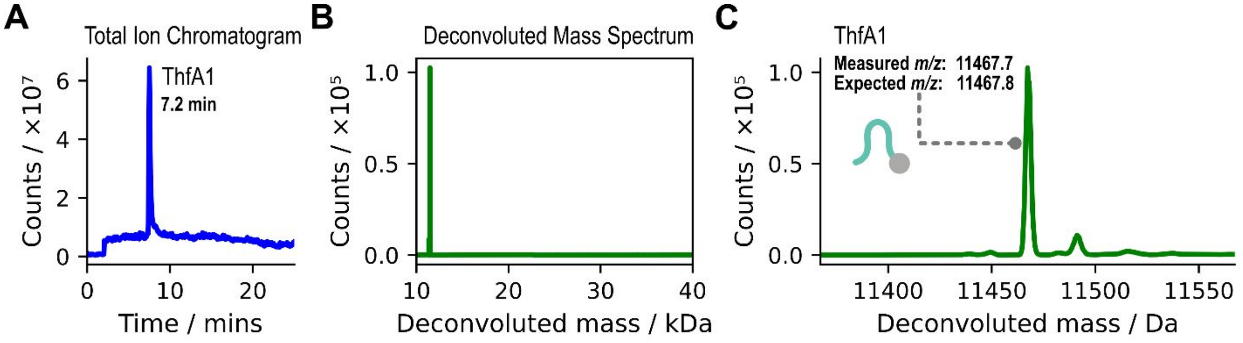

Figure S4. Mass spectrometry analysis of **ThfA1** expressed in *E. coli* BL21 (DE3) *ΔslyD* cells transformed with pTGJ007, purified from the cell pellet under denaturing conditions. A) The total ion chromatogram of purified protein, with the retention time of the peak labelled. B) Deconvoluted mass spectrum of the major peak in the TIC. C) Magnified plot of the deconvoluted mass spectrum of the major peak showing the unmodified protein mass of **ThfA1**. The gray circle in the cartoon represents the leader peptide.

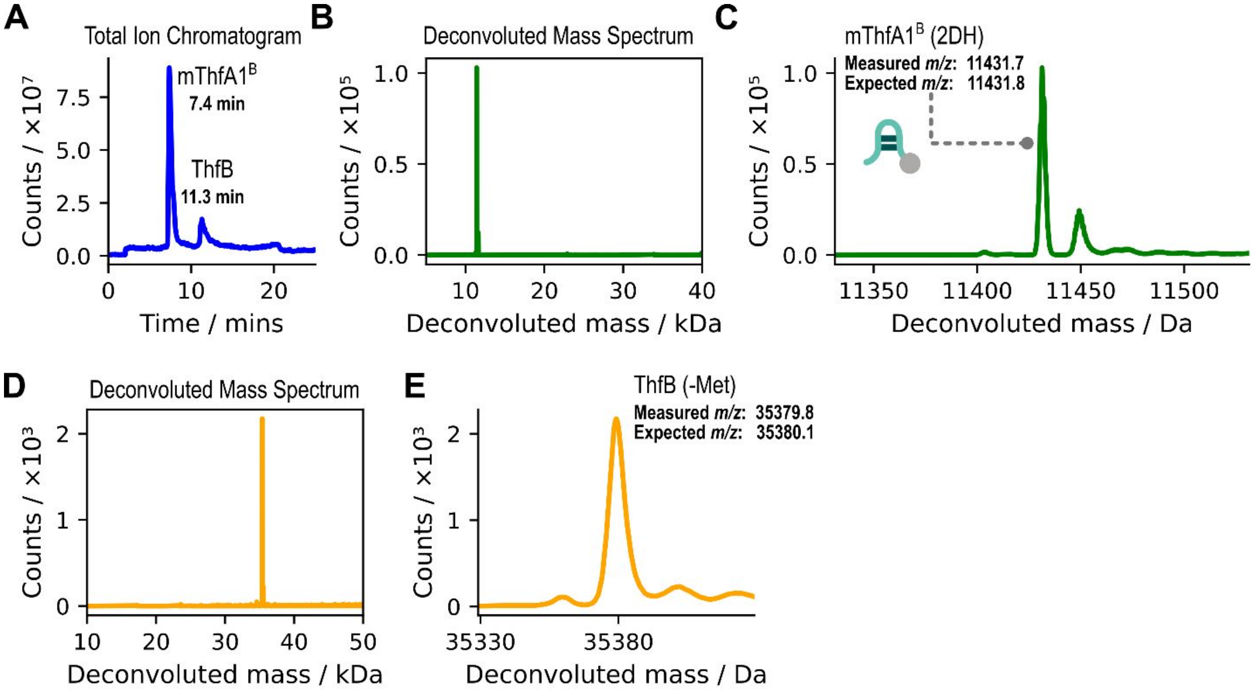

Figure S5. Mass spectrometry analysis of **mThfA1^{B}** expressed in *E. coli* BL21 (DE3) *ΔslyD* cells transformed with pTGJ007 and pBC262, purified from the cell pellet under denaturing conditions. A) The total ion chromatogram of purified protein, with the retention time of the peaks labelled. B) Deconvoluted mass spectrum of the major peak in the TIC. C) Magnified plot of the deconvoluted mass spectrum of the major peak showing **mThfA1^{B}** with two-fold dehydration of the protein mass, indicative of the formation of two ester crosslinks. The gray circle in the cartoon represents the leader peptide. D) Deconvoluted mass spectrum of the minor peak in the TIC. E) Magnified plot of the deconvoluted mass spectrum of the minor peak showing **ThfB** with loss of Met1.

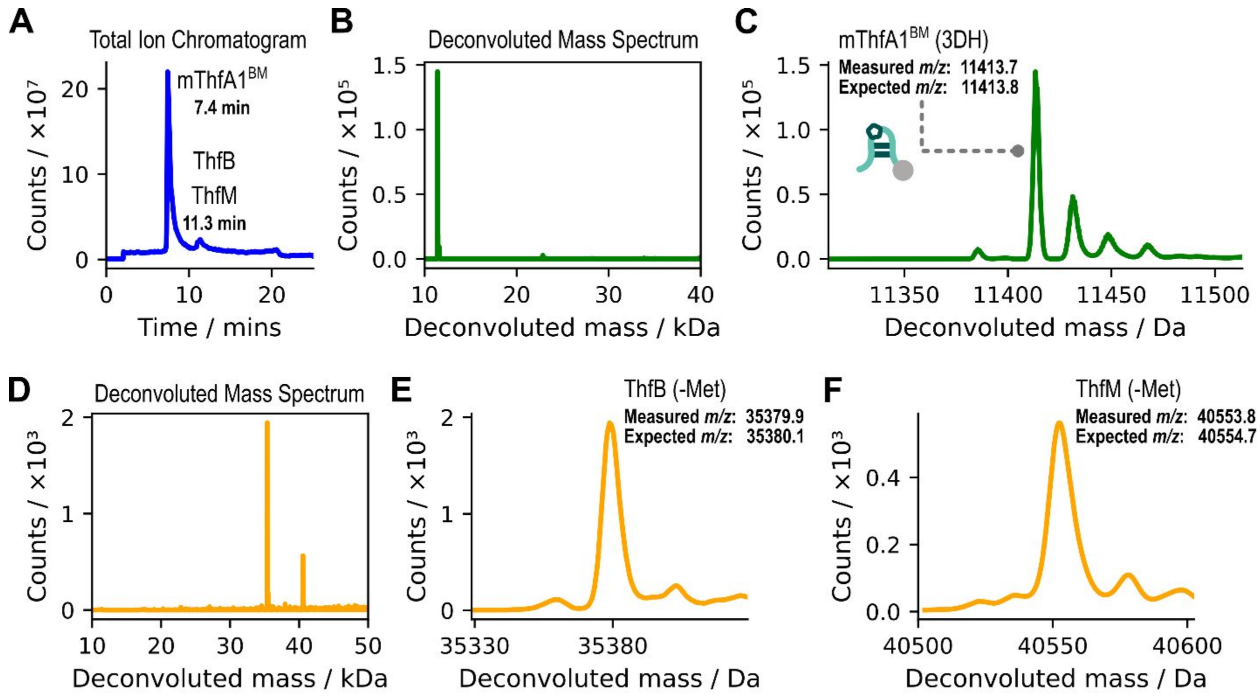

Figure S6. Mass spectrometry analysis of **mThfA1^{BM}** expressed in *E. coli* BL21 (DE3) *ΔslyD* cells transformed with pTGJ007 and pTGJ009, purified from the cell pellet under denaturing conditions. A) The total ion chromatogram of purified protein, with the retention time of the peaks labelled. B) Deconvoluted mass spectrum of the major peak in the TIC. C) Magnified plot of the deconvoluted mass spectrum of the major peak showing **mThfA1^{BM}** with three-fold dehydration of the protein mass, indicative of the formation of two ester crosslinks and an aspartimidylation. The gray circle in the cartoon represents the leader peptide. D) Deconvoluted mass spectrum of the minor peak in the TIC. E) Magnified plot of the deconvoluted mass spectrum of the minor peak showing **ThfB** with loss of Met1. F) Magnified plot of the deconvoluted mass spectrum of the minor peak showing **ThfM** with loss of Met1.

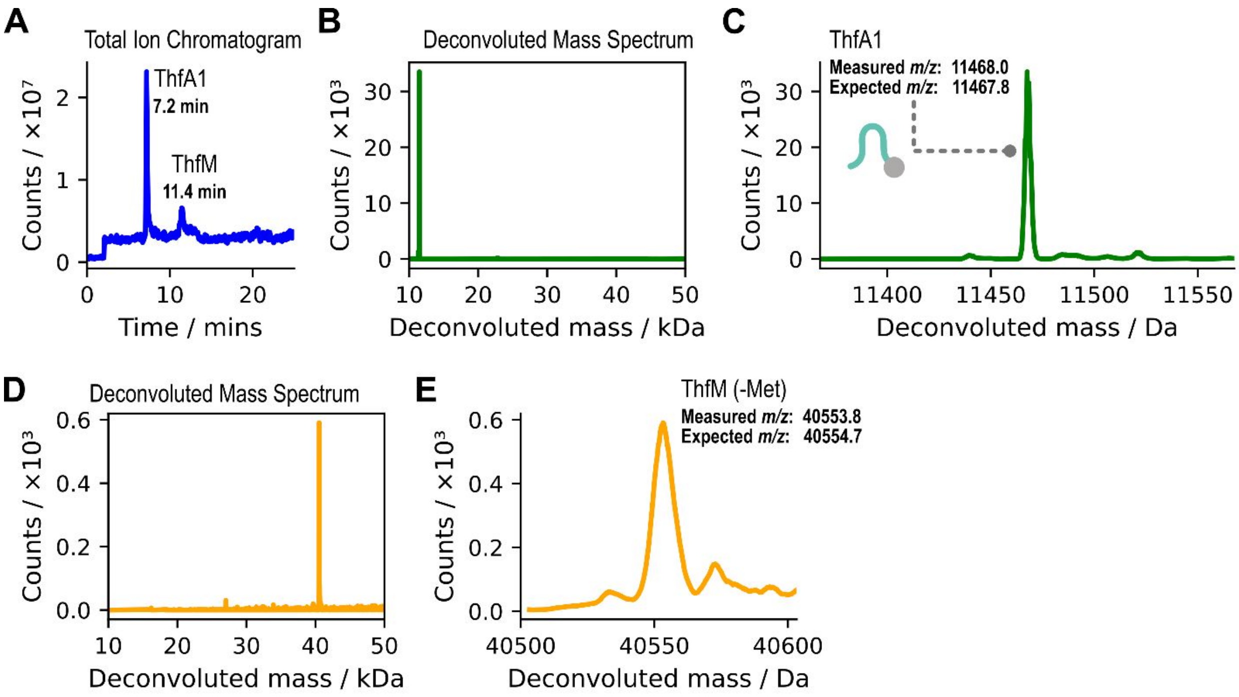

Figure S7. Mass spectrometry analysis of **ThfA1** expressed in *E. coli* BL21 (DE3) *ΔslyD* cells transformed with pTGJ007 and pTGJ056, purified from the cell pellet under denaturing conditions. A) The total ion chromatogram of purified protein, with the retention time of the peaks labelled. B) Deconvoluted mass spectrum of the major peak in the TIC. C) Magnified plot of the deconvoluted mass spectrum of the major peak showing the unmodified protein mass of **ThfA1**. The gray circle in the cartoon represents the leader peptide. D) Deconvoluted mass spectrum of the minor peak in the TIC. E) Magnified plot of the deconvoluted mass spectrum of the minor peak showing **ThfM** with loss of Met1. Despite the interaction of the precursor with the tailoring enzyme being strong enough to pull down some ThfM with the precursor during denaturing purification, no modified product was observed confirming ThfM only modifies the cyclized intermediate mThfA^{B} and not the linear precursor.

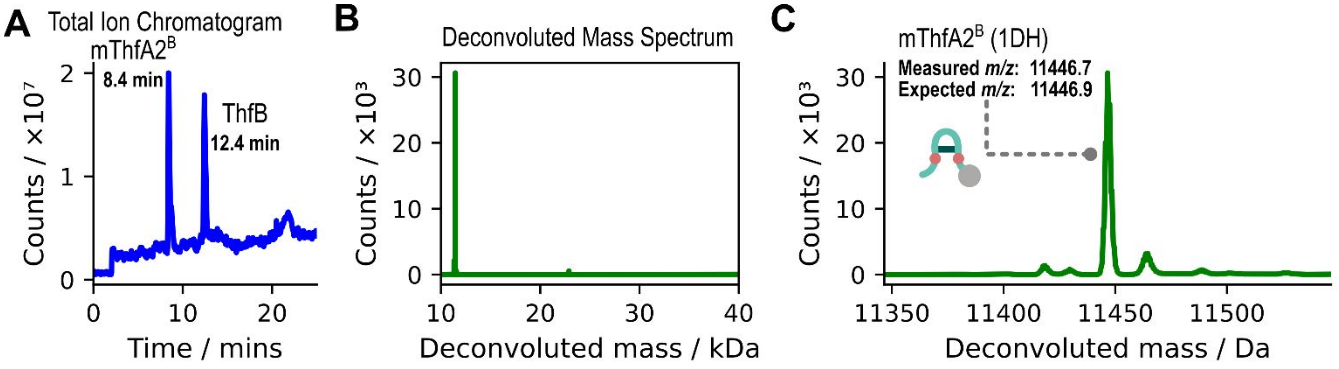

Figure S8. Mass spectrometry analysis of **mThfA2^B** expressed in *E. coli* BL21 (DE3) *ΔslyD* cells transformed with pTGJ066 and pBC262, purified from the cell pellet under denaturing conditions. A) The total ion chromatogram of purified protein, with the retention time of the peaks labelled. B) Deconvoluted mass spectrum of the major peak in the TIC. C) Magnified plot of the deconvoluted mass spectrum of the major peak showing **mThfA2^B** with one dehydration of the protein mass, indicative of the formation of one ester crosslink. The gray circle in the cartoon represents the leader peptide, and the two smaller red circles represent point substitutions.

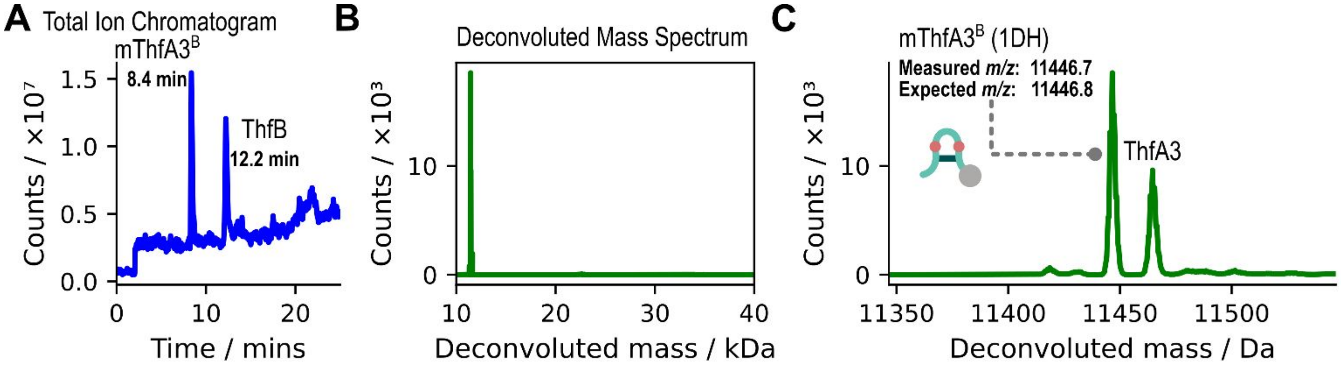

Figure S9. Mass spectrometry analysis of **mThfA3^B** expressed in *E. coli* BL21 (DE3) *ΔslyD* cells transformed with pTGJ067 and pBC262, purified from the cell pellet under denaturing conditions. A) The total ion chromatogram of purified protein, with the retention time of the peaks labelled. B) Deconvoluted mass spectrum of the major peak in the TIC. C) Magnified plot of the deconvoluted mass spectrum of the major peak showing **mThfA3^B** with one dehydration of the protein mass, indicative of the formation of one ester crosslink. The gray circle in the cartoon represents the leader peptide, and the two smaller red circles represent point substitutions.

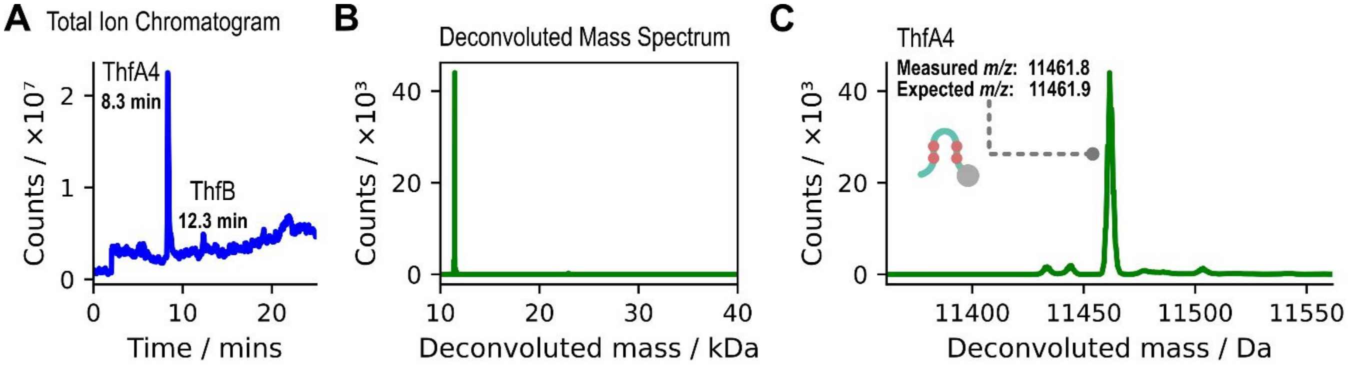

Figure S10. Mass spectrometry analysis of **ThfA4** expressed in *E. coli* BL21 (DE3) *ΔslyD* cells transformed with pTGJ068 and pBC262, purified from the cell pellet under denaturing conditions. A) The total ion chromatogram of purified protein, with the retention time of the peaks labelled. B) Deconvoluted mass spectrum of the major peak in the TIC. C) Magnified plot of the deconvoluted mass spectrum of the major peak showing **ThfA4** with no dehydration of the protein mass, indicative of no modification. The gray circle in the cartoon represents the leader peptide, and the four smaller red circles represent point substitutions.

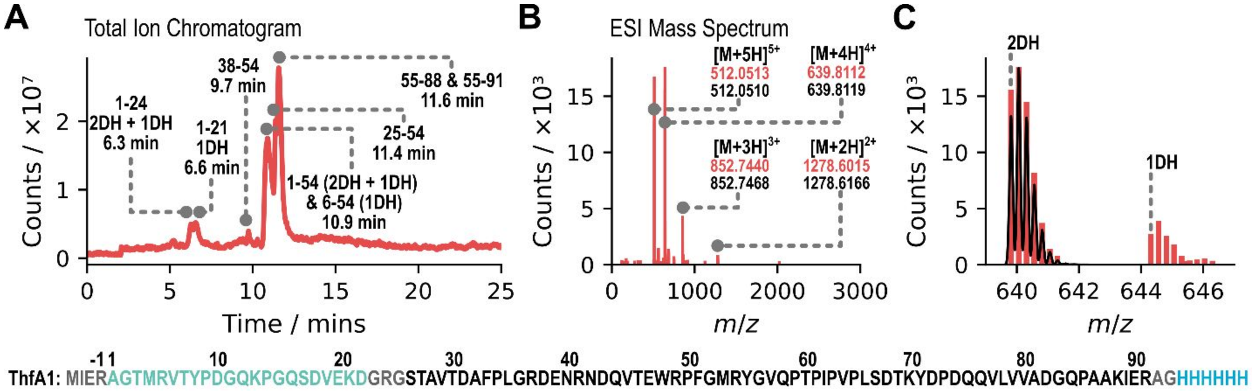

Figure S11. Mass spectrometry analysis of **mThfA1^{B}** trypsin digestion. A) The total ion chromatogram of digestion products following 15 min incubation at 37 °C, with the annotations and retention time of the peaks labelled. B) Electron spray ionization mass spectrum of the 1-24 (2DH + 1DH) peak in the TIC, where the top values (red) are the experimentally observed monoisotopic peak *m/z* value and the bottom values (black) are the theoretical *m/z* value. C) Magnified plot of the ESI mass spectrum showing the doubly and singly dehydrated 1-24 aa fragment $[M+4H]^{4+}$ ions with the simulated isotopic distribution shown as a black line for the doubly dehydrated fragment. The numbered peptide sequence for ThfA1 is shown for clarity.

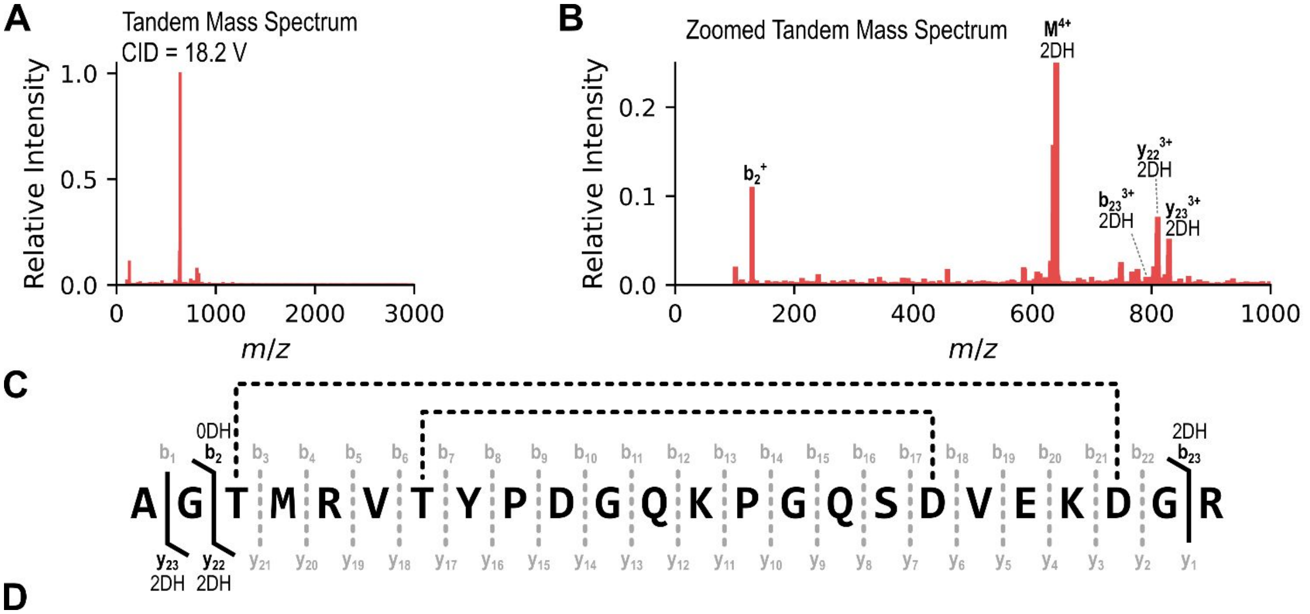

D

Ion	Measured *m/z*	Theoretical *m/z*	Error / ppm
**M (2DH)** $^{4+}$	639.8132	639.8119	-2.0
$b_2{}^{+}$	129.0638	129.0659	16.3
b_{23} **(2DH)** $^{3+}$	794.7014	794.7096	10.3
y_{23} **(2DH)** $^{3+}$	829.0664	829.0678	1.7
y_{22} **(2DH)** $^{3+}$	810.0526	810.0606	9.9

Figure S12. Tandem mass spectrometry analysis of the doubly dehydrated 1-24 aa **mThfA1**$^{\mathbf{B}}$ trypsin digestion product. A) Tandem mass spectrum of the doubly dehydrated trypsin product at retention time 6.2 min. B) Zoomed in tandem mass spectrum of the doubly dehydrated trypsin product, with fragment ions labelled. C) Structure of the bicyclic trypsin product with the fragment ions labelled. D) All identified ions from tandem mass spectrometry analysis with corresponding error values. The minimal fragmentation of the parent ion is characteristic of a cyclic structure, with fragment ions all being present at less than 10% of the parent ion intensity. Observation of doubly, singularly and no dehydrated y-ions is indicative of the positions of the crosslinks in the bicyclic structure.

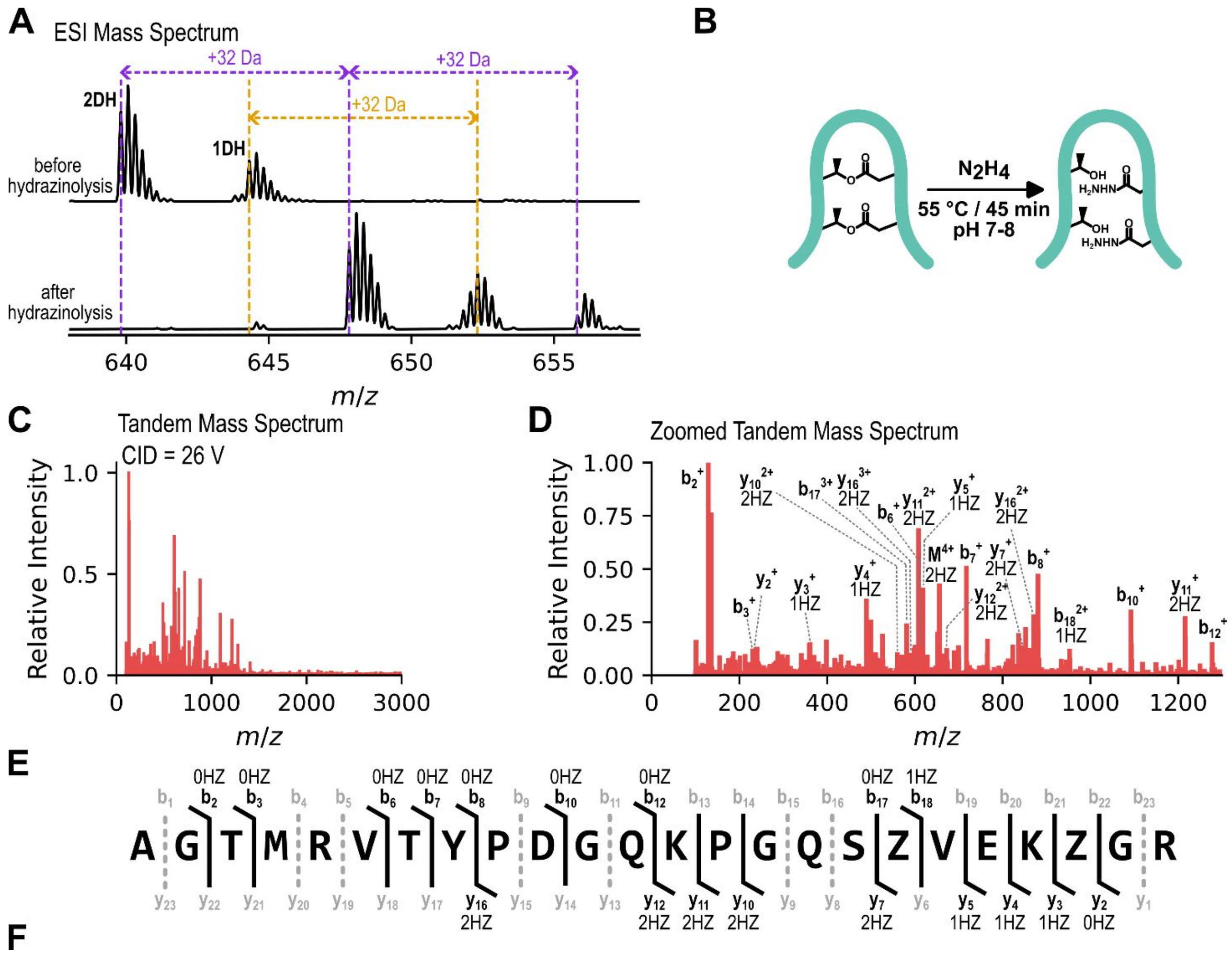

F

Ion	Measured *m/z*	Theoretical *m/z*	Error / ppm
**M (2HZ) $^{4+}$**	655.8275	655.8307	4.9
**b_2 $^{+}$**	129.0693	129.0659	-26.3
**b_3 $^{+}$**	230.1099	230.1135	15.6
**b_6 $^{+}$**	616.3140	616.3235	15.4
**b_7 $^{+}$**	717.3811	717.3712	-13.8
**b_8 $^{+}$**	880.4289	880.4345	6.4
**b_{10} $^{+}$**	1092.5078	1092.5143	5.9
**b_{12} $^{+}$**	1277.5925	1277.5943	1.4
**b_{17} $^{3+}$**	592.2882	592.2895	2.2
**b_{18} (1HZ) $^{2+}$**	952.4629	952.4576	-5.6
**y_2 $^{+}$**	232.1409	232.1404	-2.2
**y_3 (1HZ) $^{+}$**	361.1933	361.1942	2.5
**y_4 (1HZ) $^{+}$**	489.2882	489.2892	2.0
**y_5 (1HZ) $^{+}$**	618.3285	618.3318	5.3
**y_7 (2HZ) $^{+}$**	846.4445	846.4540	11.2
**y_{10} (2HZ) $^{2+}$**	559.7814	559.7867	9.5

**y_{11} (2HZ) $^{+}$**	1215.6136	1215.6189	4.4
**y_{11} (2HZ) $^{2+}$**	608.3091	608.3131	6.6
**y_{12} (2HZ) $^{2+}$**	672.3558	672.3606	7.1
**y_{16} (2HZ) $^{2+}$**	870.9376	870.9404	3.2
**y_{16} (2HZ) $^{3+}$**	580.9595	580.9627	5.5

Figure S13. Tandem mass spectrometry analysis following hydrazinolysis of HPLC purified doubly dehydrated 1-24 aa **mThfA1^{B}** trypsin digestion product. A) Stack of ESI mass spectra showing the doubly and singly dehydrated 1-24 aa fragment $[M+4H]^{4+}$ ions (top, before hydrazinolysis) and the products of hydrazinolysis with addition of one or two N_2H_4 (+32 Da) to the fragment mass (bottom, after hydrazinolysis). B) Schematic showing the hydrazinolysis of the ester crosslinks. C) Tandem mass spectrum of the hydrazinolysis product at retention time 5.6 min, with addition of two hydrazinyl groups splitting the ester crosslinks. D) Zoomed in tandem mass spectrum of the doubly tagged hydrazinolysis product, with fragment ions labelled. E) Structure of the linear hydrazinolysis product with two hydrazinyl tagged aspartic acid residues labelled Z, and the fragment ions labelled. F) All identified ions from tandem mass spectrometry analysis with corresponding error values. Observation of doubly, singularly and no hydrazinyl tagged ions is indicative of the Asp residues involved in the ester crosslinks of the bicyclic structure before hydrazinolysis.

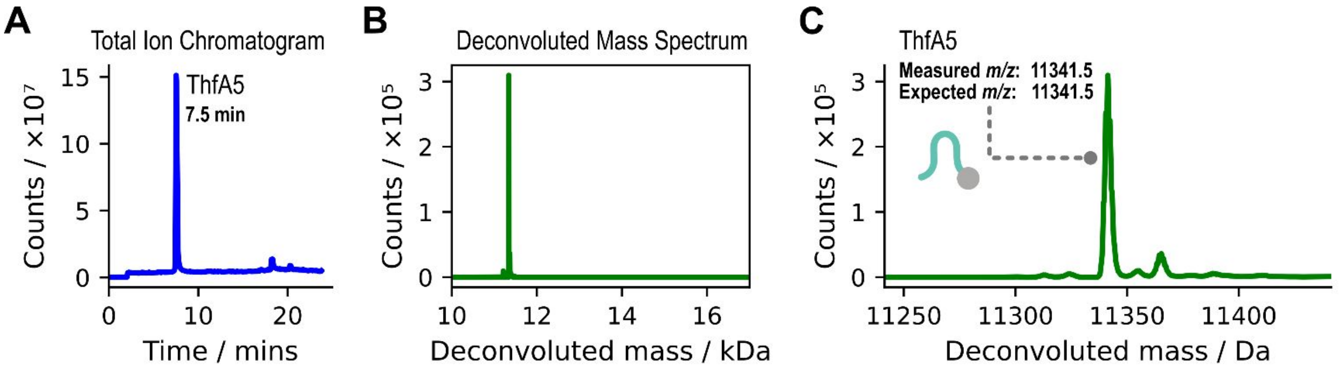

Figure S14. Mass spectrometry analysis of **ThfA5** expressed in *E. coli* BL21 (DE3) *ΔslyD* cells transformed with pTGJ005, purified from the cell pellet under denaturing conditions. A) The total ion chromatogram of purified protein, with the retention time of the peak labelled. B) Deconvoluted mass spectrum of the major peak in the TIC. C) Magnified plot of the deconvoluted mass spectrum of the major peak showing the unmodified protein mass of **ThfA5**. The gray circle in the cartoon represents the leader peptide.

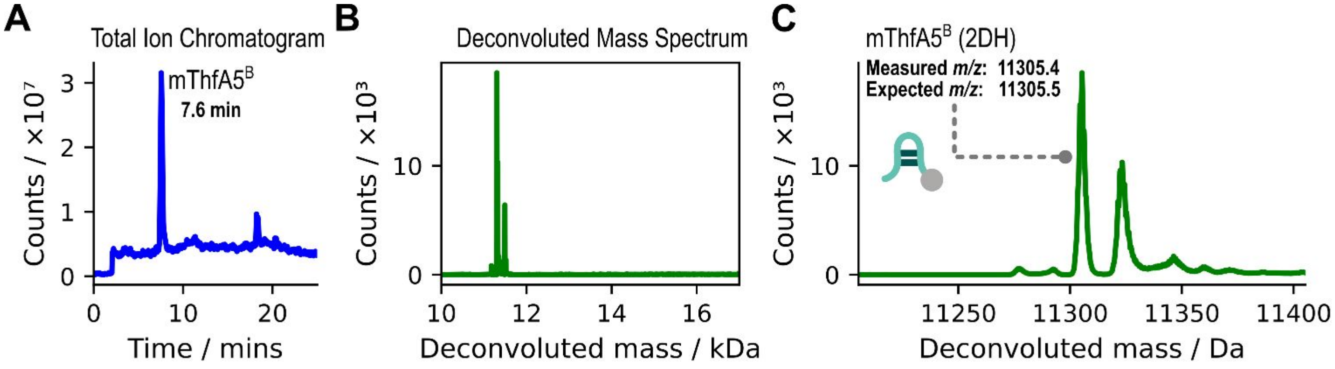

Figure S15. Mass spectrometry analysis of **mThfA5^{B}** expressed in *E. coli* BL21 (DE3) *ΔslyD* cells transformed with pTGJ005 and pBC262, purified from the cell pellet under denaturing conditions. A) The total ion chromatogram of purified protein, with the retention time of the peak labelled. B) Deconvoluted mass spectrum of the major peak in the TIC. C) Magnified plot of the deconvoluted mass spectrum of the major peak showing **mThfA5^{B}** with two-fold dehydration of the protein mass, indicative of the formation of two ester crosslinks. The gray circle in the cartoon represents the leader peptide.

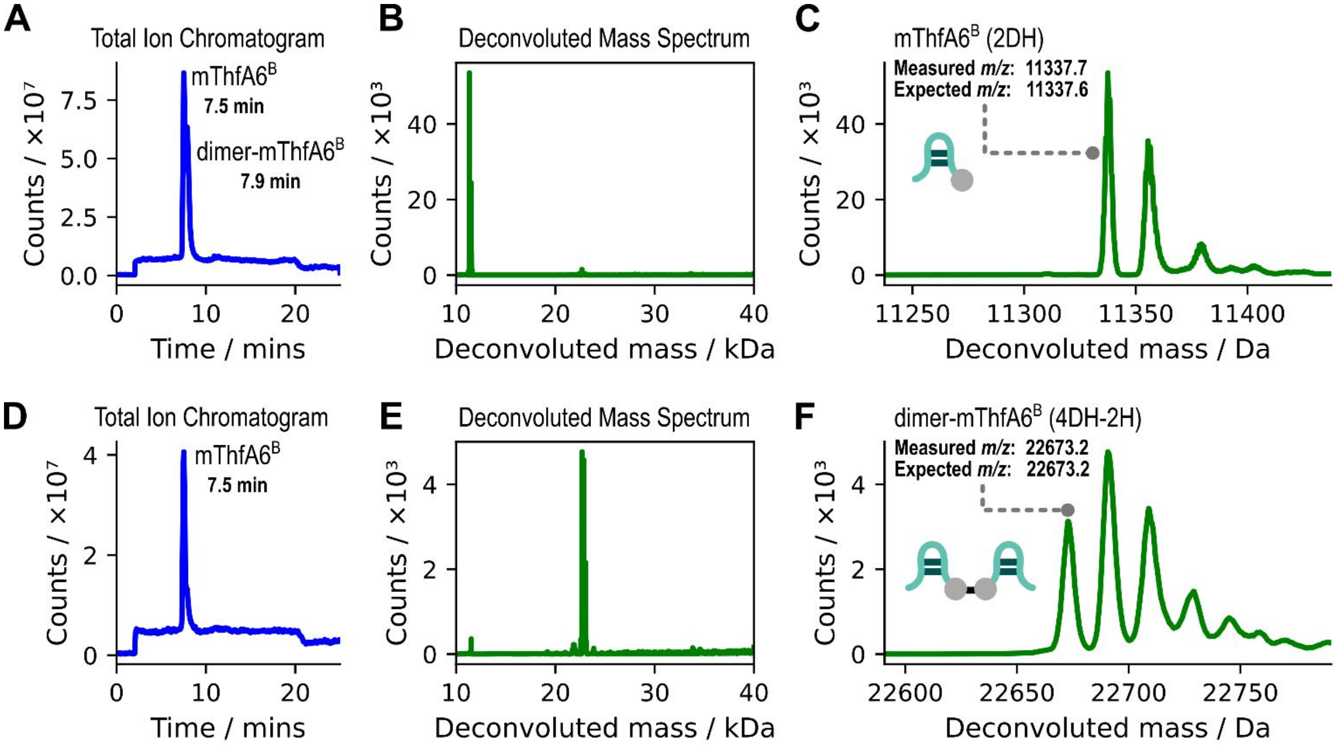

Figure S16. Mass spectrometry analysis of **mThfA6^{B}** expressed in *E. coli* BL21 (DE3) *ΔslyD* cells transformed with pTGJ004 and pBC262, purified from the cell pellet under denaturing conditions. A) The total ion chromatogram of purified protein without TCEP, with the retention time of the peaks labelled. B) Deconvoluted mass spectrum of the first peak in the TIC. C)

Magnified plot of the deconvoluted mass spectrum of the first peak showing **mThfA6^{B}** with up to two-fold dehydration of the protein mass, indicative of the formation of up to two ester crosslinks. The gray circle in the cartoon represents the leader peptide. D) The total ion chromatogram of purified protein after addition of TCEP, showing loss of the second peak from the TIC. E) Deconvoluted mass spectrum of the second peak in the TIC. F) Magnified plot of the deconvoluted mass spectrum of the second peak showing **dimer-mThfA6^{B}** with up to four-fold dehydration and loss of 2 hydrogens from the protein mass, indicative of the formation of a disulfide dimer with up to four ester crosslinks.

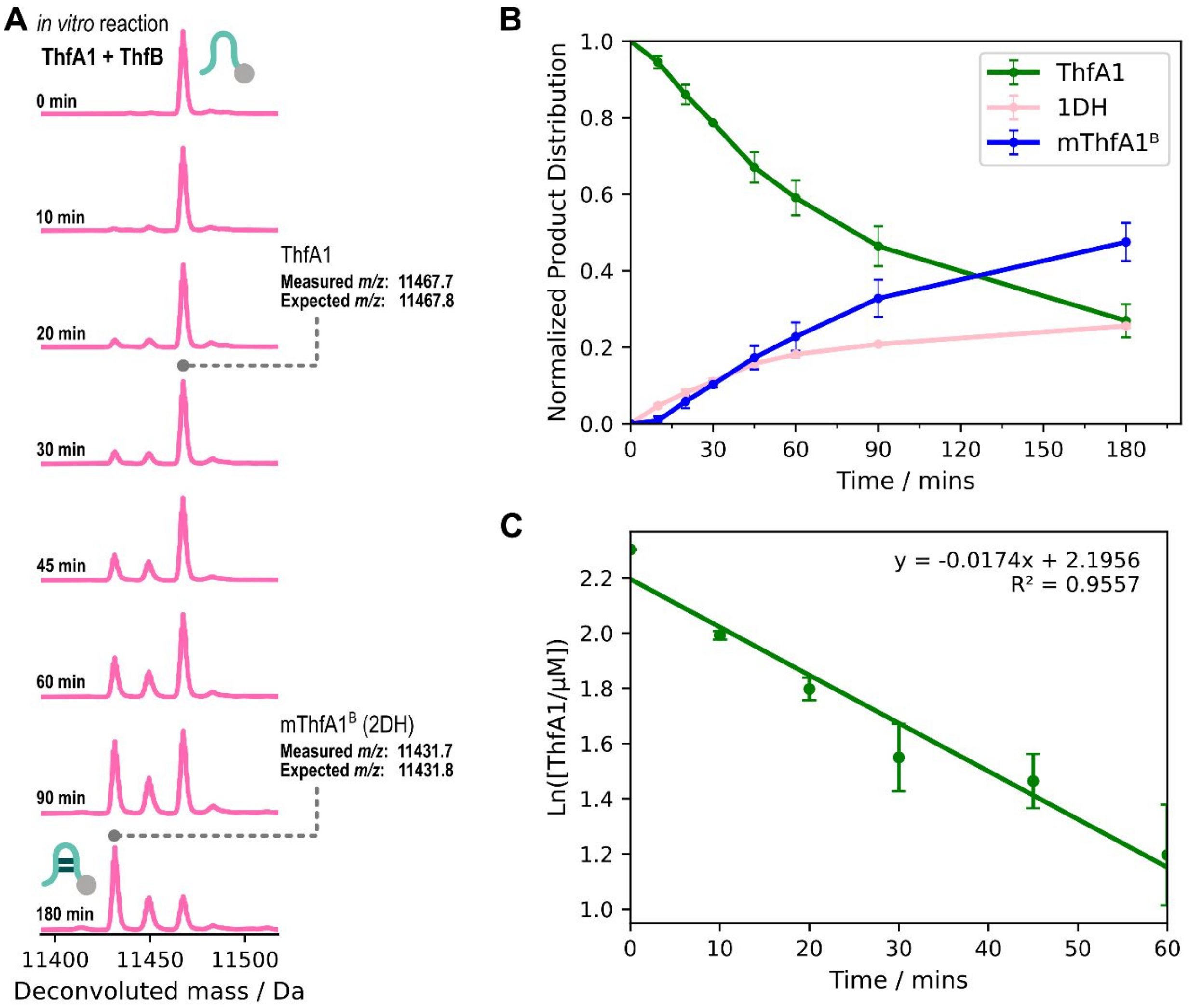

Figure S17. Kinetic analysis of the *in vitro* reaction of **ThfA1** (10 µM) with **ThfB** (1 µM) at 37 °C, monitored by LC-MS. A) Shows a representative stack of deconvoluted mass spectra, showing the product distribution for **mThfA1^{B}** over the course of a 3 h reaction. The gray circle in the cartoon represents the leader peptide. B) Changing product distribution over the course of reaction. C) The natural log of ThfA1 concentration plotted against time, fit by linear regression

to determine the initial rate of reaction, k_{ini} = 0.017±0.002 min^{-1}. Error bars represent the standard error of two replicates.

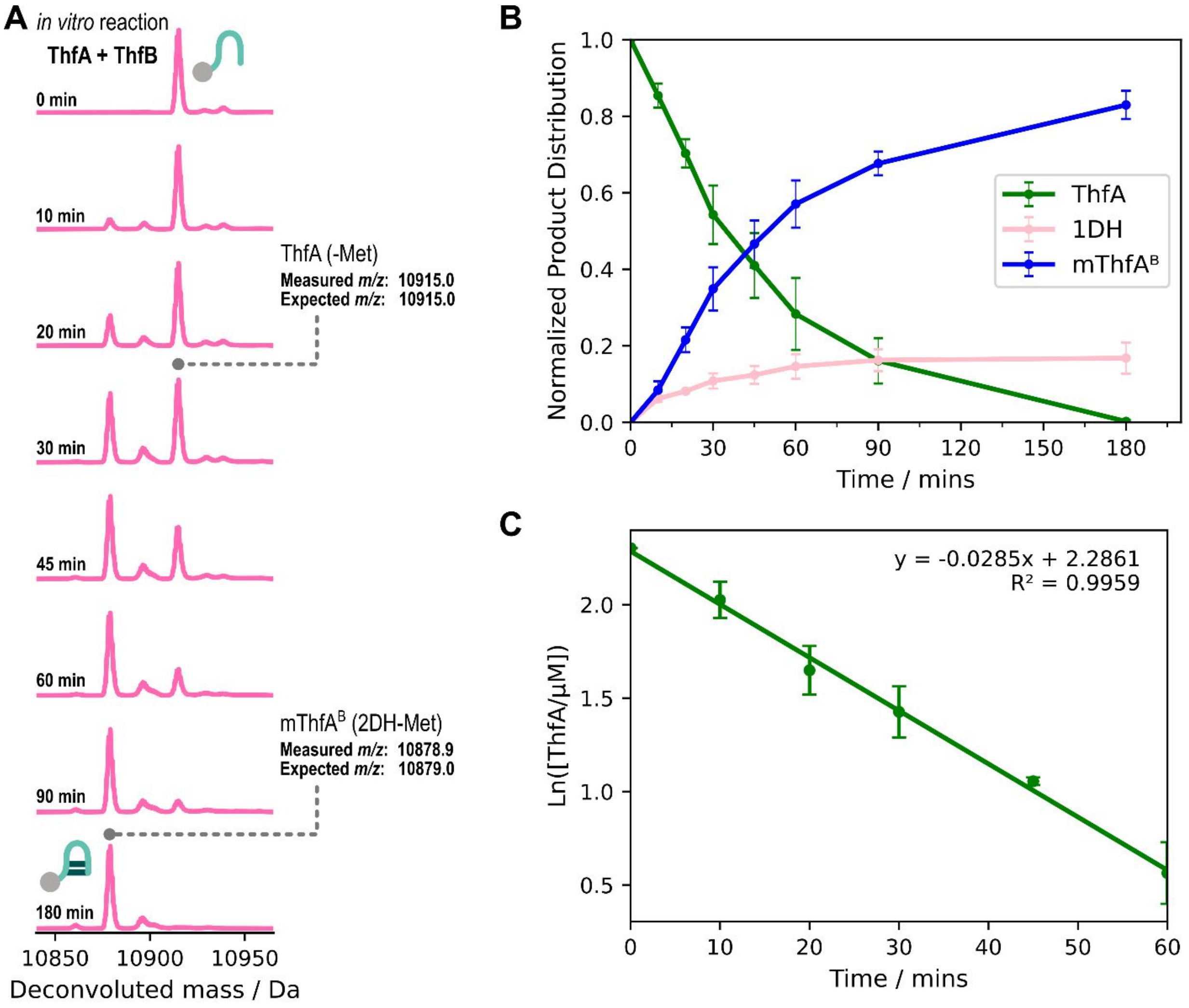

Figure S18. Kinetic analysis of the *in vitro* reaction of **ThfA** (10 μM) with **ThfB** (1 μM) at 37 °C, monitored by LC-MS. A) Shows a representative stack of deconvoluted mass spectra, showing the product distribution for **mThfA^{B}** over the course of a 3 h reaction. The gray circle in the cartoon represents the leader peptide. B) Changing product distribution over the course of reaction. C) The natural log of ThfA concentration plotted against time, fit by linear regression to determine the initial rate of reaction, k_{ini} = 0.029±0.002 min^{-1}. Error bars represent the standard error of two replicates.

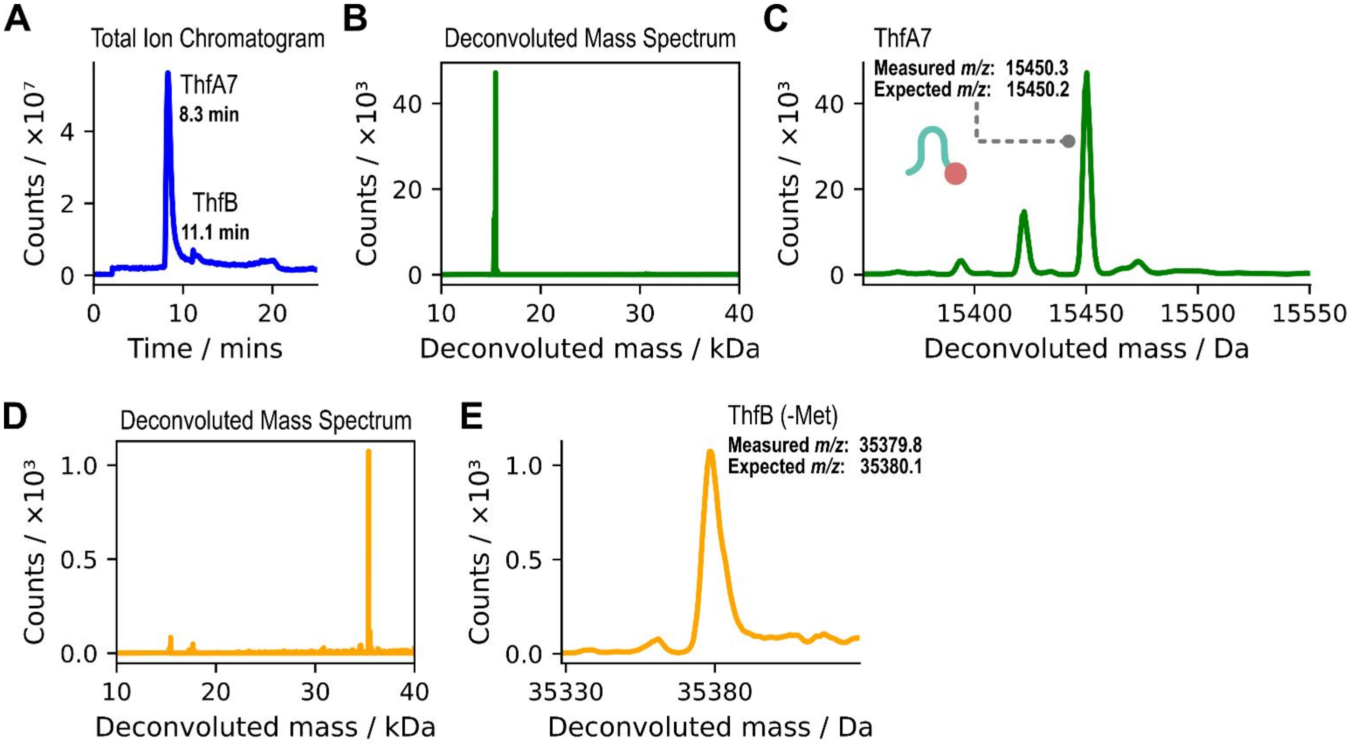

Figure S19. Mass spectrometry analysis of **ThfA7** expressed in *E. coli* BL21 (DE3) *ΔslyD* cells transformed with pTGJ027 and pBC262, purified from the cell pellet under native conditions. A) The total ion chromatogram of purified protein, with the retention time of the peaks labelled. B) Deconvoluted mass spectrum of the major peak in the TIC. C) Magnified plot of the deconvoluted mass spectrum of the major peak showing the unmodified protein mass of **ThfA7**. The red circle in the cartoon represents SUMO. D) Deconvoluted mass spectrum of the minor peak in the TIC. E) Magnified plot of the deconvoluted mass spectrum of the minor peak showing **ThfB** with loss of Met1. Purifying **ThfA7** without the native leader sequence under native conditions pulled down some ThfB, showing that ThfB interacts with the core sequence but with a diminished affinity compared to the leader sequence. Despite the interaction of the core sequence with ThfB, no modification of the precursor was observed confirming ThfB is truly a leader-dependent tailoring enzyme.

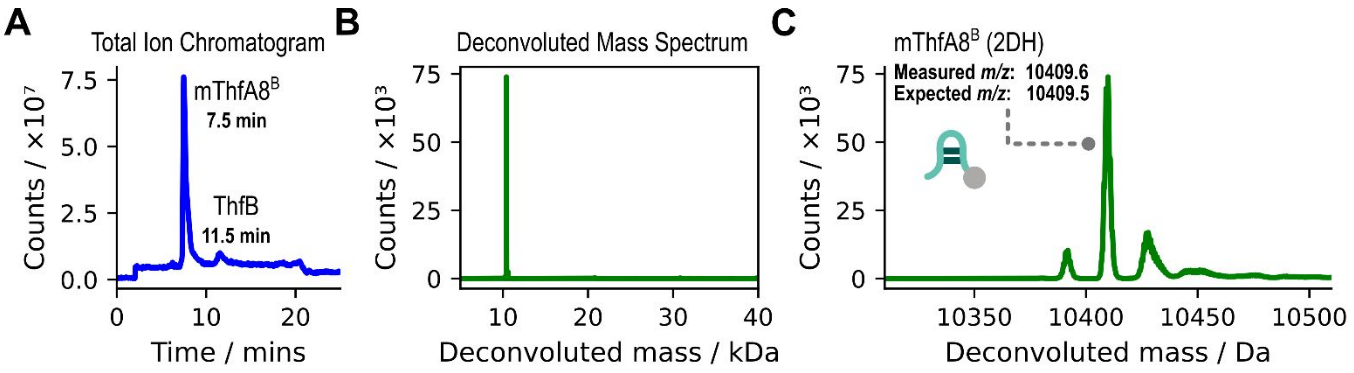

Figure S20. Mass spectrometry analysis of **mThfA8^{B}** expressed in *E. coli* BL21 (DE3) *ΔslyD* cells transformed with pTGJ037 and pBC262, purified from the cell pellet under denaturing conditions. A) The total ion chromatogram of purified protein, with the retention time of the peaks labelled. B) Deconvoluted mass spectrum of the major peak in the TIC. C) Magnified plot of the deconvoluted mass spectrum of the major peak showing **mThfA8^{B}** with two-fold dehydration of the protein mass, indicative of the formation of two ester crosslinks. The gray circle in the cartoon represents the leader peptide.

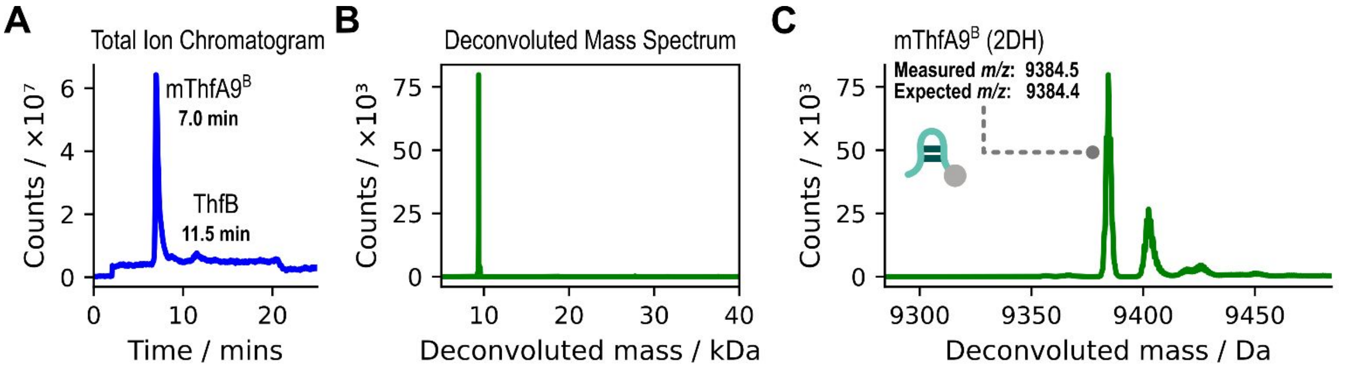

Figure S21. Mass spectrometry analysis of **mThfA9^{B}** expressed in *E. coli* BL21 (DE3) *ΔslyD* cells transformed with pTGJ038 and pBC262, purified from the cell pellet under denaturing conditions. A) The total ion chromatogram of purified protein, with the retention time of the peaks labelled. B) Deconvoluted mass spectrum of the major peak in the TIC. C) Magnified plot of the deconvoluted mass spectrum of the major peak showing **mThfA9^{B}** with two-fold dehydration of the protein mass, indicative of the formation of two ester crosslinks. The gray circle in the cartoon represents the leader peptide.

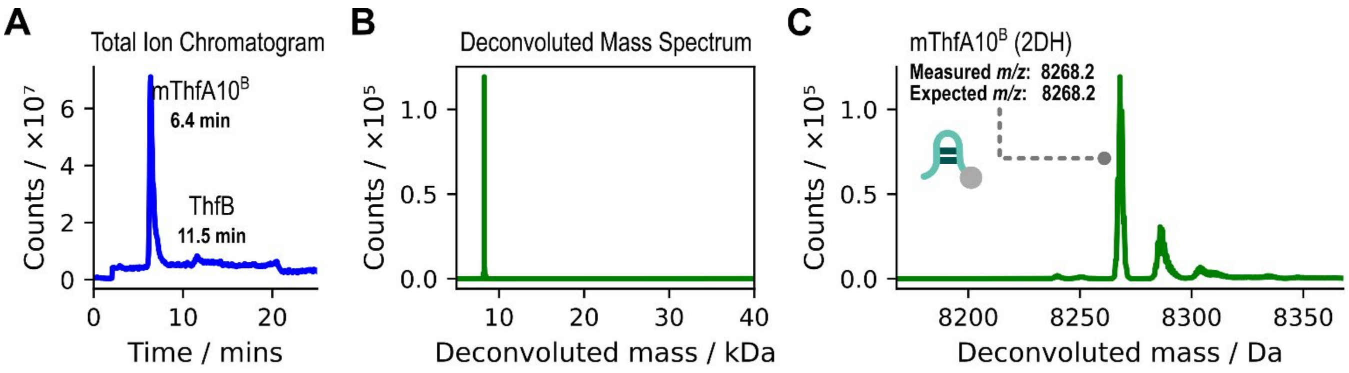

Figure S22. Mass spectrometry analysis of **mThfA10$^{\mathbf{B}}$** expressed in *E. coli* BL21 (DE3) *ΔslyD* cells transformed with pTGJ039 and pBC262, purified from the cell pellet under denaturing conditions. A) The total ion chromatogram of purified protein, with the retention time of the peaks labelled. B) Deconvoluted mass spectrum of the major peak in the TIC. C) Magnified plot of the deconvoluted mass spectrum of the major peak showing **mThfA10$^{\mathbf{B}}$** with two-fold dehydration of the protein mass, indicative of the formation of two ester crosslinks. The gray circle in the cartoon represents the leader peptide.

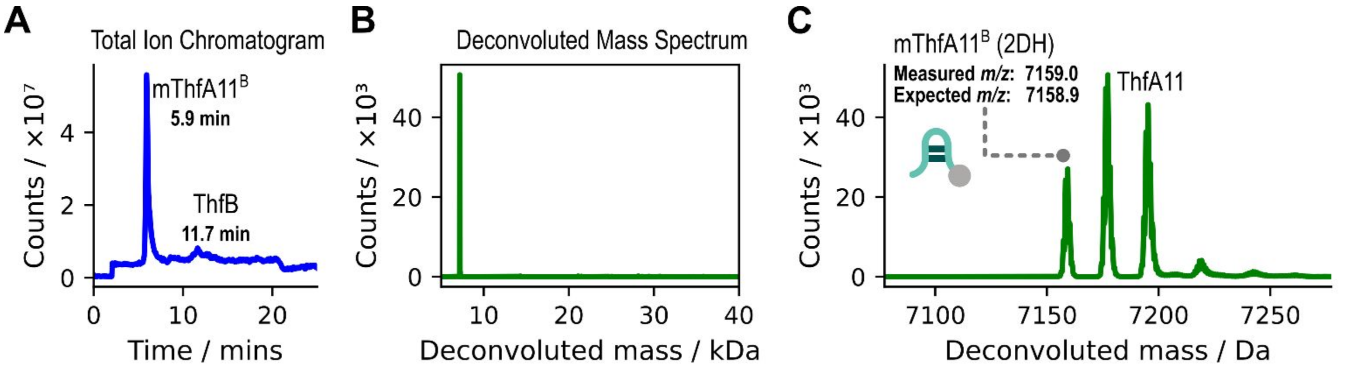

Figure S23. Mass spectrometry analysis of **mThfA11$^{\mathbf{B}}$** expressed in *E. coli* BL21 (DE3) *ΔslyD* cells transformed with pTGJ040 and pBC262, purified from the cell pellet under denaturing conditions. A) The total ion chromatogram of purified protein, with the retention time of the peaks labelled. B) Deconvoluted mass spectrum of the major peak in the TIC. C) Magnified plot of the deconvoluted mass spectrum of the major peak showing **mThfA11$^{\mathbf{B}}$** with up to two-fold dehydration of the protein mass, indicative of the formation of up to two ester crosslinks. The gray circle in the cartoon represents the leader peptide.

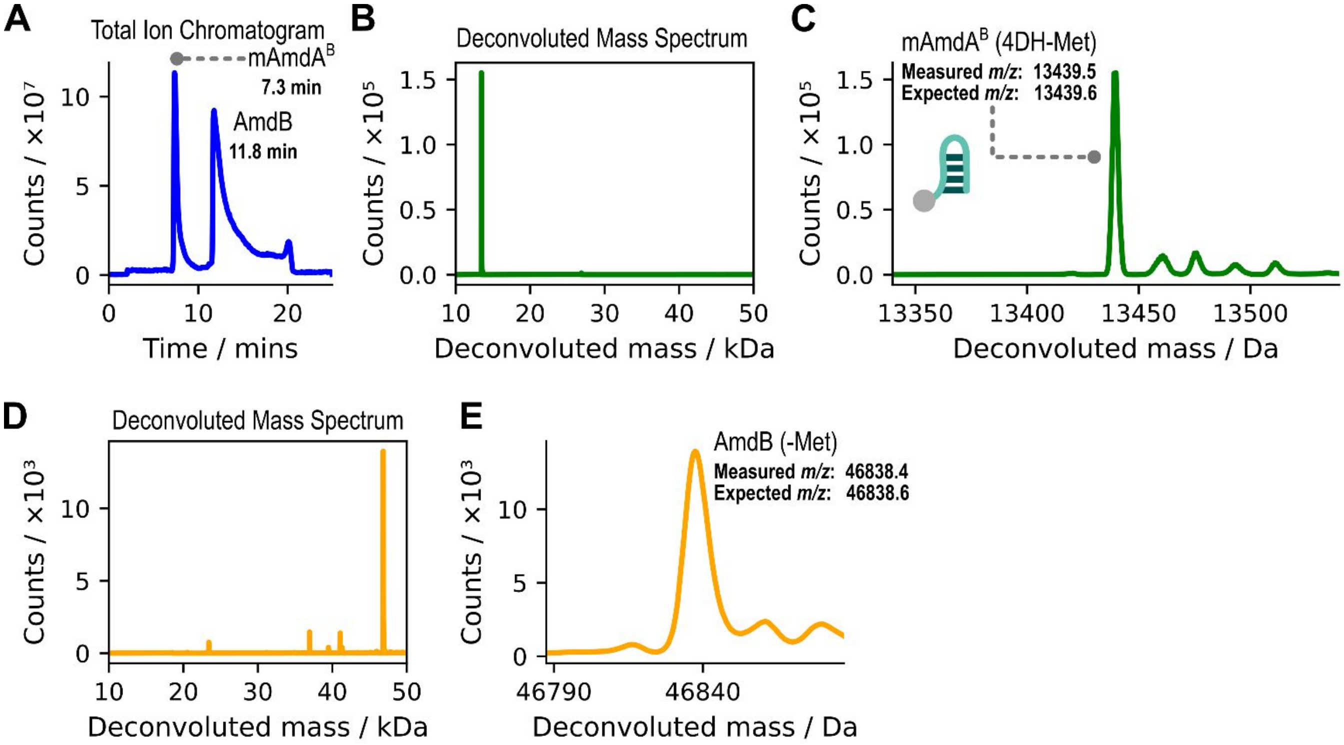

Figure S24. Mass spectrometry analysis of **mAmdA^B** expressed in *E. coli* BL21 (DE3) *ΔslyD* cells transformed with pBC045, purified from the cell pellet under native conditions. A) The total ion chromatogram of purified protein, with the retention time of the peaks labelled. B) Deconvoluted mass spectrum of the first peak in the TIC. C) Magnified plot of the deconvoluted mass spectrum of the first peak showing **mAmdA^B** with loss of Met1 and four-fold dehydration of the protein mass, indicative of the formation of four ester crosslinks. The gray circle in the cartoon represents the leader peptide. D) Deconvoluted mass spectrum of the second peak in the TIC. E) Magnified plot of the deconvoluted mass spectrum of the second peak showing **AmdB** with loss of Met1.

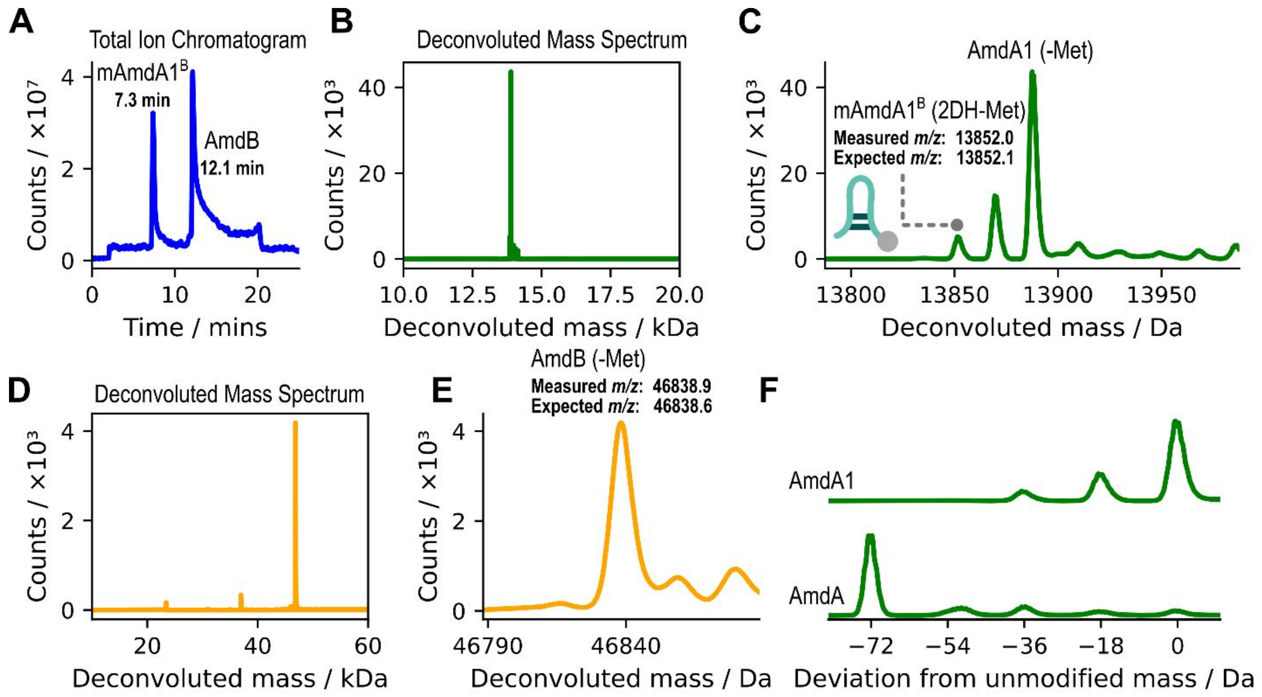

Figure S25. Mass spectrometry analysis of **mAmdA1^{B}** expressed for 2 days in *E. coli* BL21 (DE3) *ΔslyD* cells transformed with pTGJ063 and pTGJ064, purified from the cell pellet under native conditions. A) The total ion chromatogram of purified protein, with the retention time of the peaks labelled. B) Deconvoluted mass spectrum of the first peak in the TIC. C) Magnified plot of the deconvoluted mass spectrum of the first peak showing **mAmdA1^{B}** with loss of Met1 and up to two-fold dehydration of the protein mass, indicative of the formation of up to two ester crosslinks. The gray circle in the cartoon represents the leader peptide. D) Deconvoluted mass spectrum of the second peak in the TIC. E) Magnified plot of the deconvoluted mass spectrum of the second peak showing **AmdB** with loss of Met1. F) Stack of the product distribution for AmdB modification of AmdA1 and AmdA, shown as mass deviation from unmodified proteins.

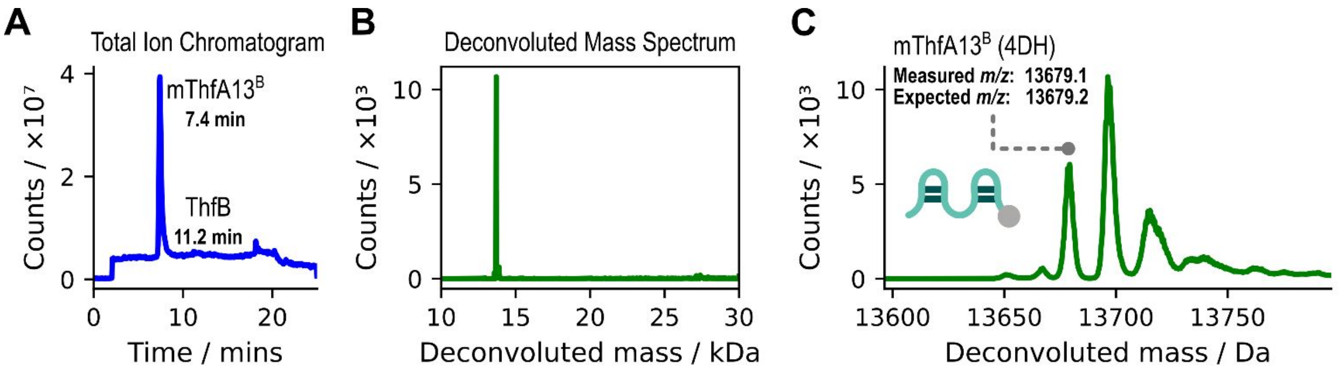

Figure S26. Mass spectrometry analysis of **mThfA13^{B}** expressed in *E. coli* BL21 (DE3) *ΔslyD* cells transformed with pTGJ011 and pBC262, purified from the cell pellet under denaturing conditions. A) The total ion chromatogram of purified protein in the presence of TCEP, with the retention time of the peaks labelled. B) Deconvoluted mass spectrum of the major peak in the TIC. C) Magnified plot of the deconvoluted mass spectrum of the major peak showing **mThfA13^{B}** with up to four-fold dehydration of the protein mass, indicative of the formation of up to four ester crosslinks. The gray circle in the cartoon represents the leader peptide.

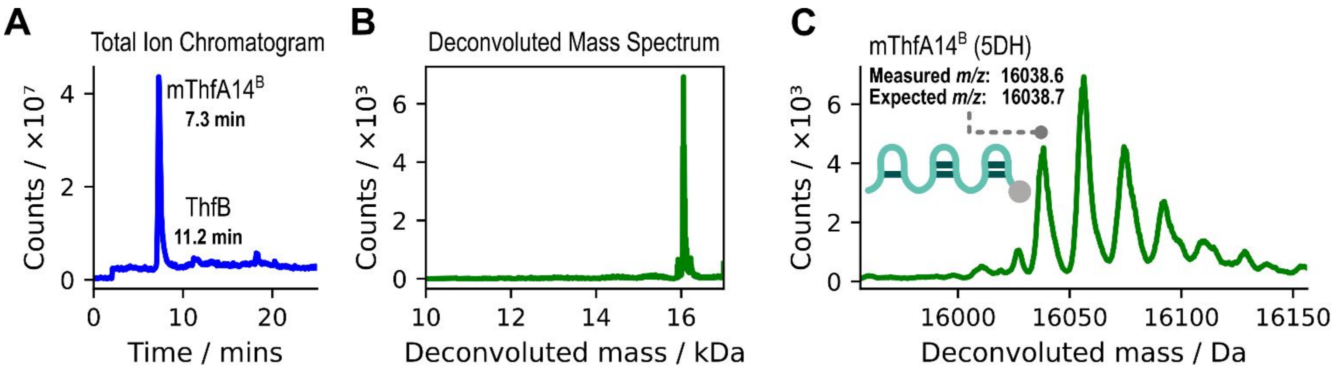

Figure S27. Mass spectrometry analysis of **mThfA14^{B}** expressed in *E. coli* BL21 (DE3) *ΔslyD* cells transformed with pTGJ006 and pBC262, purified from the cell pellet under denaturing conditions. A) The total ion chromatogram of purified protein in the presence of TCEP, with the retention time of the peaks labelled. B) Deconvoluted mass spectrum of the major peak in the TIC. C) Magnified plot of the deconvoluted mass spectrum of the major peak showing **mThfA14^{B}** with up to five-fold dehydration of the protein mass, indicative of the formation of up to five ester crosslinks. The gray circle in the cartoon represents the leader peptide.

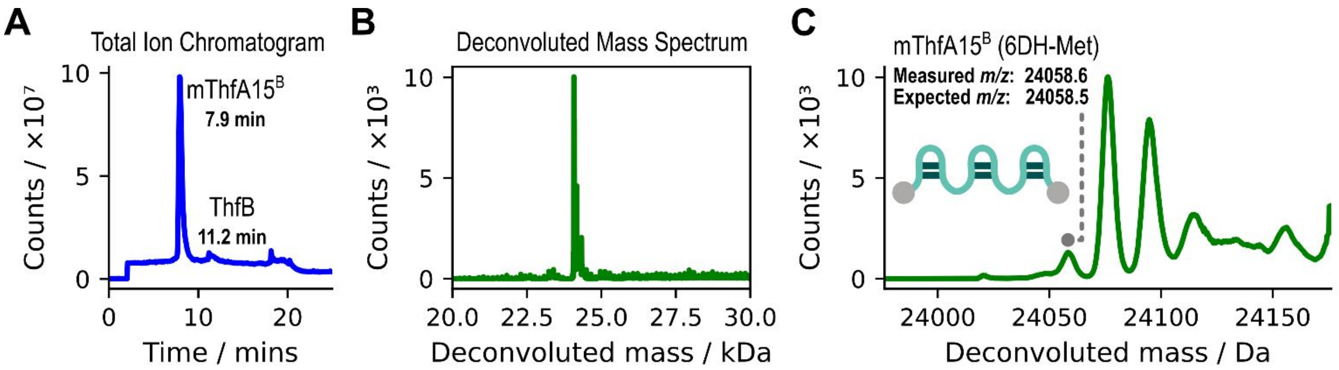

Figure S28. Mass spectrometry analysis of **mThfA15^{B}** expressed in *E. coli* BL21 (DE3) *ΔslyD* cells transformed with pTGJ008 and pBC262, purified from the cell pellet under denaturing conditions. A) The total ion chromatogram of purified protein in the presence of TCEP, with the retention time of the peaks labelled. B) Deconvoluted mass spectrum of the major peak in the TIC. C) Magnified plot of the deconvoluted mass spectrum of the major peak showing **mThfA15^{B}** with loss of Met1 and up to six-fold dehydration of the protein mass, indicative of the formation of up to six ester crosslinks. The gray circle in the cartoon represents the leader peptide.

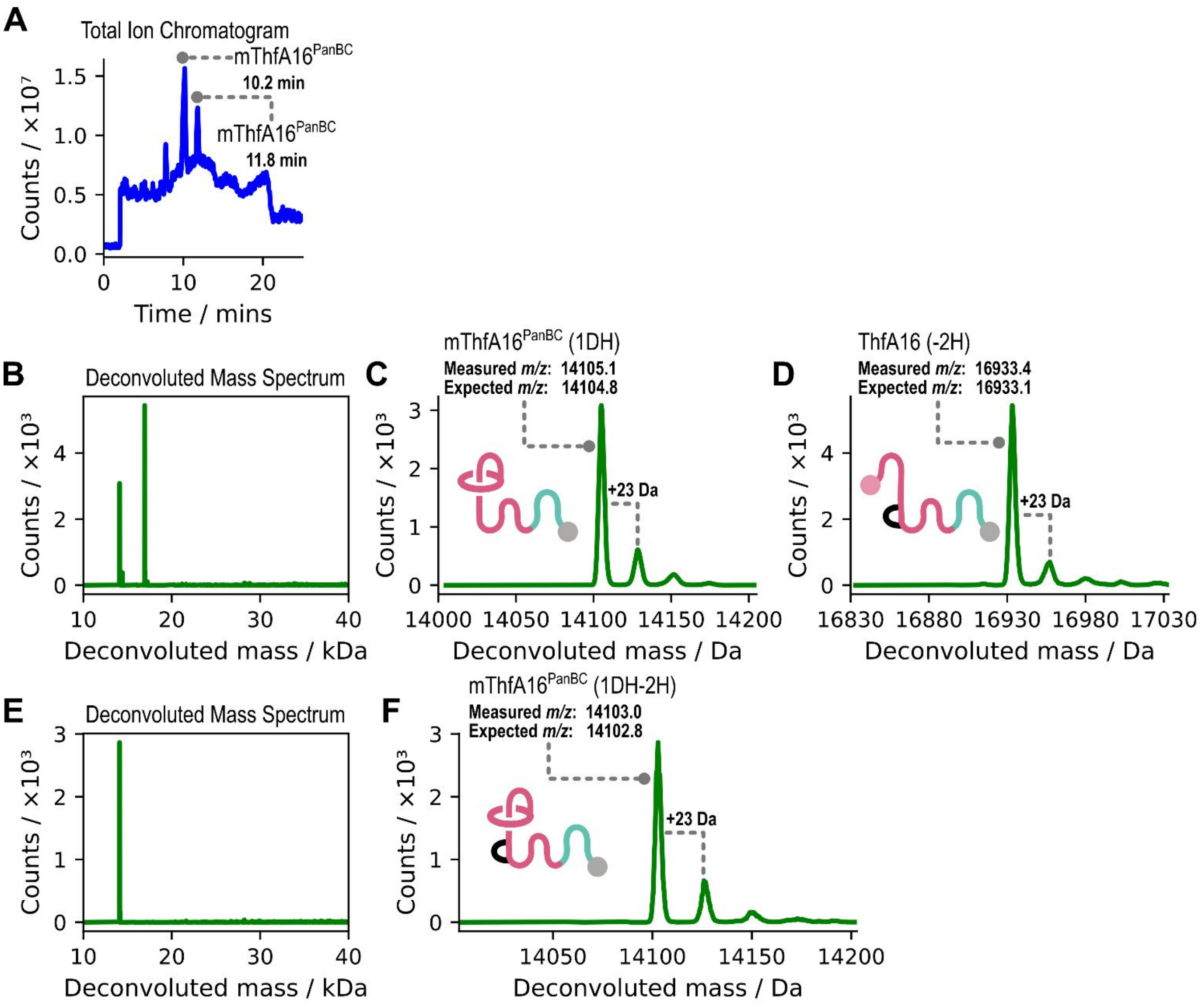

Figure S29. Mass spectrometry analysis of **mThfA16^{PanBC}** expressed in *E. coli* BL21 (DE3) *ΔslyD* cells transformed with pTGJ010, purified from the cell pellet under denaturing conditions. A) The total ion chromatogram of purified protein, with the retention time of the peaks labelled. B) Deconvoluted mass spectrum of the major peak in the TIC. C) Magnified plot of the deconvoluted mass spectrum of the major peak showing **mThfA16^{PanBC}** with one dehydration of the protein mass, indicative of the formation of the lasso peptide isopeptide bond. The pink circle represents the pandonodin leader peptide and the gray circle in the cartoon represents the ThfA leader peptide. D) Magnified plot of the deconvoluted mass spectrum of the major peak showing the unmodified protein mass of **ThfA16** with loss of 2 hydrogens, indicative of the formation of a disulfide bond. E) Deconvoluted mass spectrum of the minor peak in the TIC. F) Magnified plot of the deconvoluted mass spectrum of the minor peak showing **mThfA16^{PanBC}** with loss of 2 hydrogens and one dehydration of the protein mass, indicative of the formation of a disulfide bond and the lasso peptide isopeptide bond.

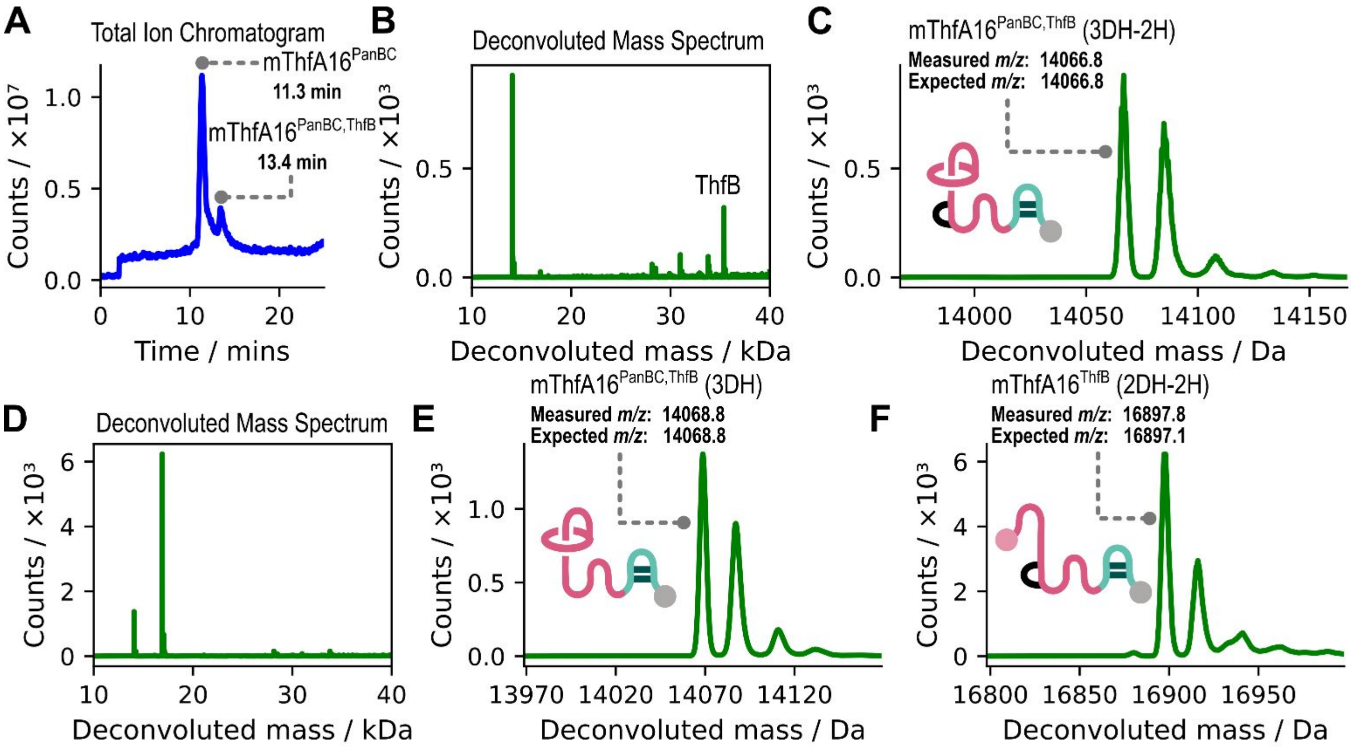

Figure S30. Mass spectrometry analysis of **mThfA16^{PanBC,ThfB}** expressed in *E. coli* BL21 (DE3) *ΔslyD* cells transformed with pTGJ010 and pBC262, purified from the cell pellet under denaturing conditions. A) The total ion chromatogram of purified protein, with the retention time of the peaks labelled. B) Deconvoluted mass spectrum of the minor peak in the TIC. C) Magnified plot of the deconvoluted mass spectrum of the minor peak showing **mThfA16^{PanBC,ThfB}** with loss of 2 hydrogens and up to three dehydrations of the protein mass, indicative of the formation of a disulfide bond, the lasso peptide isopeptide bond and two ester crosslinks. D) Deconvoluted mass spectrum of the major peak in the TIC. E) Magnified plot of the deconvoluted mass spectrum of the major peak showing **mThfA16^{PanBC,ThfB}** with up to three dehydrations of the protein mass, indicative of the formation of the lasso peptide isopeptide bond and two ester crosslinks. F) Magnified plot of the deconvoluted mass spectrum of the major peak showing **mThfA16^{ThfB}** with loss of 2 hydrogens and up to two dehydrations of the protein mass, indicative of the formation of a disulfide bond and up to two ester crosslinks. The pink circle represents the pandonodin leader peptide and the gray circle in the cartoon represents the ThfA leader peptide.

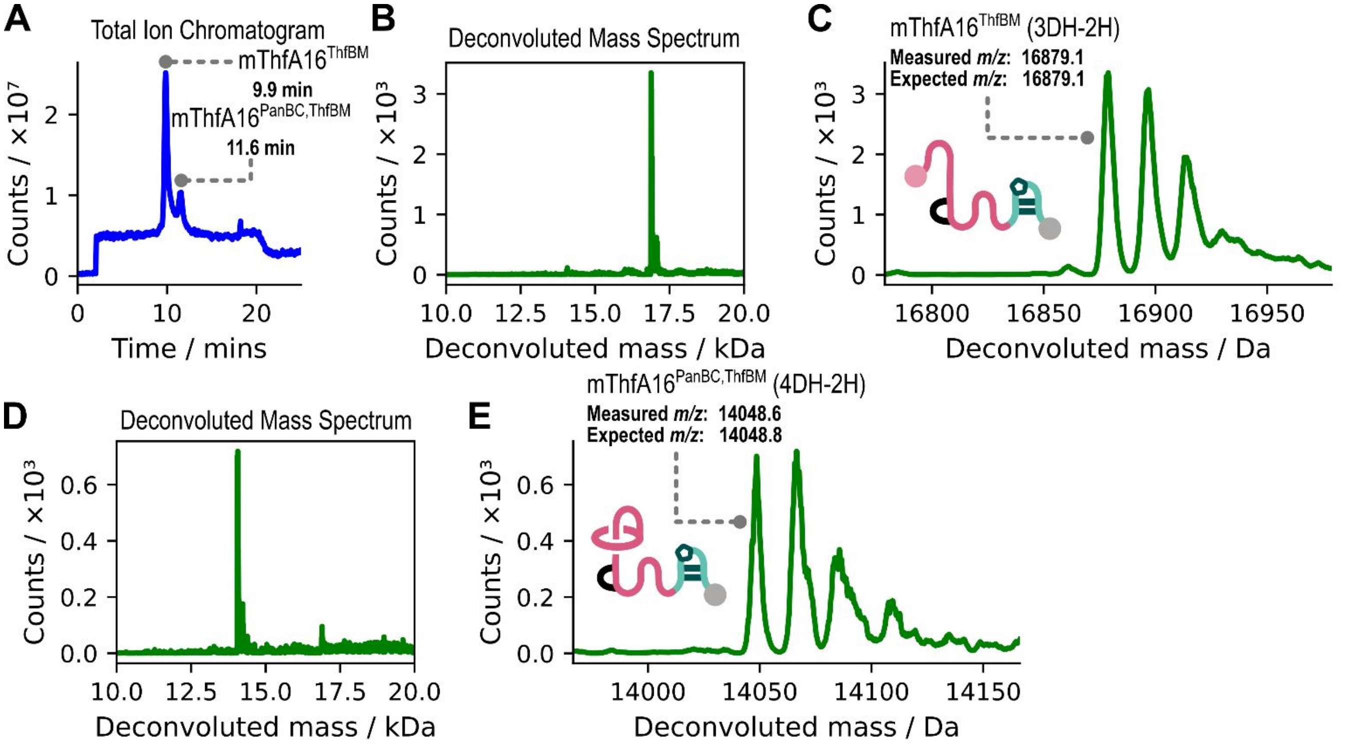

Figure S31. Mass spectrometry analysis of **mThfA16^{PanBC,ThfBM}** expressed in *E. coli* BL21 (DE3) *ΔslyD* cells transformed with pTGJ010 and pTGJ009, purified from the cell pellet under denaturing conditions. A) The total ion chromatogram of purified protein, with the retention time of the peaks labelled. B) Deconvoluted mass spectrum of the major peak in the TIC. C) Magnified plot of the deconvoluted mass spectrum of the major peak showing **mThfA16^{ThfBM}** with loss of 2 hydrogens and up to three dehydrations of the protein mass, indicative of the formation of a disulfide bond, two ester crosslinks and the aspartimidylation. The pink circle represents the pandonodin leader peptide and the gray circle in the cartoon represents the ThfA leader peptide. D) Deconvoluted mass spectrum of the minor peak in the TIC. E) Magnified plot of the deconvoluted mass spectrum of the minor peak showing **mThfA16^{PanBC,ThfBM}** with loss of 2 hydrogens and up to four dehydrations of the protein mass, indicative of the formation of a disulfide bond, the lasso peptide isopeptide bond, two ester crosslinks and the aspartimidylation.

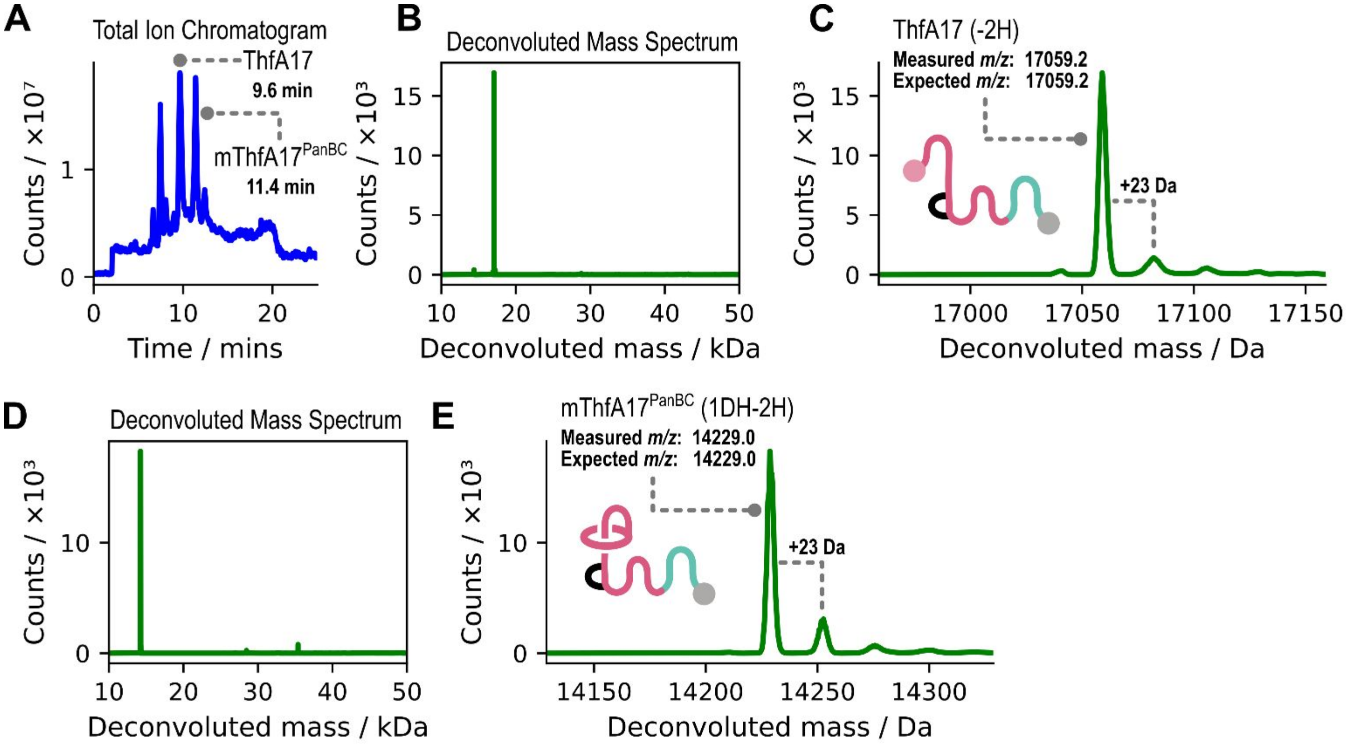

Figure S32. Mass spectrometry analysis of **mThfA17^{PanBC}** expressed in *E. coli* BL21 (DE3) *ΔslyD* cells transformed with pTGJ031, purified from the cell pellet under native conditions. A) The total ion chromatogram of purified protein, with the retention time of the peaks labelled. B) Deconvoluted mass spectrum of the major peak in the TIC. C) Magnified plot of the deconvoluted mass spectrum of the major peak showing the unmodified protein mass of **ThfA17** with loss of 2 hydrogens, indicative of the formation of a disulfide bond. The pink circle represents the pandonodin leader peptide and the gray circle in the cartoon represents the ThfA leader peptide. D) Deconvoluted mass spectrum of the minor peak in the TIC. E) Magnified plot of the deconvoluted mass spectrum of the minor peak showing **mThfA17^{PanBC}** with loss of 2 hydrogens and one dehydration of the protein mass, indicative of the formation of a disulfide bond and the lasso peptide isopeptide bond.

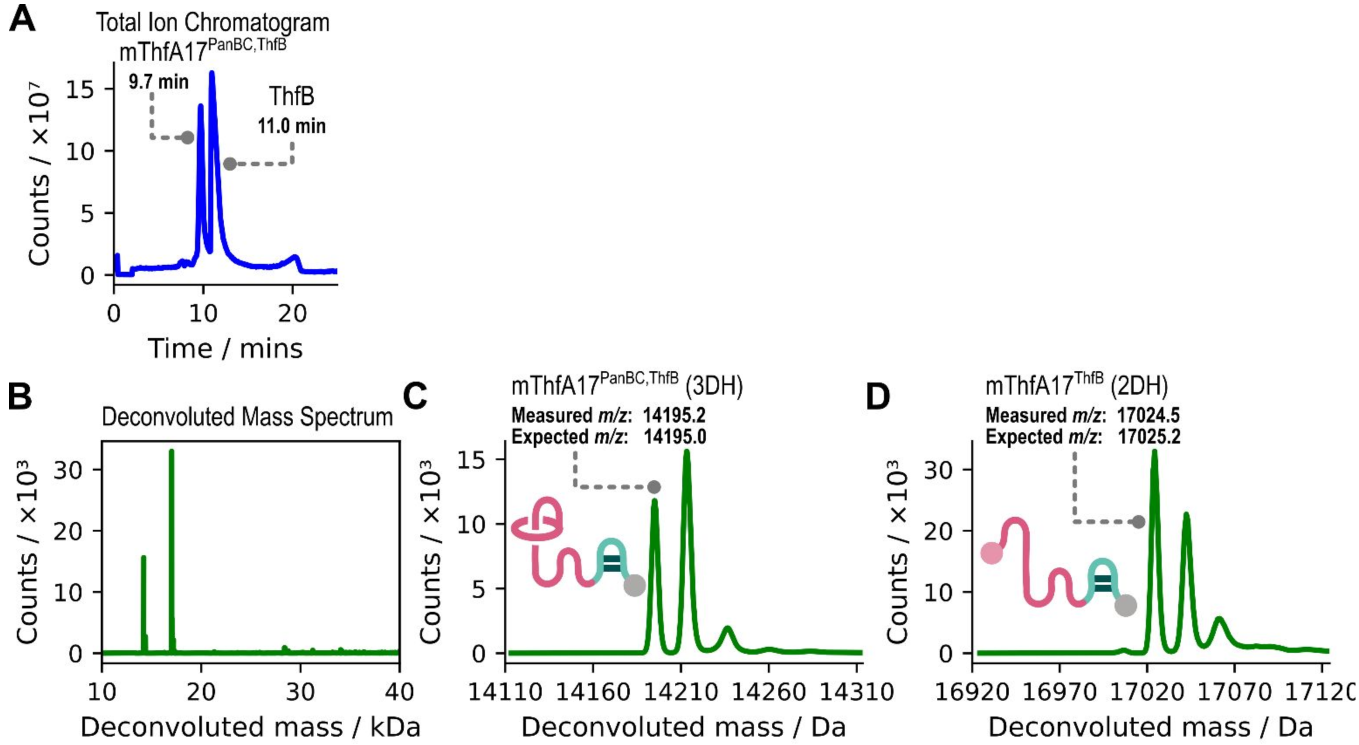

Figure S33. Mass spectrometry analysis of **mThfA17^{PanBC,ThfB}** expressed in *E. coli* BL21 (DE3) *ΔslyD* cells transformed with pTGJ031 and pBC262, purified from the cell pellet under native conditions. A) The total ion chromatogram of purified protein, with the retention time of the peaks labelled. B) Deconvoluted mass spectrum of the first peak in the TIC. C) Magnified plot of the deconvoluted mass spectrum of the first peak showing **mThfA17^{PanBC,ThfB}** with up to three dehydrations of the protein mass, indicative of the formation of the lasso peptide isopeptide bond and two ester crosslinks. D) Magnified plot of the deconvoluted mass spectrum of the first peak showing **mThfA17^{ThfB}** with up to two dehydrations of the protein mass, indicative of the formation of up to two ester crosslinks. The pink circle represents the pandonodin leader peptide and the gray circle in the cartoon represents the ThfA leader peptide.

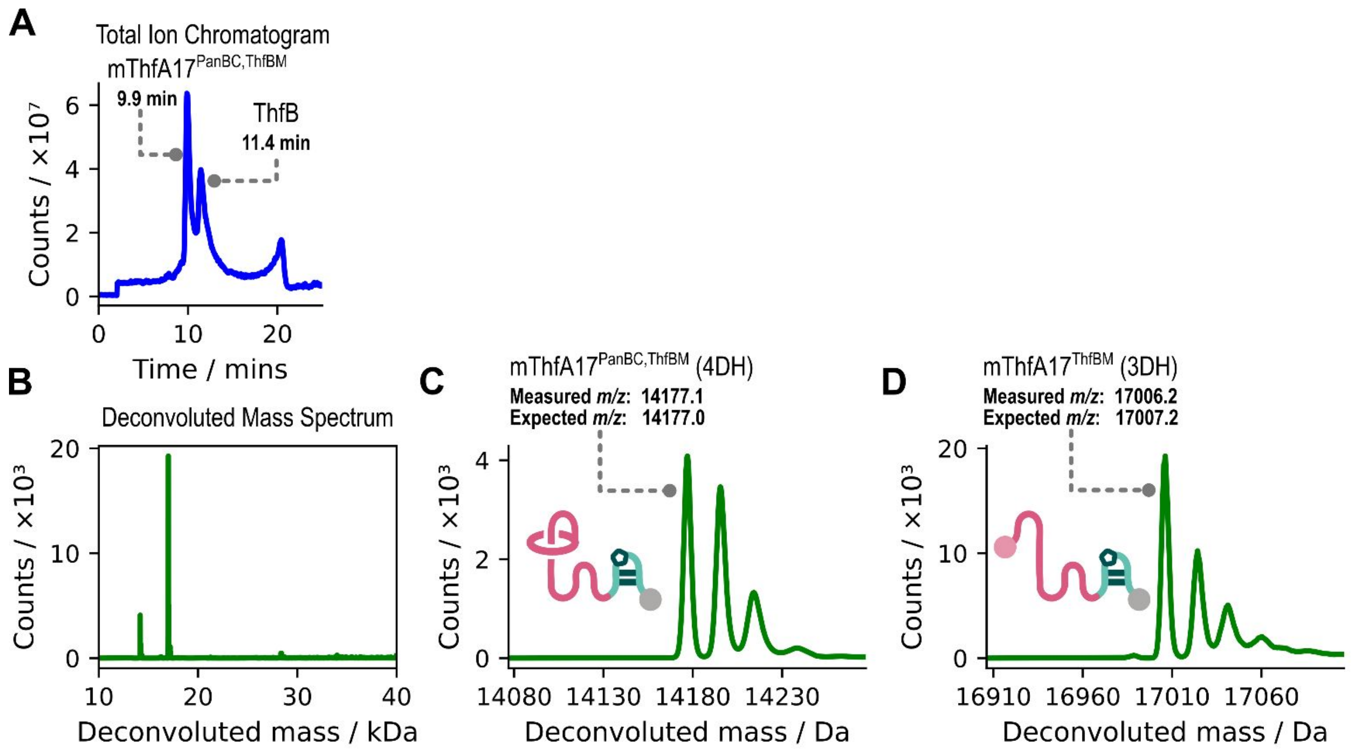

Figure S34. Mass spectrometry analysis of **mThfA17^{PanBC,ThfBM}** expressed in *E. coli* BL21 (DE3) *ΔslyD* cells transformed with pTGJ031 and pTGJ009, purified from the cell pellet under denaturing conditions. A) The total ion chromatogram of purified protein, with the retention time of the peaks labelled. B) Deconvoluted mass spectrum of the first peak in the TIC. C) Magnified plot of the deconvoluted mass spectrum of the first peak showing **mThfA17^{PanBC,ThfBM}** with up to four dehydrations of the protein mass, indicative of the formation of the lasso peptide isopeptide bond, two ester crosslinks and the aspartimidylation. D) Magnified plot of the deconvoluted mass spectrum of the first peak showing **mThfA17^{ThfBM}** with up to three dehydrations of the protein mass, indicative of the formation of two ester crosslinks and the aspartimidylation. The pink circle represents the pandonodin leader peptide and the gray circle in the cartoon represents the ThfA leader peptide.

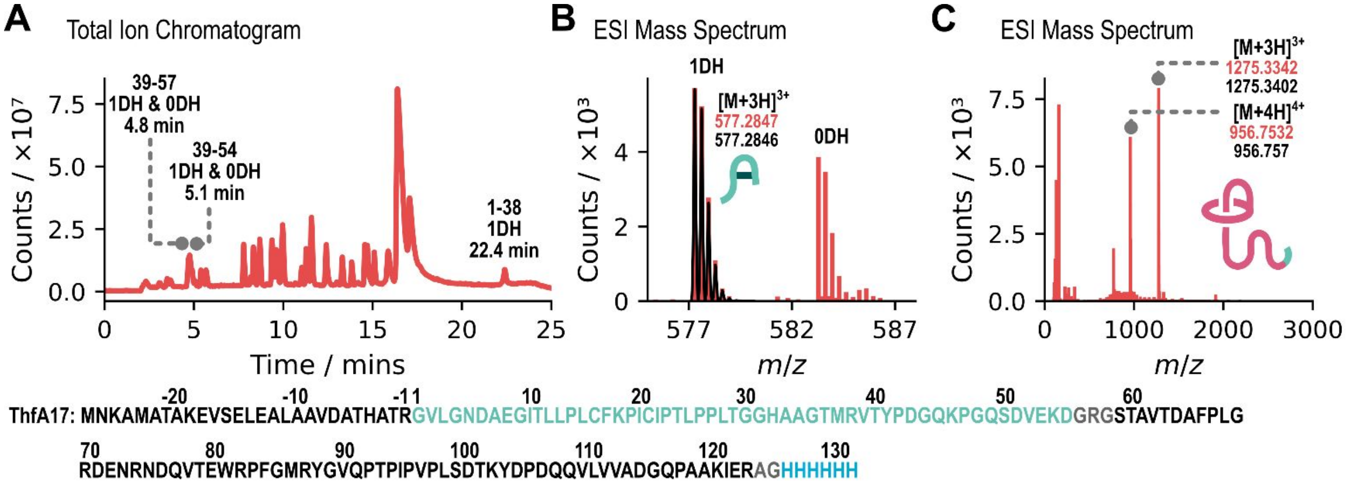

Figure S35. Mass spectrometry analysis of **mThfA17^{PanBC,ThfB}** trypsin digestion. A) The total ion chromatogram of trypsin digestion products following incubation overnight at 37 °C, with the annotations and retention time of the dehydrated product peaks labeled. B) Magnified plot of the ESI mass spectrum showing the zero and singly dehydrated 39-54 aa fragment $[M+3H]^{3+}$ ions with the simulated isotopic distribution shown as a black line for the singly dehydrated fragment. Observation of these fragments confirms the presence of the inner ester crosslink between Thr40-Asp51. C) Electrospray ionization mass spectrum of the 1-38 (1DH) peak in the TIC indicative of the lasso peptide fragment with the native Gly1-Glu8 isopeptide bond, where the top values (red) are the experimentally observed monoisotopic peak *m/z* value and the bottom values (black) are the theoretical *m/z* value. The numbered peptide sequence for ThfA14 is shown for clarity.

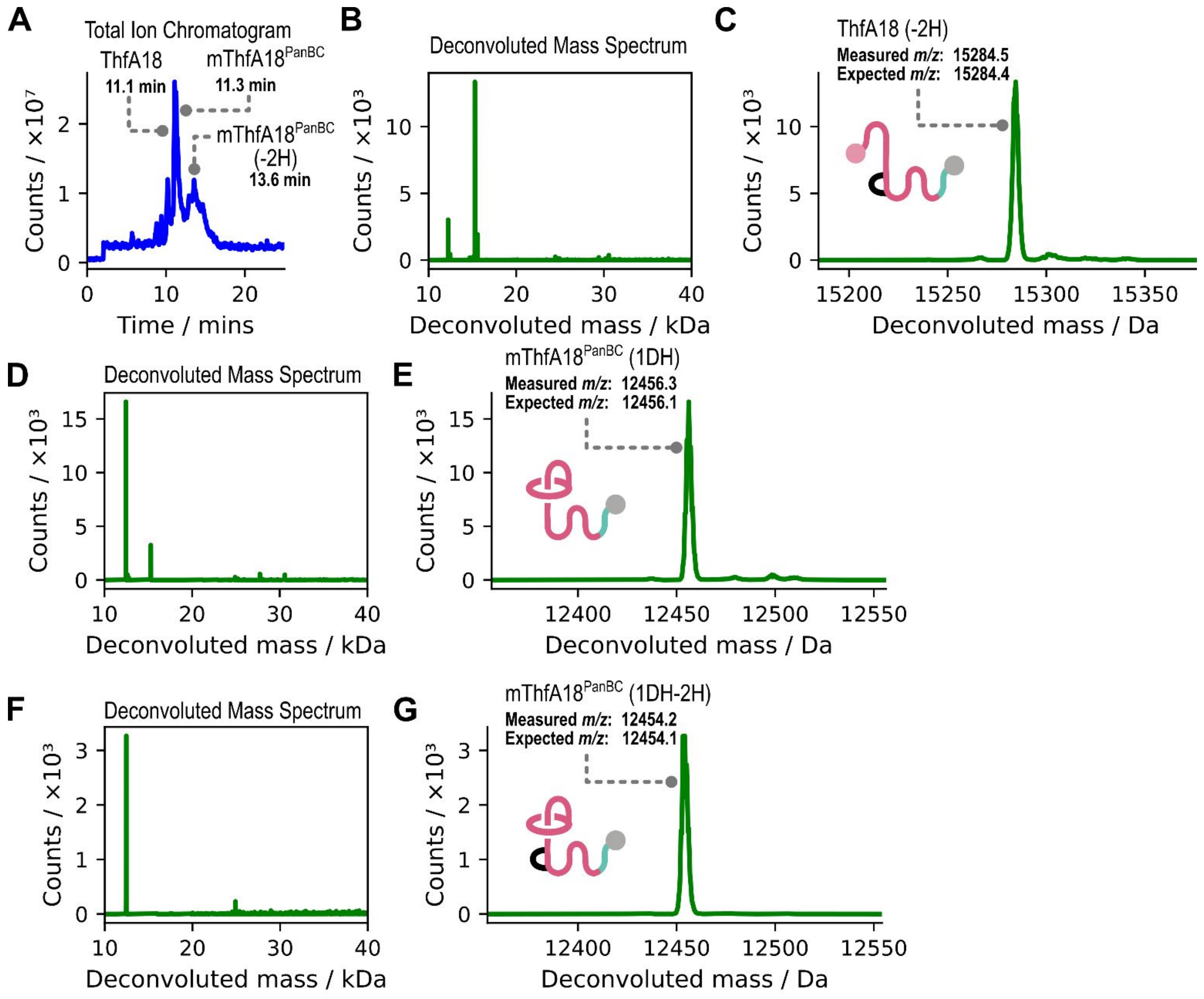

Figure S36. Mass spectrometry analysis of **mThfA18^{PanBC}** expressed in *E. coli* BL21 (DE3) *ΔslyD* cells transformed with pTGJ021, purified from the cell pellet under denaturing conditions. A) The total ion chromatogram of purified protein, with the retention times of the peaks labelled. B) Deconvoluted mass spectrum of the first peak in the TIC. C) Magnified plot of the deconvoluted mass spectrum of the first peak showing the unmodified protein mass of **ThfA18** with loss of 2 hydrogens, indicative of the formation of a disulfide bond. The pink circle represents the pandonodin leader peptide and the gray circle in the cartoon represents the ThfA leader peptide. D) Deconvoluted mass spectrum of the second peak in the TIC. E) Magnified plot of the deconvoluted mass spectrum of the second peak showing **mThfA18^{PanBC}** with one dehydration of the protein mass, indicative of the formation of the lasso peptide isopeptide bond. F) Deconvoluted mass spectrum of the third peak in the TIC. E) Magnified plot of the deconvoluted mass spectrum of the third peak showing **mThfA18^{PanBC}** with loss of 2 hydrogens and one dehydration of the protein mass, indicative of the formation of a disulfide bond and the lasso peptide isopeptide bond.

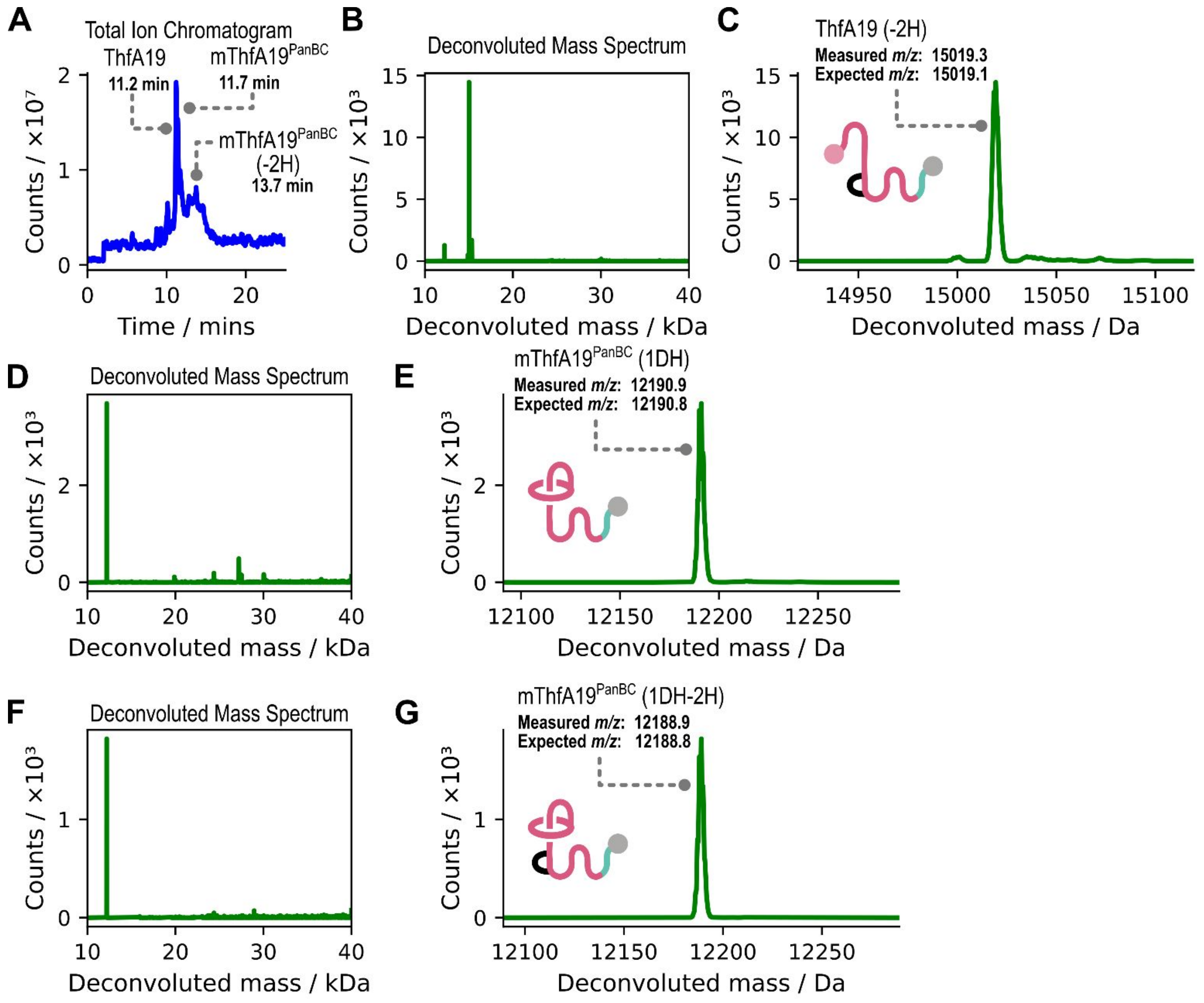

Figure S37. Mass spectrometry analysis of **mThfA19^{PanBC}** expressed in *E. coli* BL21 (DE3) *ΔslyD* cells transformed with pTGJ044, purified from the cell pellet under denaturing conditions. A) The total ion chromatogram of purified protein, with the retention times of the peaks labelled. B) Deconvoluted mass spectrum of the first peak in the TIC. C) Magnified plot of the deconvoluted mass spectrum of the first peak showing the unmodified protein mass of **ThfA19** with loss of 2 hydrogens, indicative of the formation of a disulfide bond. The pink circle represents the pandonodin leader peptide and the gray circle in the cartoon represents the ThfA leader peptide. D) Deconvoluted mass spectrum of the second peak in the TIC. E) Magnified plot of the deconvoluted mass spectrum of the second peak showing **mThfA19^{PanBC}** with one dehydration of the protein mass, indicative of the formation of the lasso peptide isopeptide bond. F) Deconvoluted mass spectrum of the third peak in the TIC. E) Magnified plot of the deconvoluted mass spectrum of the third peak showing **mThfA19^{PanBC}** with loss of 2 hydrogens and one dehydration of the protein mass, indicative of the formation of a disulfide bond and the lasso peptide isopeptide bond.

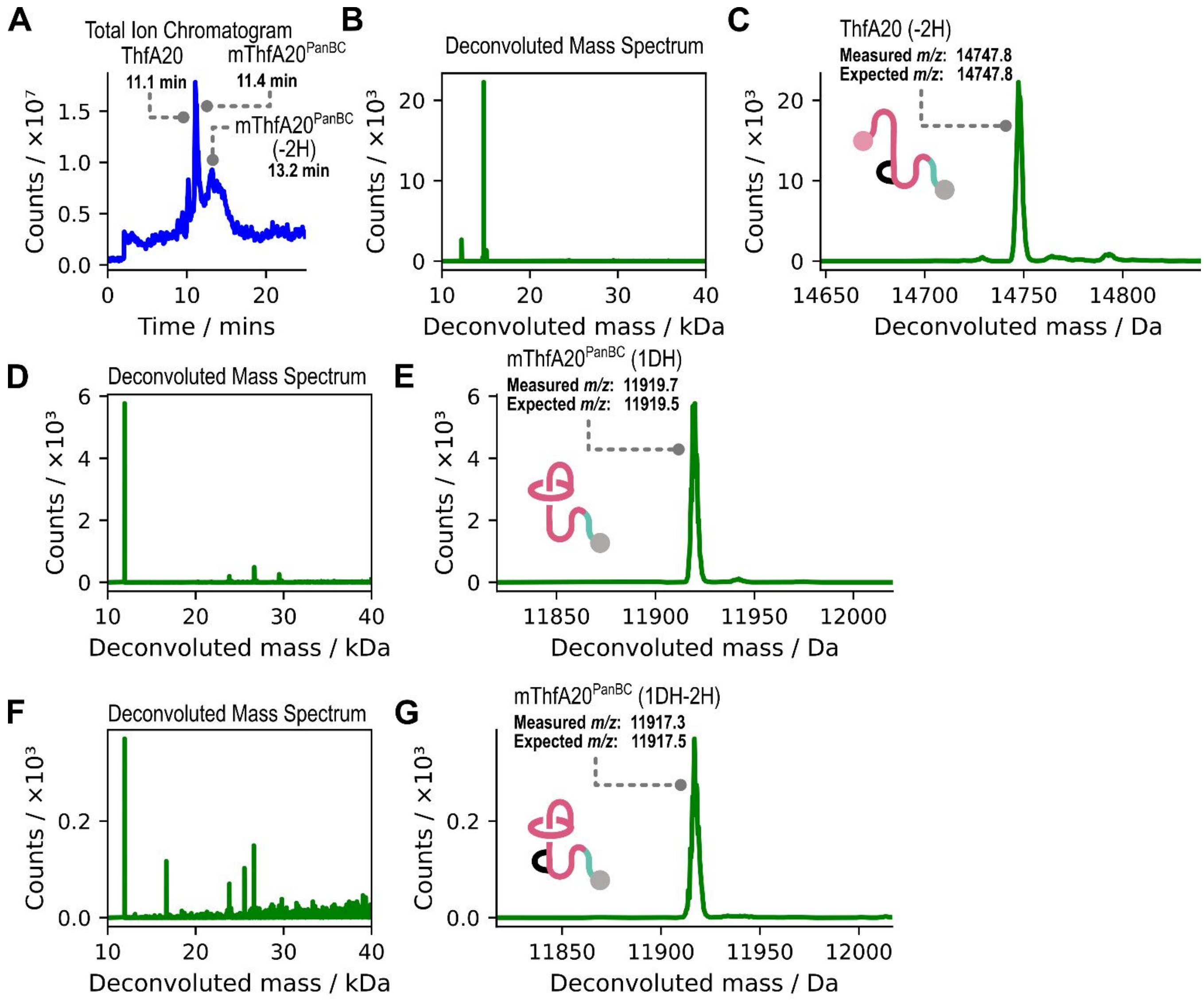

Figure S38. Mass spectrometry analysis of **mThfA20^{PanBC}** expressed in *E. coli* BL21 (DE3) *ΔslyD* cells transformed with pTGJ045, purified from the cell pellet under denaturing conditions. A) The total ion chromatogram of purified protein, with the retention times of the peaks labelled. B) Deconvoluted mass spectrum of the first peak in the TIC. C) Magnified plot of the deconvoluted mass spectrum of the first peak showing the unmodified protein mass of **ThfA20** with loss of 2 hydrogens, indicative of the formation of a disulfide bond. The pink circle represents the pandonodin leader peptide and the gray circle in the cartoon represents the ThfA leader peptide. D) Deconvoluted mass spectrum of the second peak in the TIC. E) Magnified plot of the deconvoluted mass spectrum of the second peak showing **mThfA20^{PanBC}** with one dehydration of the protein mass, indicative of the formation of the lasso peptide isopeptide bond. F) Deconvoluted mass spectrum of the third peak in the TIC. E) Magnified plot of the deconvoluted mass spectrum of the third peak showing **mThfA20^{PanBC}** with loss of 2 hydrogens and one dehydration of the protein mass, indicative of the formation of a disulfide bond and the lasso peptide isopeptide bond.

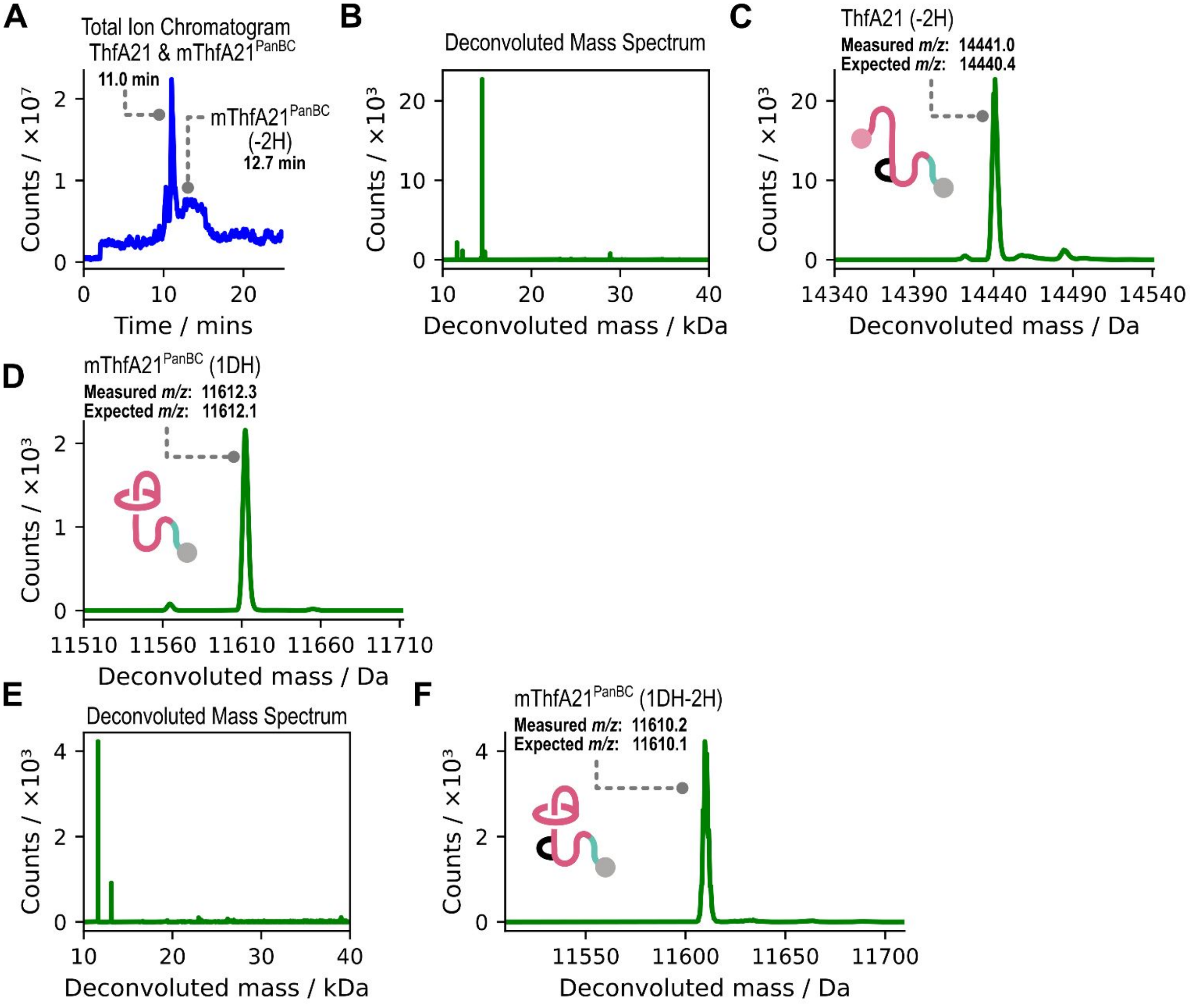

Figure S39. Mass spectrometry analysis of **mThfA21^{PanBC}** expressed in *E. coli* BL21 (DE3) *ΔslyD* cells transformed with pTGJ046, purified from the cell pellet under denaturing conditions. A) The total ion chromatogram of purified protein, with the retention times of the peaks labelled. B) Deconvoluted mass spectrum of the major peak in the TIC. C) Magnified plot of the deconvoluted mass spectrum of the major peak showing the unmodified protein mass of **ThfA21** with loss of 2 hydrogens, indicative of the formation of a disulfide bond. The pink circle represents the pandonodin leader peptide and the gray circle in the cartoon represents the ThfA leader peptide. D) Magnified plot of the deconvoluted mass spectrum of the major peak showing **mThfA21^{PanBC}** with one dehydration of the protein mass, indicative of the formation of the lasso peptide isopeptide bond. E) Deconvoluted mass spectrum of the minor peak in the TIC. E) Magnified plot of the deconvoluted mass spectrum of the minor peak showing **mThfA21^{PanBC}** with loss of 2 hydrogens and one dehydration of the protein mass, indicative of the formation of a disulfide bond and the lasso peptide isopeptide bond.

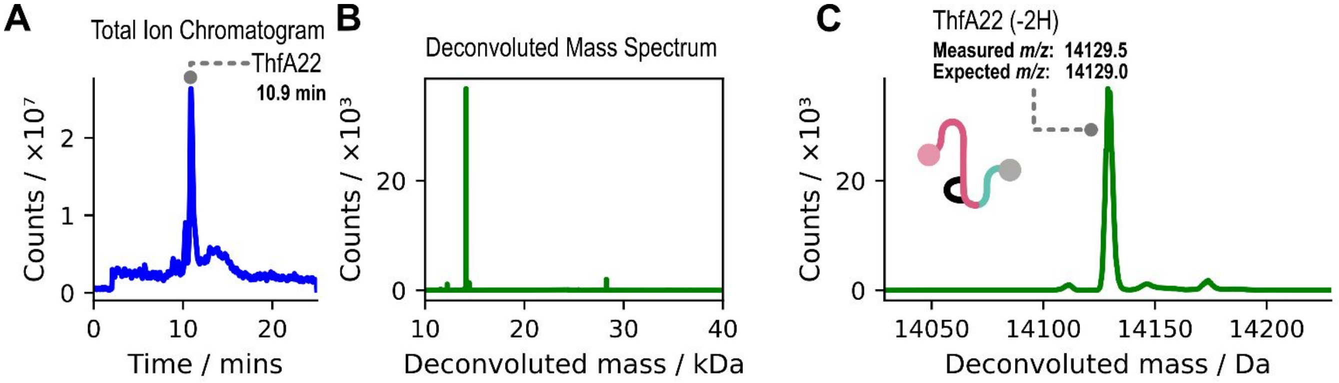

Figure S40. Mass spectrometry analysis of **ThfA22** expressed in *E. coli* BL21 (DE3) *ΔslyD* cells transformed with pTGJ047, purified from the cell pellet under denaturing conditions. A) The total ion chromatogram of purified protein, with the retention times of the peaks labelled. B) Deconvoluted mass spectrum of the major peak in the TIC. C) Magnified plot of the deconvoluted mass spectrum of the major peak showing the unmodified protein mass of **ThfA22** with loss of 2 hydrogens, indicative of the formation of a disulfide bond. No masses corresponding to **mThfA22$^{\mathbf{PanBC}}$** were detected, demonstrating that this variant could not be modified into a lasso peptide product when coexpressed with PanBCD. The pink circle represents the pandonodin leader peptide and the gray circle in the cartoon represents the ThfA leader peptide.

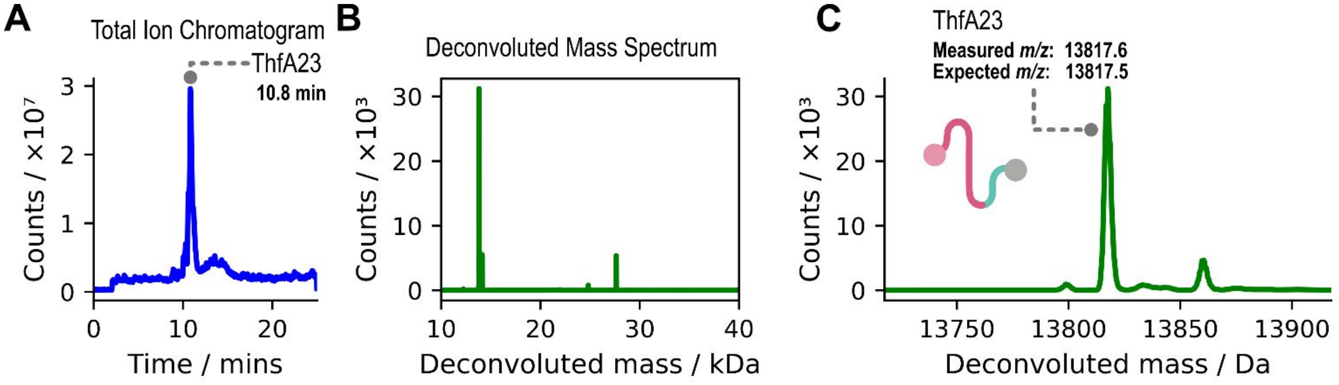

Figure S41. Mass spectrometry analysis of **ThfA23** expressed in *E. coli* BL21 (DE3) *ΔslyD* cells transformed with pTGJ048, purified from the cell pellet under denaturing conditions. A) The total ion chromatogram of purified protein, with the retention times of the peaks labelled. B) Deconvoluted mass spectrum of the major peak in the TIC. C) Magnified plot of the deconvoluted mass spectrum of the major peak showing the unmodified protein mass of **ThfA23**. No masses corresponding to **mThfA23$^{\mathbf{PanBC}}$** were detected, demonstrating that this variant could not be modified into a lasso peptide product when coexpressed with PanBCD. The pink circle represents the pandonodin leader peptide and the gray circle in the cartoon represents the ThfA leader peptide.

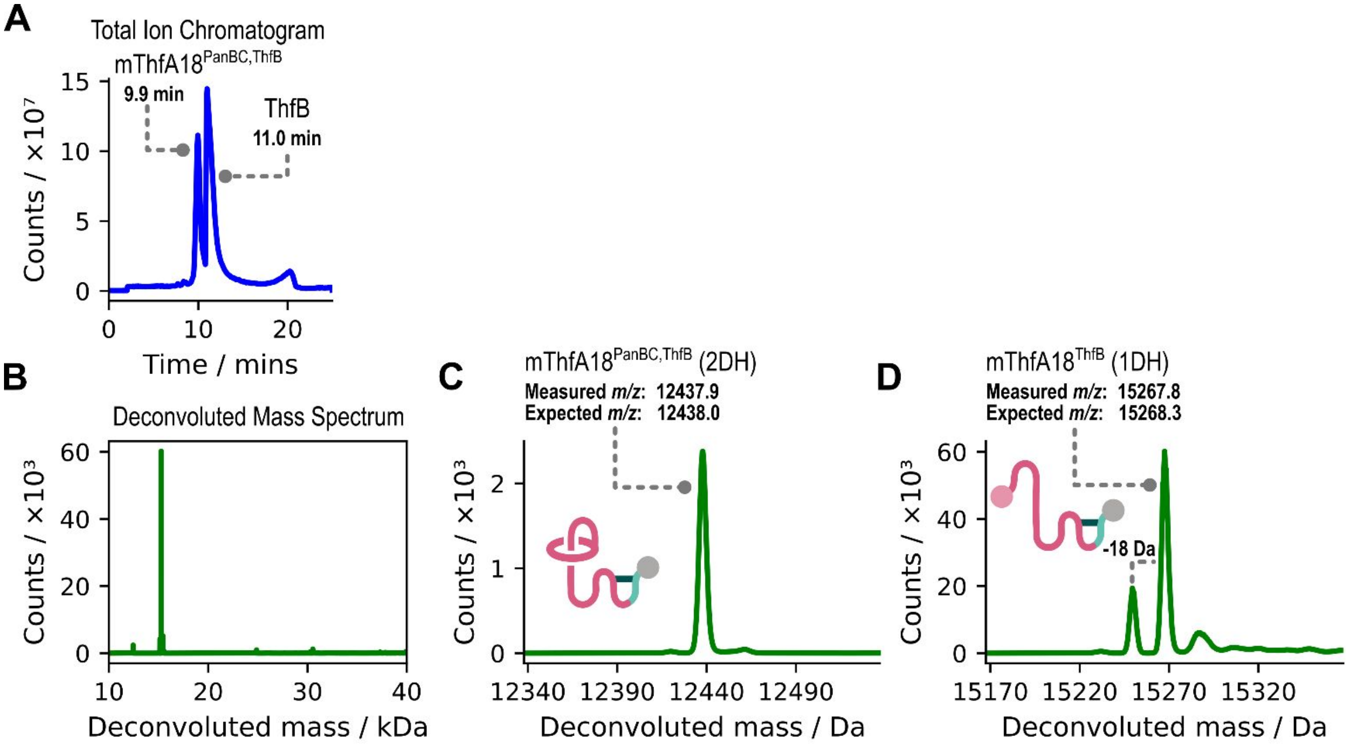

Figure S42. Mass spectrometry analysis of **mThfA18^{PanBC,ThfB}** expressed in *E. coli* BL21 (DE3) *ΔslyD* cells transformed with pTGJ021 and pBC262, purified from the cell pellet under native conditions. A) The total ion chromatogram of purified protein, with the retention times of the peaks labelled. B) Deconvoluted mass spectrum of the first peak in the TIC. C) Magnified plot of the deconvoluted mass spectrum of the first peak showing **mThfA18^{PanBC,ThfB}** with two dehydrations of the protein mass, indicative of the formation of the lasso peptide isopeptide bond and one ThfB-installed crosslink. D) Magnified plot of the deconvoluted mass spectrum of the first peak showing **mThfA18^{ThfB}** with one dehydration of the protein mass, indicative of the formation of one ThfB-installed crosslink. The pink circle represents the pandonodin leader peptide and the gray circle in the cartoon represents the ThfA leader peptide.

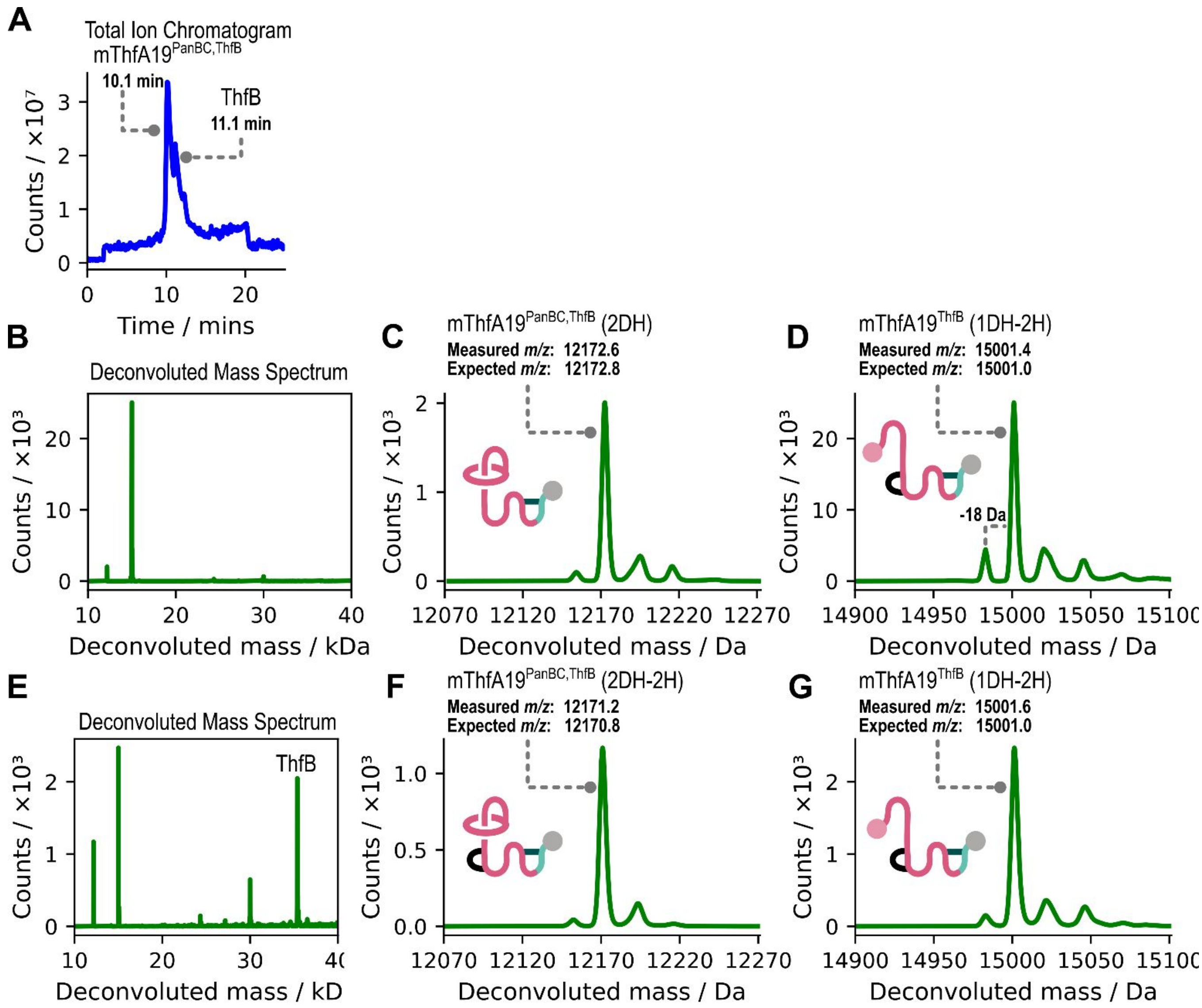

Figure S43. Mass spectrometry analysis of **mThfA19^{PanBC,ThfB}** expressed in *E. coli* BL21 (DE3) *ΔslyD* cells transformed with pTGJ044 and pBC262, purified from the cell pellet under denaturing conditions. A) The total ion chromatogram of purified protein, with the retention times of the peaks labelled. B) Deconvoluted mass spectrum of the first peak in the TIC. C) Magnified plot of the deconvoluted mass spectrum of the first peak showing **mThfA19^{PanBC,ThfB}** with two dehydrations of the protein mass, indicative of the formation of the lasso peptide isopeptide bond and one ThfB-installed crosslink. D) Magnified plot of the deconvoluted mass spectrum of the first peak showing **mThfA19^{ThfB}** with loss of 2 hydrogens and one dehydration of the protein mass, indicative of the formation of a disulfide bond and one ThfB-installed crosslink. The pink circle represents the pandonodin leader peptide and the gray circle in the cartoon represents the ThfA leader peptide. E) Deconvoluted mass spectrum of the second peak in the TIC. F) Magnified plot of the deconvoluted mass spectrum of the second peak showing **mThfA19^{PanBC,ThfB}** with loss of 2 hydrogens and two dehydrations of the protein mass, indicative of the formation of the lasso peptide isopeptide bond and one ThfB-installed crosslink. D)

Magnified plot of the deconvoluted mass spectrum of the first peak showing **mThfA19^{ThfB}** with loss of 2 hydrogens and one dehydration of the protein mass, indicative of the formation of a disulfide bond and one ThfB-installed crosslink.

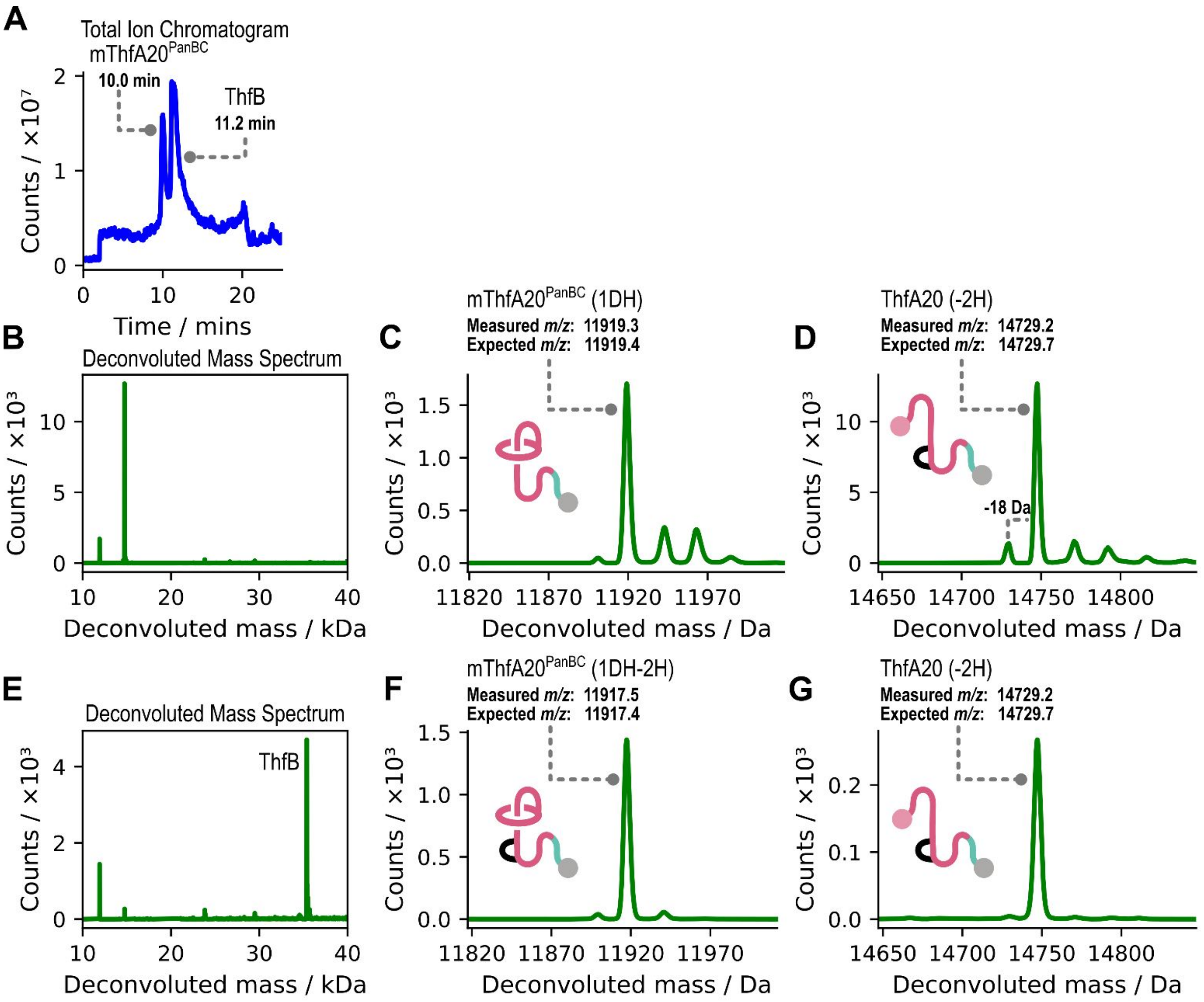

Figure S44. Mass spectrometry analysis of **mThfA20^{PanBC,ThfB}** expressed in *E. coli* BL21 (DE3) *ΔslyD* cells transformed with pTGJ045 and pBC262, purified from the cell pellet under denaturing conditions. A) The total ion chromatogram of purified protein, with the retention time of the peaks labelled. B) Deconvoluted mass spectrum of the first peak in the TIC. C) Magnified plot of the deconvoluted mass spectrum of the first peak showing **mThfA20^{PanBC}** with one dehydration of the protein mass, indicative of the formation of the lasso peptide isopeptide bond. D) Magnified plot of the deconvoluted mass spectrum of the first peak showing the unmodified protein mass of **ThfA20** with loss of 2 hydrogens, indicative of the formation of a disulfide bond. The pink circle represents the pandonodin leader peptide and the gray circle in the cartoon represents the ThfA

leader peptide. E) Deconvoluted mass spectrum of the second peak in the TIC. F) Magnified plot of the deconvoluted mass spectrum of the second peak showing **mThfA20^{PanBC}** with loss of 2 hydrogens and one dehydration of the protein mass, indicative of the formation of a disulfide bond and the lasso peptide isopeptide bond. G) Magnified plot of the deconvoluted mass spectrum of the first peak showing the unmodified protein mass of **ThfA20** with loss of 2 hydrogens, indicative of the formation of a disulfide bond.

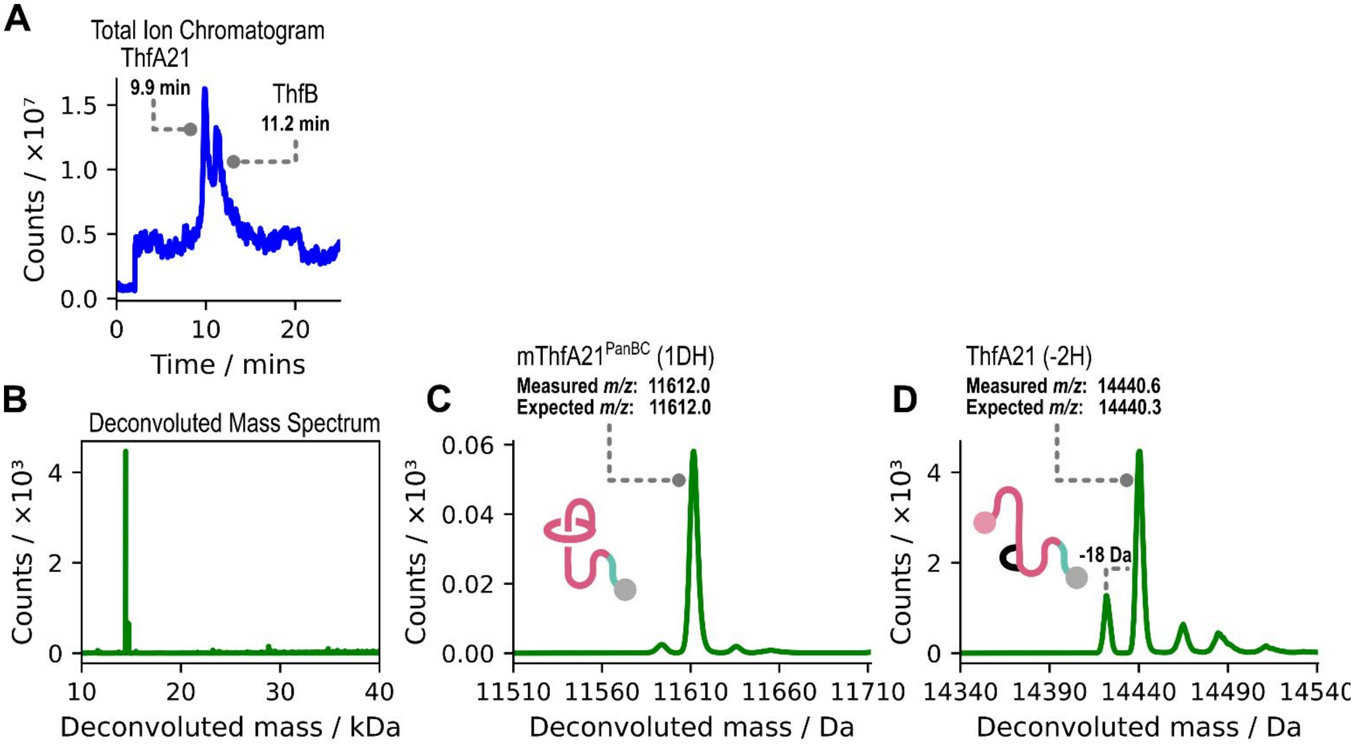

Figure S45. Mass spectrometry analysis of **mThfA21^{PanBC,ThfB}** expressed in *E. coli* BL21 (DE3) *ΔslyD* cells transformed with pTGJ046 and pBC262, purified from the cell pellet under denaturing conditions. A) The total ion chromatogram of purified protein, with the retention time of the peaks labelled. B) Deconvoluted mass spectrum of the first peak in the TIC. C) Magnified plot of the deconvoluted mass spectrum of the first peak showing **mThfA21^{PanBC}** with one dehydration of the protein mass, indicative of the formation of a lasso peptide isopeptide bond. D) Magnified plot of the deconvoluted mass spectrum of the first peak showing the unmodified protein mass of **ThfA21** with loss of 2 hydrogens, indicative of the formation of a disulfide bond. The pink circle represents the pandonodin leader peptide and the gray circle in the cartoon represents the ThfA leader peptide.

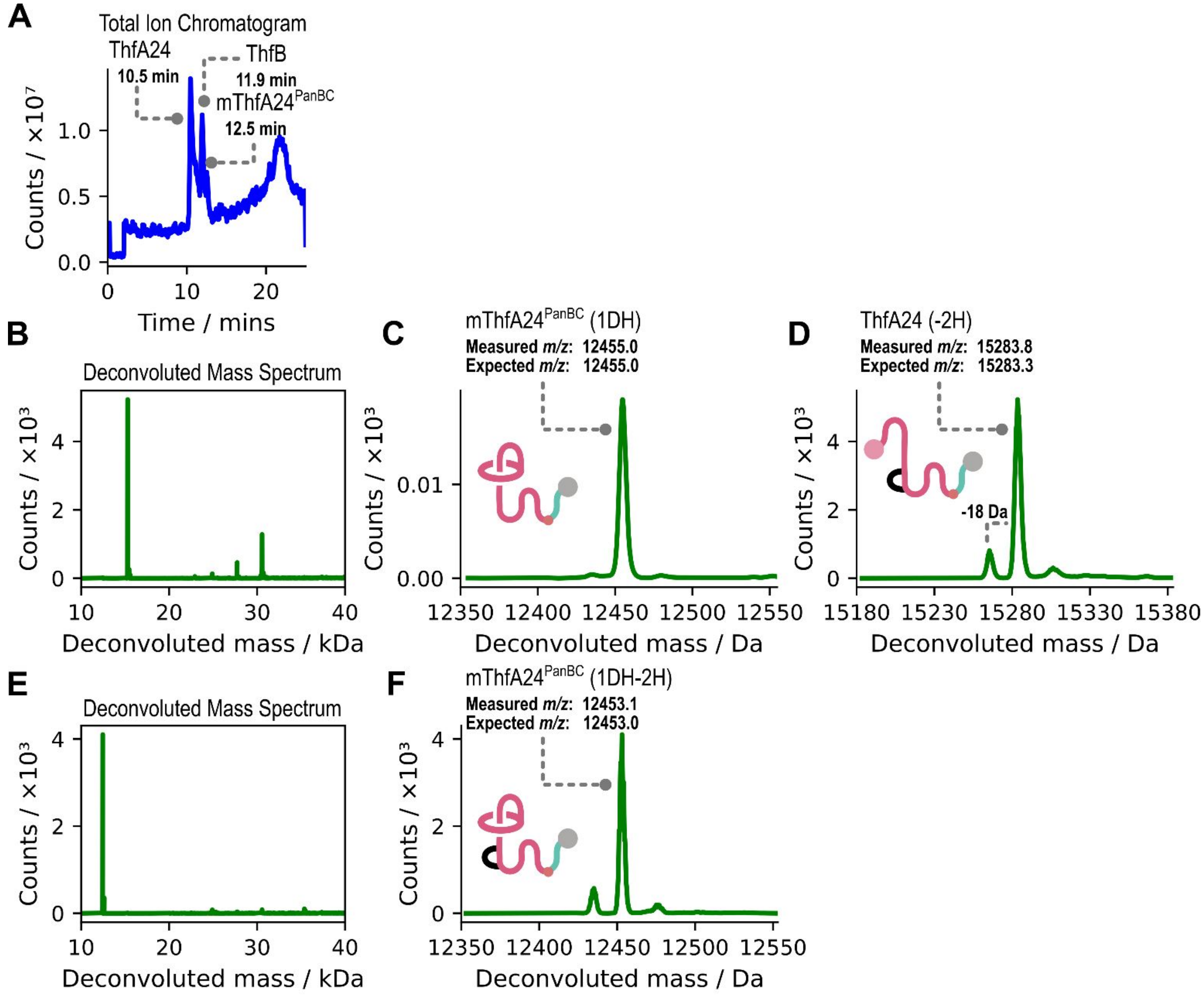

Figure S46. Mass spectrometry analysis of **mThfA24^{PanBC,ThfB}** expressed in *E. coli* BL21 (DE3) *ΔslyD* cells transformed with pTGJ070 and pBC262, purified from the cell pellet under denaturing conditions. A) The total ion chromatogram of purified protein, with the retention times of the peaks labelled. B) Deconvoluted mass spectrum of the first peak in the TIC. C) Magnified plot of the deconvoluted mass spectrum of the first peak showing **mThfA24^{PanBC}** with one dehydration of the protein mass, indicative of the formation of a lasso peptide isopeptide bond. D) Magnified plot of the deconvoluted mass spectrum of the first peak showing the unmodified protein mass of **ThfA24** with loss of 2 hydrogens, indicative of the formation of a disulfide bond. E) Deconvoluted mass spectrum of the third peak (12.5 min) in the TIC. F) Magnified plot of the deconvoluted mass spectrum of the third peak (12.5 min) showing **mThfA24^{PanBC}** with loss of 2 hydrogens and one dehydration of the protein mass, indicative of the formation of a disulfide bond and the lasso peptide isopeptide bond. The large pink circle represents the pandonodin leader peptide and the large gray circle in the cartoon represents the ThfA leader peptide, while the smaller red circle represents a point substitution (Asp34Asn).

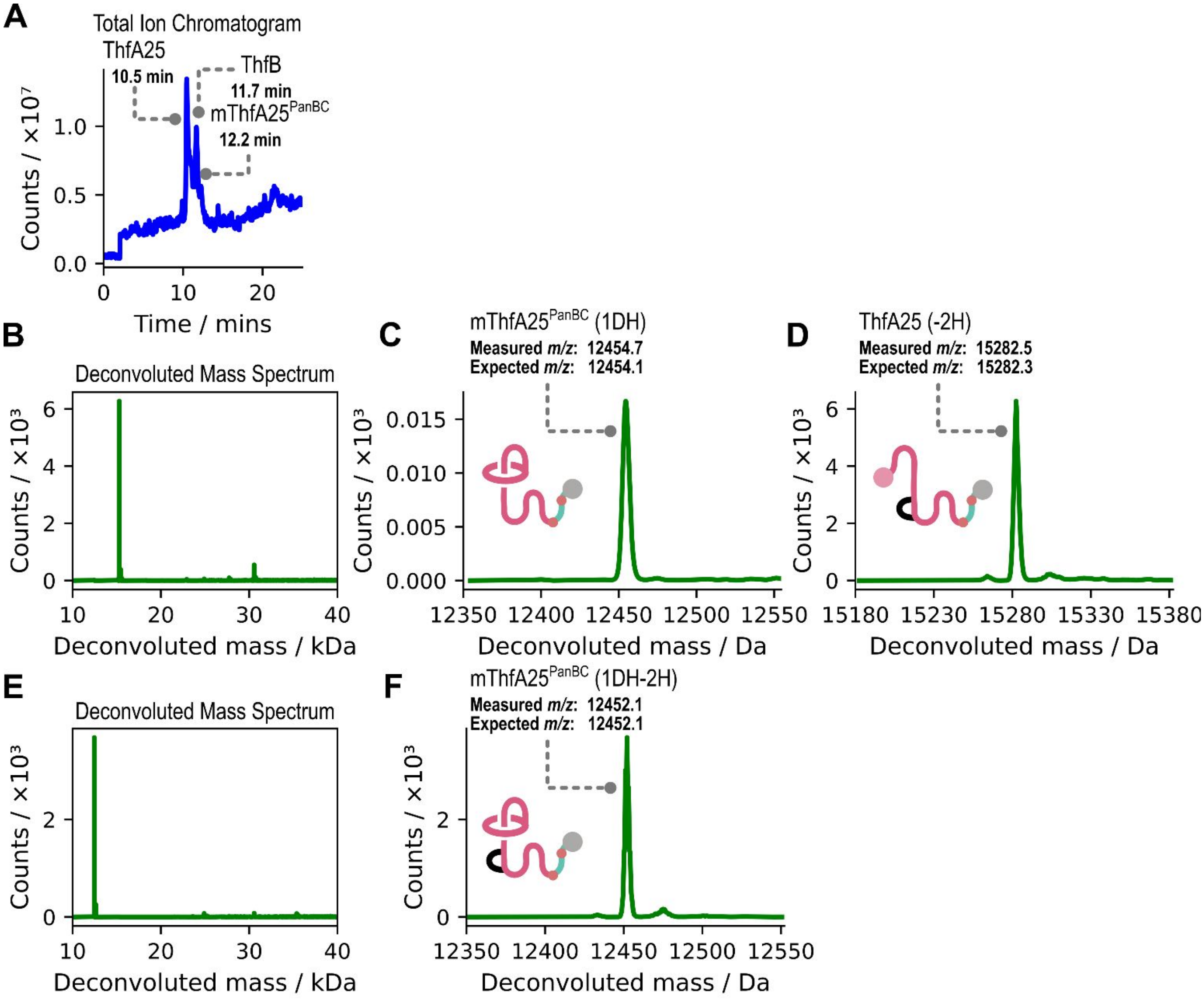

Figure S47. Mass spectrometry analysis of **mThfA25$^{\mathbf{PanBC,ThfB}}$** expressed in *E. coli* BL21 (DE3) *ΔslyD* cells transformed with pTGJ071 and pBC262, purified from the cell pellet under denaturing conditions. A) The total ion chromatogram of purified protein, with the retention times of the peaks labelled. B) Deconvoluted mass spectrum of the first peak in the TIC. C) Magnified plot of the deconvoluted mass spectrum of the first peak showing **mThfA25$^{\mathbf{PanBC}}$** with one dehydration of the protein mass, indicative of the formation of a lasso peptide isopeptide bond. D) Magnified plot of the deconvoluted mass spectrum of the first peak showing the unmodified protein mass of **ThfA25** with loss of 2 hydrogens, indicative of the formation of a disulfide bond. E) Deconvoluted mass spectrum of the third peak (12.2 min) in the TIC. F) Magnified plot of the deconvoluted mass spectrum of the third peak (12.2 min) showing **mThfA25$^{\mathbf{PanBC}}$** with loss of 2 hydrogens and one dehydration of the protein mass, indicative of the formation of a disulfide bond and the lasso peptide isopeptide bond. The large pink circle represents the pandonodin leader peptide and the large gray circle in the cartoon represents the ThfA leader peptide, while the smaller red circles represent point substitutions (Asp34Asn and Asp38Asn).

References for Supplemental Information

1. Choi, B., Elashal, H. E., Cao, L. & Link, A. J. Mechanistic Analysis of the Biosynthesis of the Aspartimidylated Graspetide Amycolimiditide. *J Am Chem Soc* **144**, 21628–21639 (2022).

2. Niedermeyer, T. H. J. & Strohalm, M. mMass as a Software Tool for the Annotation of Cyclic Peptide Tandem Mass Spectra. *PLoS One* **7**, e44913 (2012).

3. Elashal, H. E. *et al.* Biosynthesis and characterization of fuscimiditide, an aspartimidylated graspetide. *Nature Chemistry 2022 14:11* **14**, 1325–1334 (2022).

4. Choi, B., Johnson, T. G., Acuña, A., Elashal, H. E. & Link, A. J. Peptide and Protein Cyclization by a Promiscuous Graspetide Synthetase. *ACS Cent Sci* **2025**, 1111–1121 (2025).

5. Cheung-Lee, W. L., Cao, L. & Link, A. J. Pandonodin: A Proteobacterial Lasso Peptide with an Exceptionally Long C-Terminal Tail. *ACS Chem Biol* **14**, 2783–2792 (2019).
